# Accelerating the discovery of alkyl halide-derived natural products using halide depletion

**DOI:** 10.1038/s41557-023-01390-z

**Published:** 2024-01-12

**Authors:** Nathaniel R. Glasser, Dongtao Cui, Douglas D. Risser, C. Denise Okafor, Emily P. Balskus

**Affiliations:** 1 Department of Chemistry and Chemical Biology, Harvard University, Cambridge, MA USA; 2 Department of Biology, University of the Pacific, Stockton, CA USA; 3 Department of Biochemistry and Molecular Biology, Pennsylvania State University, University Park, PA USA; 4 Department of Chemistry, Pennsylvania State University, University Park, PA USA; 5 Howard Hughes Medical Institute, Harvard University, Cambridge, MA USA

**Keywords:** Natural products, Metabolomics

## Abstract

Even in the genomic era, microbial natural product discovery workflows can be laborious and limited in their ability to target molecules with specific structural features. Here we leverage an understanding of biosynthesis to develop a workflow that targets the discovery of alkyl halide-derived natural products by depleting halide anions, a key biosynthetic substrate for enzymatic halogenation, from microbial growth media. By comparing the metabolomes of bacterial cultures grown in halide-replete and deficient media, we rapidly discovered the nostochlorosides, the products of an orphan halogenase-encoding gene cluster from *Nostoc punctiforme* ATCC 29133. We further found that these products, a family of unusual chlorinated glycolipids featuring the rare sugar gulose, are polymerized via an unprecedented enzymatic etherification reaction. Together, our results highlight the power of leveraging an understanding of biosynthetic logic to streamline natural product discovery.

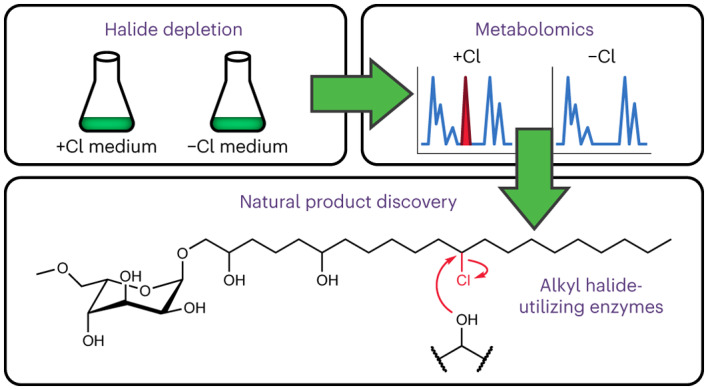

## Abstract (web summary)

Natural products discovery remains an ongoing challenge. Now, halide depletion offers a complementary approach to discover natural products whose biosynthesis requires halides, including products of cryptic halogenation. Halide depletion reveals that nostochlorosides, the products of an orphan biosynthetic gene cluster in *Nostoc punctiforme*, are polymerized by a halide-displacing etherifying enzyme.

## Main

Natural products are an important source of both molecular complexity and bioactive pharmacophores^1^, and elucidating natural product biosynthetic pathways has led to the discovery of previously unknown enzymatic chemistry^2^. Although analyses of microbial genomes have uncovered a wealth of uncharacterized natural product biosynthetic gene clusters (BGCs)^3^, the discovery of new natural products from these organisms is still hampered by challenges such as redundancy and re-discovery^4^. Many genomics-driven natural product discovery approaches also require genetic manipulation of either a genetically tractable producing organism or a heterologous host^5,6^, which can be laborious and challenging. Clearly, accelerating access to microbial natural products will require the development of innovative approaches designed to target the discovery of novel compounds.

Halogenated natural products are an important class of metabolites that provide exciting opportunities for discovery. In pharmacology, halogen atoms (F, Cl, Br and I) alter the physicochemical properties of small molecules and can increase membrane permeability^7^. Approximately 25% of drugs and drug candidates contain at least one halogen atom^8^, a testament to the utility of halogens for modulating bioactivity and pharmacokinetics. Halogen atoms are introduced into metabolites by biosynthetic enzymes called halogenases^9^, and at least 5,000 halogenated natural products are known so far^10^. In addition to their pivotal functions in natural product biosynthesis, halogenases also show promise as stereo- and regioselective biocatalysts for small-molecule synthesis^11^. Accordingly, methods to streamline the discovery of halogenated natural products—and their associated biosynthetic machinery—have facilitated the identification of useful compounds and enzymes. Bioinformatically, the sequences of halogenase enzymes^9^ have guided the selection of strains for genome-mining approaches^12^. Mass spectrometry (MS) is also a powerful tool for identifying new halogenated metabolites, with approaches developed so far having relied on identifying the characteristic isotope distribution of chlorine and bromine^13^, fragmentation analysis to detect halide daughter ions^14^, and elemental analysis to detect halogens directly^15^. Halogens therefore provide a useful handle to identify biologically and chemically interesting natural products.

**Fig. 1:**
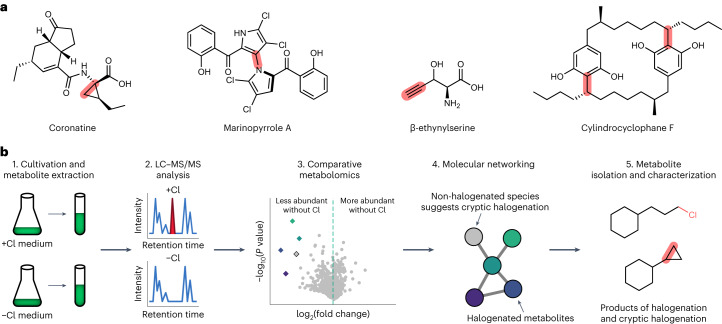
Halide depletion as a tool for natural product discovery. **a**, Examples of natural products derived from cryptic halogenation. The bonds highlighted in red are formed by alkyl halide-derivatizing enzymes that use halogenated intermediates to enable C–C and C–N bond formation. **b**, Overview of our halide depletion workflow. Microbial cultures are grown with and without a halide (such as chloride) present in the growth medium. Metabolite extracts are analysed by LC–MS/MS. Comparative metabolomics and molecular networking reveal key differences between the two cultures, with metabolites derived from halogenation being significantly less abundant in halide-depleted cultures. If a non-halogenated metabolite’s abundance is halide-dependent, then clustering of the non-halogenated metabolite (grey) with halogenated metabolites (coloured) in a molecular network may suggest a role for cryptic halogenation in biosynthesis.

A particularly intriguing role for halogenated metabolites is their use as precursors in the biosynthesis of more complex molecules. For example, cryptic halogenation is a biosynthetic strategy whereby an intermediate is transiently halogenated to activate it for further enzymatic chemistry^16^. Examples of the diverse structural motifs derived from cryptic halogenation include cyclopropanes^17,18^, biaryls^19^, terminal alkynes^20^ and paracyclophanes^21,22^ (Fig. 1a). Unlike their corresponding halogenated biosynthetic precursors, natural products derived from cryptic halogenation are often unhalogenated and are therefore difficult to discover by existing MS-based methods. Moreover, the alkyl halide-derivatizing enzymes involved in cryptic halogenation come from diverse protein families that are insufficiently characterized compared to their halogenase counterparts^16^, complicating efforts to identify such pathways using bioinformatics. Given the diversity of unusual scaffolds known to be produced through cryptic halogenation, methods to identify such products would be particularly valuable for natural product discovery.

In this Article we use an understanding of biosynthesis to accelerate the discovery of natural products and biosynthetic pathways that involve halogenation. Recognizing that halide anions are essential substrates for halogenases, we explored whether removing them from microbial growth media (‘halide depletion’) perturbs the production of halogenated natural products and metabolites derived from cryptic halogenation. As a proof of concept, we first combined halide depletion with comparative metabolomics and molecular networking (Fig. 1b) to identify new paracyclophane derivatives and biosynthetic precursors from cyanobacteria. We then applied this workflow to discover a family of chlorinated glycolipids from *Nostoc punctiforme* ATCC 29133 produced by an orphan BGC (*pks3*). Our findings highlight the potential of halide depletion, as well as other strategies that leverage biosynthetic knowledge, to dramatically streamline natural product and enzyme discovery.

## Halide depletion reveals new cylindrocyclophane derivatives

**Fig. 2:**
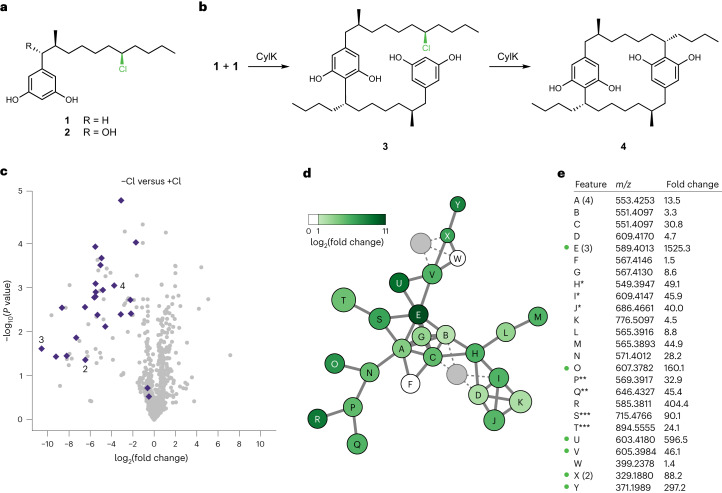
Discovery of new cylindrocyclophane derivatives validates the halide depletion workflow. **a**, Monoalkylresorcinol monomers. **b**, The cylindrocyclophane biosynthetic pathway involves the stepwise dimerization of the monoalkylresorcinol **1** to form a cylindrofridin (**3**) and cylindrocyclophane (**4**). **c**, LC–MS analysis of *C. licheniforme* ATCC 29412. Fold changes represent mean feature abundance in cultures grown without chloride relative to those grown with chloride (three independent biological replicates per condition), and *P* values were determined by a two-tailed Student’s *t*-test. Diamonds represent features identified in the cylindrocyclophane metabolic network. Circles represent other features. **d**, The cylindrocyclophane metabolic network. Each circle represents a unique feature from the LC–MS data, and the circle area is proportional to the *m*/*z* value (*z* = 1 for all features). Solid lines represent feature similarity with a cosine score of at least 0.60 and at least four matching fragments or neutral losses in MS/MS spectra. Colours represent the fold change of the feature as shown in **c**. The two features in grey, connected to the network by dashed lines, represent artefacts from the automated MS/MS procedure that were not identified as real features in **c**. **e**, A list of measured *m*/*z* values (*z* = 1 for all features, positive ionization) of the features in the cylindrocyclophane metabolic network and their associated fold changes. Green dots represent chlorinated features as determined by the intensity of the M + 2 isotopologue in MS1 spectra. Asterisks (*, ** and ***) represent groups of features that probably originate from the same parent compound based on nearly identical retention times (Supplementary Table 1).

The development of our halide depletion discovery workflow was inspired by several isolated studies that suggested that levels of halide ions in bacterial growth media can influence the production of known natural products derived from enzymatic halogenation^23,24^. We first sought to validate our halide depletion workflow in the cyanobacterium *Cylindrospermum licheniforme* ATCC 29412. This organism produces the cylindrocyclophanes, a family of paracyclophane natural products (Supplementary Fig. 1a) that are constructed via cryptic halogenation^21,22^. Cylindrocyclophane biosynthesis involves the stepwise dimerization of a halogenated monoalkylresorcinol precursor (**1**) (Fig. 2a) by the alkyl halide-derivatizing enzyme CylK, which catalyses a Friedel–Crafts alkylation (Fig. 2b)^22,25^. The halogenated alkylresorcinol precursors (for example, **2**) are known as cylindrofridins (Supplementary Fig. 1b) and are also abundant metabolites in some cylindrocyclophane-producing species^26^. Robust cylindrocyclophane production occurs in both chloride- and bromide-containing media^17^, suggesting that the biosynthetic machinery can use either halide. Given that cylindrocyclophane biosynthesis requires a halide to generate the key halogenated intermediate, we reasoned that cylindrocyclophane production should be greatly reduced or abolished upon halide depletion.

To test this hypothesis, we compared the metabolomes of *C. licheniforme* cultures grown in minimal medium with and without chloride, where the two media had similar osmolarity, ionic strength and concentrations of other nutrients. Comparison of biomass extracts by LC–MS identified a number of differentially abundant features between the two conditions (Fig. 2c and Supplementary Table 1). A feature consistent with a cylindrocyclophane biosynthetic intermediate (**3**) (Fig. 2b) represented the largest depletion, with a relative fold change of 1,525 (*P* = 2.4 × 10^−2^). Features assigned to cylindrocyclophane F (**4**) and the hydroxymonoalkylresorcinol monomer (**2**) were also depleted in cultures grown without chloride, with relative fold changes of 13.5 (*P* = 8.9 × 10^−4^) and 88.2 (*P* = 4.3 × 10^−2^), respectively.

To better understand potential structural relationships between the metabolites, we next re-analysed the samples using tandem liquid chromatography–mass spectrometry (LC–MS/MS) and analysed the spectra by molecular networking. The molecular networking identified 22 features that clustered with cylindrocyclophane F (Fig. 2d). All but two of these features showed a lower abundance in cultures grown without chloride (Fig. 2c,d), and only four appeared to be halogenated (Supplementary Fig. 2), suggesting that most of the features may arise from cryptic halogenation. Indeed, the formulae predicted for each feature are consistent with known and previously unknown cylindrocyclophanes or cylindrofridins (Supplementary Fig. 1c). Database searches of METLIN^27^ and GNPS^28^ did not suggest plausible identities for the other features that responded to halide depletion but did not cluster with the cylindrocyclophane metabolic network, further emphasizing the need for methods to illuminate features of interest in complex datasets. Together, these data indicate that halide depletion can enable the facile identification of compounds derived from halogenation in complex microbial metabolomes.

## Halide depletion reveals new halogenated natural products

**Fig. 3:**
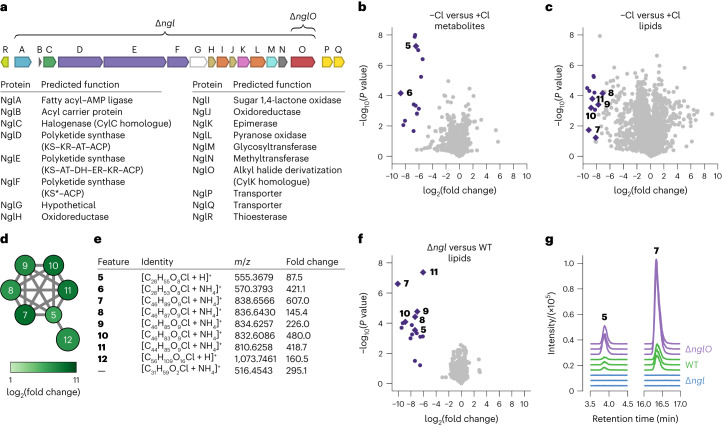
Discovery of *ngl*-derived metabolites in *N. punctiforme* ATCC 29133 using halide depletion. **a**, The CylC- and CylK-encoding BGC (*ngl*) from *N. punctiforme* ATCC 29133. The *ngl* BGC represents 36,029 bp comprising the genes Npun_R3355 to Npun_F3373 (NPUN_RS17000 to NPUN_RS17085). Predicted functions are based on annotations from the Conserved Domain Database. KS, ketosynthase; KR, ketoreductase; AT, acyltransferase; DH, dehydrogenase; ER, enoylreductase; ACP, acyl carrier protein; KS*, ketosynthase with an inactivating point mutation in an active-site residue. Brackets indicate the genomic regions deleted to generate strains ∆*ngl* and ∆*nglO*. **b**,**c**, LC–MS analysis of *N. punctiforme* ATCC 29133 cultures grown with and without chloride: features detected using the same chromatographic separation used for cylindrocyclophanes (**b**) and additional features detected using a chromatographic separation optimized for lipids (**c**). Fold changes represent mean feature abundance in cultures grown without chloride relative to those grown with chloride (three independent biological replicates per condition), and *P* values were determined by a two-tailed Student’s *t*-test. Purple diamonds represent the parent ion of features of interest. Purple circles represent features originating from alternative ion adducts or source fragment artefacts. Grey circles represent other features. **d**, The metabolic network of features identified in **b** and **c**. Each circle represents a unique feature from the LC–MS data, with the circle area proportional to the *m*/*z* value (*z* = 1 for all features). Colours represent the fold change of the feature. **e**, A list of measured *m*/*z* values (*z* = 1 for all features, positive ionization) of the chlorinated metabolites and their assigned molecular formulae. **f**, Lipidomics comparison of extracts from ∆*ngl* relative to wild-type *N. punctiforme* ATCC 29133. *P* values were determined by a two-tailed Student’s *t*-test. Purple diamonds represent the parent ion of features of interest. Purple circles represent features originating from alternative ion adducts or source fragment artefacts. Grey circles represent other features. **g**, Extracted ion chromatograms for **5** (*m*/*z* = 555.3658 ± 5 ppm) and **7** (*m*/*z* = 838.6533 ± 5 ppm) from the wild type compared to ∆*ngl* and ∆*nglO*. Each trace represents an independent biological replicate. Source data

We next sought to use the halide depletion workflow to identify previously uncharacterized natural products. To begin, we searched microbial genomes for BGCs that encode homologues of two key cylindrocyclophane biosynthetic enzymes: the halogenase CylC and the alkyl halide-derivatizing enzyme CylK^21,22^. We specifically focused on organisms that do not encode homologues of CylI, the resorcinol-forming enzyme in cylindrocyclophane biosynthesis^22^, as we thought they would probably produce distinct natural products. We found that the genetically tractable model cyanobacterium *N. punctiforme* ATCC 29133 contains a BGC that is distinct from the cylindrocyclophane BGC and encodes CylC and CylK homologues (Fig. 3a). This BGC, also known as *pks3*, was previously identified in a survey of polyketide synthase (PKS)-encoding BGCs^29^. Based on the efforts reported here, we propose the *pks3* BGC be named *ngl* and the encoded proteins be named NglA–NglR (for nostochloroside glycolipid).

To discover candidate products of *ngl*, we applied the halide depletion workflow to *N. punctiforme* ATCC 29133. Analysis of culture extracts by LC–MS initially revealed two chlorinated metabolites that markedly decreased in abundance upon halide depletion (Fig. 3b and Supplementary Table 2). These features include a compound with the molecular formula C_28_H_55_O_8_Cl (**5**) (*m*/*z* calcd. for [M + H]^+^, 555.3658; found, 555.3679) and a desaturated analogue with the molecular formula C_28_H_53_O_8_Cl (**6**). These molecules were less abundant in cultures grown in chloride-depleted media, with relative fold changes of 87.5 (*P* = 5.5 × 10^−8^) and 421.1 (*P* = 6.9 × 10^−6^), respectively, representing the largest fold change and most significant *P* value in the initial metabolomics dataset (Fig. 2b). A second analysis using a lipidomics LC–MS method revealed an additional six depleted metabolites of interest that again had the highest relative fold changes observed (Fig. 3c and Supplementary Table 3), with representative metabolite C_46_H_89_O_9_Cl (**7**) (*m*/*z* calcd. for [M + NH_4_]^+^, 838.6533; found, 838.6566) having a relative fold change of 607.0 (*P* = 1.8 × 10^−2^). Molecular networking suggested these features were related to **5** (Fig. 3d), with five features (**7**–**11**) having masses consistent with fatty-acid ester adducts of **5** (Fig. 3e). In a replicate halide depletion experiment (Supplementary Table 4) analysed by a lipidomics LC–MS method, we also noted a feature (**12**) with a mass consistent with a pseudo-dimer of **5**, and molecular networking clustered it with **5**–**11** (Fig. 3e). We did not observe the pseudo-dimer in all cultures grown with chloride, but we never observed it in the absence of chloride. We therefore hypothesized that **12** might derive from cryptic halogenation. Only one chlorinated feature, consistent with C_31_H_59_O_2_Cl (*m*/*z* calcd. for [M + NH_4_]^+^, 516.4542; found, 516.4543), appeared unrelated to the others by its distinct molecular formula. Overall, our halide depletion workflow was extremely effective at highlighting uncharacterized halogenated natural products in *N. punctiforme* ATCC 29133.

**Extended Data Fig. 1:**
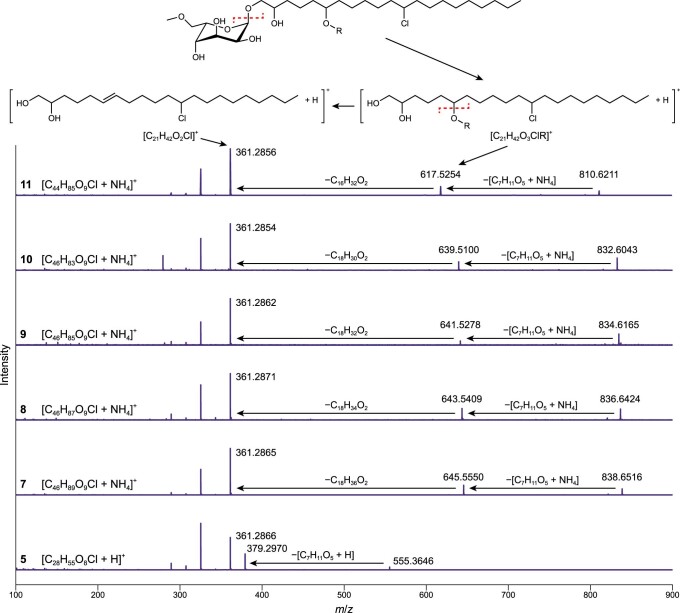
MS/MS spectra of 5 and 7–11. The structure of **5** and **7**–**11**, determined in subsequent experiments (Fig. 4), is shown at the top for reference with the interpretation of key losses shown as dashed lines. Key *m*/*z* values are shown along with the interpretation of neutral losses. The collision energy was 10 V for **5** and 15 V for **7**–**11**. Source data

**Extended Data Fig. 2:**
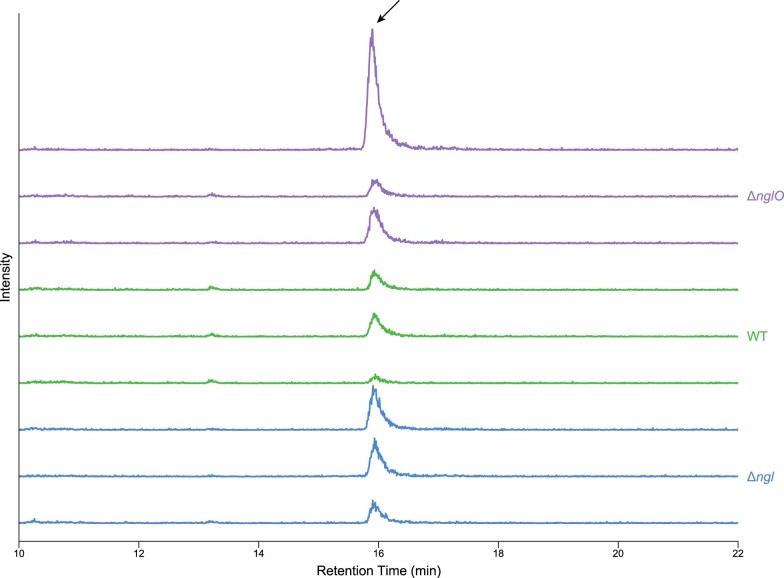
Production of a chlorinated metabolite in *N. punctiforme* ATCC 29133 mutants. Extracted ion chromatograms for [C_31_H_59_O_2_Cl + NH_4_]^+^ (*m*/*z* = 516.4543 ± 5 ppm) from the wild type compared to ∆*ngl* and ∆*nglO*. Each trace represents an independent biological replicate. Data are from cultures that were grown in the absence of fixed nitrogen. The arrow indicates the peak corresponding to [C_31_H_59_O_2_Cl + NH_4_]^+^. Source data

We chose to characterize metabolites **5**–**12** because their molecular network represented the largest of the fold changes observed upon halide depletion. We hypothesized that they originate from *ngl* because their fragmentation patterns show neutral losses of a sugar-like moiety with a lipid-like tail (Extended Data Fig. 1), consistent with the presence of a glycosyltransferase and fatty acyl–AMP ligase (FAAL) in this BGC (Fig. 3a). To confirm this assignment, we created two markerless deletion mutants, one of the *nglA*–*nglO* core biosynthetic genes (hereafter ∆*ngl*) and one of *nglO* (∆*nglO*) encoding the putative alkyl halide-derivatizing enzyme (CylK homologue) (Fig. 3a). LC–MS analysis revealed that **5**–**11** were undetectable in the metabolome of ∆*ngl* (Fig. 3f,g and Supplementary Table 5). In contrast, ∆*nglO* overproduced **5**–**11** (Fig. 3g), consistent with a putative role for NglO in converting **5**–**11** to other, undetected metabolites, although we did not detect the potential product of cryptic halogenation (pseudo-dimer **12**) in these experiments. We detected the unrelated chlorinated feature C_31_H_59_O_2_Cl in both ∆*ngl* and ∆*nglO* (Extended Data Fig. 2), indicating that this compound is not produced by *ngl*. Together, these results demonstrate that *ngl* encodes at least part of the biosynthetic machinery for **5**–**11**.

## Structure of the halogenated metabolites

**Fig. 4:**
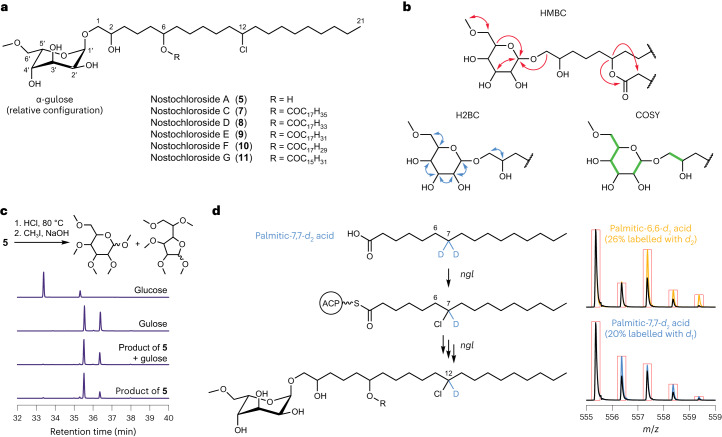
Chlorinated glycolipids from *N. punctiforme* ATCC 29133 contain unusual structural features. **a**, The proposed structures of nostochlorosides A and C–G (**5** and **7**–**11**) based on a detailed characterization of **5** and **7**. Carbons are numbered as referenced in the main text. **b**, Key NMR interactions used to establish bond connectivity. **c**, Identification of the sugar moiety. The scheme shows treatment of **5** to produce a permethylated sugar that was analysed by GC–MS. The traces represent extracted ion chromatography (EIC) results for the C_4_H_8_O_2_^•+^ fragment (*m*/*z* = 88.0519 ± 5 ppm) for the permethylated product derived from **5**, the product derived from **5** co-injected with permethylated gulose, permethylated gulose and permethylated glucose. EICs are normalized to the peak height for their respective *m*/*z* ranges. Each sugar yields multiple peaks that originate from the α- and β-anomers of the pyranose and furanose forms. **d**, Identification of the position of the chlorine substituent. Left: interpretation of deuterium-labelling experiments in *N. punctiforme* ATCC 29133. Right: strain ∆*nglO* was fed palmitic-*d*_2_ acid as indicated. Black traces represent the mass spectrum of unlabelled **5** and coloured traces the experimentally observed mixture of unlabelled and labelled **5**. Red boxes mark the theoretical centroided mass spectrum for a mixture of unlabelled and labelled **5** as indicated. Traces are normalized to M + 0. The increase in intensity at M + 1 with palmitic-7-7-*d*_2_ acid indicates replacement of a deuterium atom with chlorine. Source data

We next aimed to structurally characterize representative products of *ngl*. We used normal-phase silica chromatography and reversed-phase high-performance liquid chromatography (HPLC) with mass-guided fractionation to isolate **5** and **7** from large-scale cultures of ∆*nglO*, which overproduces the metabolites of interest. Both compounds were obtained in low yields (<1 mg) as colourless translucent solids with no detectable UV–vis absorbance. Full NMR characterization (Supplementary Figs. 3–18, Supplementary Tables 6 and 7 and Supplementary Note 1) and additional chemical derivatizations, described in the following, revealed **5** and **7** to be chlorinated glycolipids (Fig. 4a,b). The structural elucidation of **5** and **7** supports the results from molecular networking, identifying **5**–**11** as a family of glycolipids and corresponding fatty-acid ester derivatives that we have named the nostochlorosides.

**Extended Data Fig. 3:**
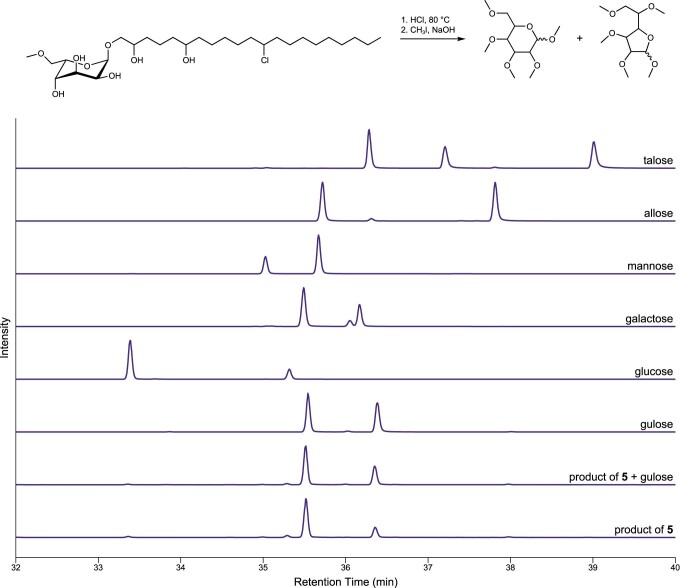
Elucidation of the sugar moiety of 5. The traces represent EICs of the C_4_H_8_O_2_^•+^ fragment (*m*/*z* = 88.0519 ± 5 ppm) for the permethylated product from **5**, the product from **5** co-injected with permethylated gulose, or the permethylated product of the sugar as shown. Data are an expanded version of Fig. 4c showing all the sugars tested. Source data

**Extended Data Fig. 4:**
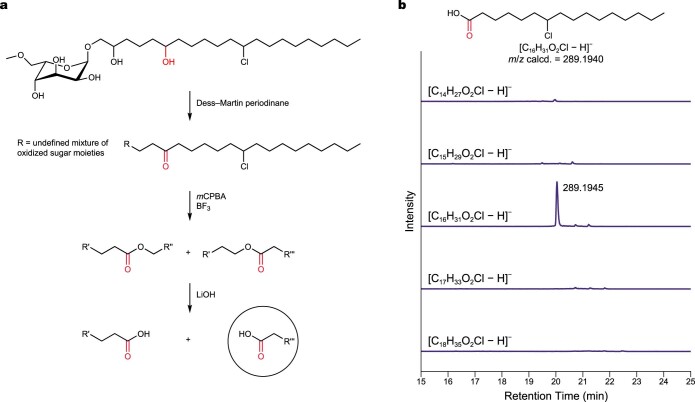
Identification of the location of the side-chain hydroxy group in 5. **a**, Chemical treatment of **5** to produce a chlorinated fatty acid, indicated by the circled product, which was analyzed by LC–MS. **b**, LC–MS analysis of the fatty acid byproducts of **5** in negative ionization mode. The traces represent EICs (±5 ppm) corresponding to the prospective fragments as shown. Peak heights are normalized to the largest peak across all *m*/*z* ranges. The structure of the inferred product is shown at the top. Source data

**Extended Data Fig. 5:**
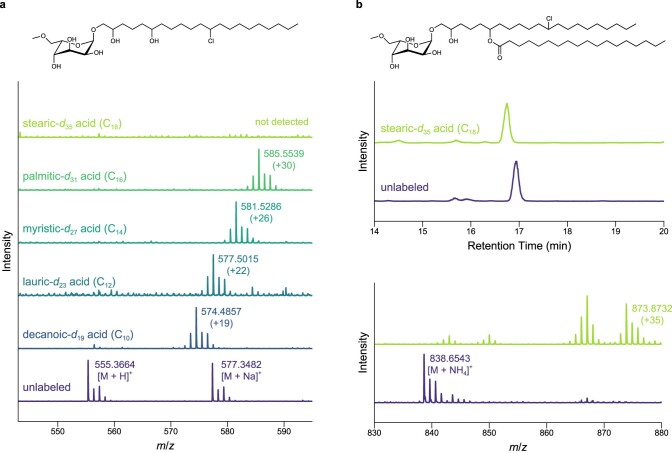
Feeding of isotopically labeled fatty acids to ∆*nglO* and LC–MS analysis of culture extracts. The structure of the compound being analyzed is shown at the top of each panel for reference. Strain ∆*nglO* was grown in the presence of deuterated fatty acids, then analyzed by LC–MS for the incorporation of deuteria into the nostochlorosides. **a**, Analysis of label uptake into **5**. Each trace shows the mass spectrum of **5** labeled with the indicated fatty acid. The spectrum of unlabeled **5** is shown at the bottom for reference. The measured *m*/*z* value for the most intense peak is shown along with the relative nominal mass increase over the unlabeled compound. Spectra are normalized to the largest peak within each trace. Labeled **5** was chromatographically separated from unlabeled **5** and so unlabeled **5** does not appear in the labeled spectra. **b**, Analysis of stearic-*d*_35_ acid uptake into **7**. Strain ∆*nglO* was grown in the presence of stearic-*d*_35_ acid and analyzed for deuteria incorporation into **7**. The top panel shows EICs for unlabeled and labeled **7** (as [M + NH_4_]^+^ ± 5 ppm; *m*/*z* 838.6533 and 873.8730, respectively). The bottom panel shows the mass spectrum at the EIC peak maximum for unlabeled and labeled **7**, demonstrating that all 35 deuteria from stearic-*d*_35_ acid are incorporated into **7**. A peak corresponding to 69 or 70 deuteria was not observed. Source data

To identify the sugar headgroup, we subjected **5** to acid hydrolysis and derivatized the resulting fragments by permethylation. Gas chromatography and mass spectrometry (GC–MS) of the product in comparison to permethylated sugar standards identified the sugar as gulose (Fig. 4c and Extended Data Fig. 3), indicating that the nostochlorosides contain 6-*O*-methylgulose. We also detected traces of glucose in this analysis, possibly arising from trace impurities of other *N. punctiforme* glycolipids in the sample^30^. We were unable to separate permethylated d- and l-gulose by chiral GC, leaving us uncertain as to the absolute stereochemical configuration of this sugar in the nostochlorosides. However, to our knowledge, there are no reports of naturally occurring d-gulose, so we hypothesize that the nostochlorosides contain l-gulose^31–33^. To identify the position of the internal hydroxy group, we used a chemical degradation approach to convert the hydroxy group of **5** into a carboxylic acid fragment (Extended Data Fig. 4a). The fragment we observed indicated the hydroxy group must be located at the 6-position of the intact glycolipid (Extended Data Fig. 4b). To identify the location of the chlorine substituent, we performed stable isotope-feeding experiments with deuterated fatty acids (Supplementary Note 2). We observed the loss of one deuterium atom upon feeding with lauric-*d*_23_ acid (Extended Data Fig. 5a), but not decanoic-*d*_19_ acid, consistent with chlorination at either the 11- or 12-positions of **5** (Fig. 4d). We further observed one deuterium atom loss for palmitic-7,7-*d*_2_ acid, but not palmitic-6,6-*d*_2_ acid, indicating that the nostochlorosides are chlorinated at the 12-position (Fig. 4d). Stearic-*d*_35_ acid was not incorporated into **5**, but it was incorporated into **7** with full deuterium atom retention (Extended Data Fig. 5b), suggesting palmitic acid is the starting unit for nostochloroside biosynthesis. From molecular networking, we propose that **8**–**11** are related to **7** but incorporate fatty acids that vary in length and unsaturation (Fig. 4a).

## NglO oligomerizes 5 via tail-to-tail etherification

We next turned our attention to the putative pseudo-dimer **12**. We hypothesized that this potential product of cryptic halogenation could be generated by the CylK homologue NglO (Npun_F3370) because of CylK’s established role in catalysing monoalkylresorcinol dimerization during cylindrocyclophane biosynthesis^21,22,25^. We were intrigued by the potential origins of **12** because **5**–**11** lack the resorcinol nucleophile used by CylK for alkyl halide substitution. The CylK homologue BrtB was previously shown to catalyse *O*-alkylation of a carboxylic acid with an alkyl chloride to form fatty-acid esters in bartoloside biosynthesis, indicating that CylK homologues can use alternative nucleophiles^34^. However, **5**–**11** also lack a free carboxylic acid, suggesting NglO might use a distinct nucleophile.

**Fig. 5:**
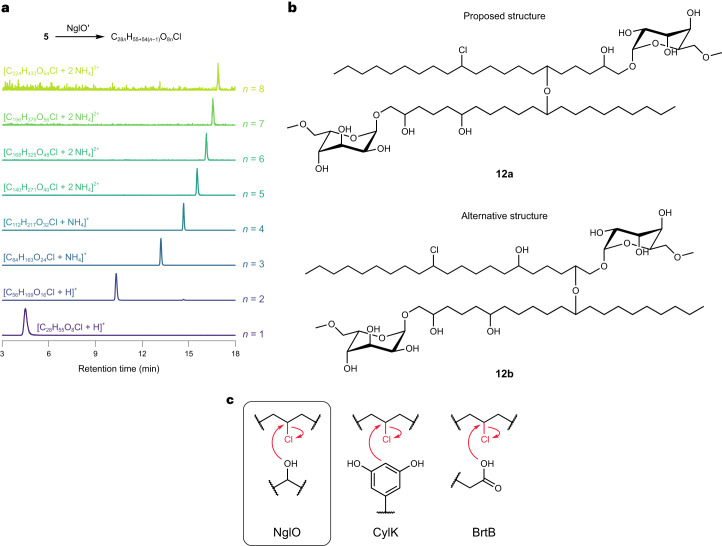
NglO catalyses oligomerization of 5 via an unprecedented etherification reaction. **a**, LC–MS detection of oligomers of **5** in an in vitro assay with purified NglO′. Each trace represents the EIC for the indicated species (±5 ppm). Each EIC is normalized to the maximum intensity within the extracted *m*/*z* window. **b**, Proposed structure (**12a**) for the pseudo-dimer of nostochloroside A (**12**) based on computational analysis and an alternative possible structure (**12b**) that experimental data do not rule out. Carbons are numbered as referenced in the main text relative to each nostochloroside A monomer. **c**, Comparison of alkyl halide derivatization reactions catalysed by CylK homologues known so far. Source data

**Extended Data Fig. 6:**
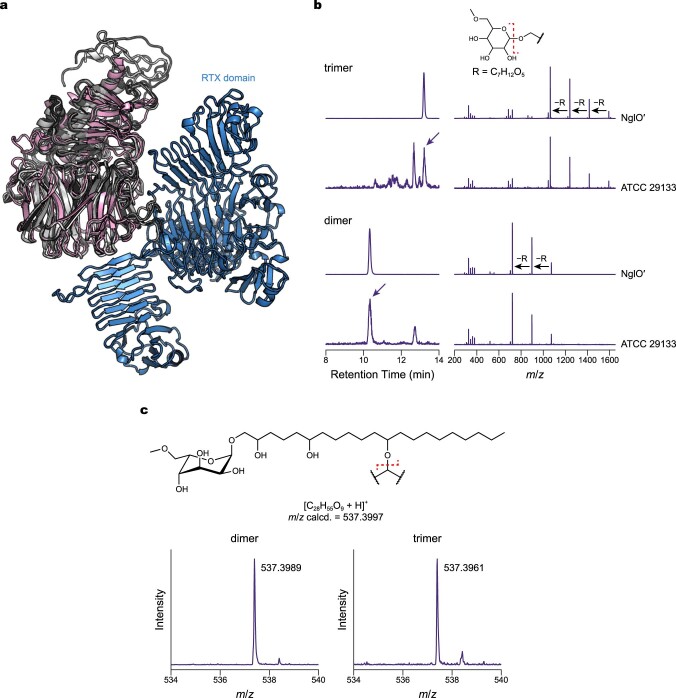
Analysis of NglO. **a**, Homology modeling of NglO. Five AlphaFold predictions (gray and blue) are shown super-imposed onto the solved CylK X-ray crystal structure (PDB ID 7RON, pink). The RTX domain in NglO is shown in blue, corresponding to the region truncated in NglO′. **b**, Comparison of features detected in the in vitro assay with extracts of wild-type ATCC 29133. Traces are scaled to the largest peak. Left, EICs of the dimer and trimer for the in vitro assay and the cell extracts. Arrows indicate the peak analyzed by MS/MS. Right, MS/MS spectra of the dimer and trimer of **5**. The structure shows the observed loss of the sugar moiety. **c**, Close-up view of the MS/MS spectra for the in vitro dimer and trimer showing the peak corresponding to the fragment C_28_H_55_O_9_. The structural interpretation of the fragmentation is shown for reference. Source data

To better understand the biosynthetic role of NglO, we heterologously expressed and purified (Supplementary Fig. 21) a truncated form of the enzyme (NglO′) missing a C-terminal repeats-in-toxin (RTX) domain that is not found in CylK. We used the truncated form because we were unable to purify soluble full-length NglO. Structural prediction by AlphaFold^35^ suggests NglO contains the β-propeller and N-terminal Ca^2+^ domains of CylK, with some architectural differences in the N-terminal domain, but differs primarily by the presence of the RTX domain (Extended Data Fig. 6a), which might play a role in membrane association^36^. Upon incubating purified NglO′ with **5**, we detected masses corresponding to oligomeric products containing up to eight units derived from **5** (Fig. 5a), with the dimeric product mass being consistent with **12**. A control reaction without enzyme did not yield detectable oligomerization. We also did not detect activity in assays with **7** as substrate, and a reaction containing both **5** and **7** produced only oligomers of **5**. All the observed products were chlorinated, and we did not detect any products derived from macrocyclization.

To confirm that the observed NglO′ activity is relevant in vivo, we screened extracts from multiple wild-type *N. punctiforme* cultures and identified one with a comparatively high amount of **12**. This extract contained features with indistinguishable *m*/*z* values, retention times and MS/MS spectra for the pseudo-dimer (**12**) and pseudo-trimer generated in the in vitro NglO′ assay (Extended Data Fig. 6b). These results confirm that NglO generates **12** through the oligomerization of nostochloroside A, and furthermore that NglO catalyses an alkyl halide-utilizing reaction distinct from those performed by CylK and BrtB.

As NglO oligomerizes nostochloroside A into many different chemical species, we could not obtain enough of **12** or a higher oligomerization state for NMR characterization. Using the present culture conditions and isolation methods, we estimate this would require >1,000 l of culture, which is a substantial burden for a slow-growing phototrophic organism. As an alternative approach to predicting the structures of the oligomers, we turned to MS data and computational analyses of NglO. The MS/MS fragmentation patterns of the pseudo-dimer (**12**) and pseudo-trimer show successive losses of the sugar moiety (Extended Data Fig. 6b), indicating that the repeating units are not connected at the sugar. The MS/MS spectra are consistent with replacement of the chlorine with a hydroxy group (Extended Data Fig. 6c). Given the lack of other discernable nucleophiles in **5**, we interpret this fragment to indicate that the pseudo-dimer **12** contains an ether linkage between either O2 and C12 or O6 and C12. We detected only a single peak for each oligomeric state in our LC–MS analysis, indicating that NglO produces just one of these two possible ether linkages.

**Extended Data Fig. 7:**
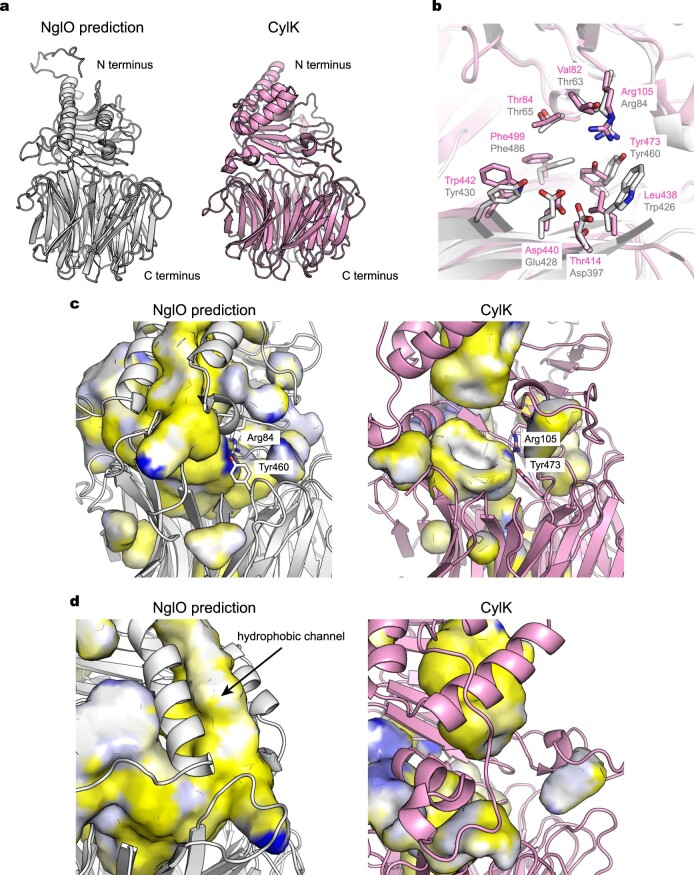
Analysis of the NglO structural prediction. **a**, Comparison of the AlphaFold prediction for NglO (white) with the X-ray crystal structure of CylK (pink, PDB ID 7ROO). For clarity, the C-terminal RTX domain in NglO is omitted. **b**, Comparison of residues in the active site between the AlphaFold prediction of NglO (white) and CylK (pink). **c**, Visualization by parKVfinder of the substrate binding cavity in NglO (left, white) and CylK (right, pink). Yellow corresponds to hydrophobic regions and blue corresponds to hydrophilic regions. **d**, Alternate view of the N-terminus of the predicted NglO structure (left, white) and the CylK X-ray crystal structure (right, pink) highlighting the predicted architectural differences in the N-terminal domain.

To gain insights into how NglO may interact with its substrate, we further compared the AlphaFold structure prediction for NglO to the solved X-ray crystal structures of CylK. The NglO prediction showed good agreement with the CylK crystal structures (Extended Data Fig. 7a), including near superposition of the conserved alkyl halide-binding residues Arg84 and Tyr460 (Arg105 and Tyr473 in CylK) and the nucleophile-activating base Glu428 (Asp440 in CylK) (Extended Data Fig. 7b), indicating that the AlphaFold prediction accurately recapitulates important structural features of this enzyme family. Conservation of the key halide-binding and nucleophile-activating residues suggests that members of the CylK family retain similar mechanisms. Visualization of the predicted NglO binding pocket using parKVFinder^37,38^ revealed a larger and more hydrophobic cavity than in CylK (Extended Data Fig. 7c), which is consistent with NglO acting on larger substrates. Intriguingly, this visualization also revealed a distinct N-terminal domain structure containing a large hydrophobic groove that is absent in CylK (Extended Data Fig. 7d), which might represent a lipid-binding cavity. Much like CylK, the predicted NglO active site and substrate binding pocket are distinctly hydrophobic (Extended Data Fig. 7c). If the NglO structural prediction is correct, we would not expect NglO to accommodate a polar gulose moiety near the active site as would be required if O2 were the nucleophile for ether bond formation. We therefore favour the hypothesis that NglO uses the O6 of nostochloroside A to yield a structure for **12** with an ether linkage between O6 and C12 of two nostochloroside A subunits (Fig. 5b). Also supporting the involvement of an O6 nucleophile, we note that NglO′ did not oligomerize nostochloroside C (**7**), which is already modified at the O6 position. Nonetheless, a definitive assignment for **12** will require additional biochemical analyses of NglO using synthetic substrates and/or a large-scale (~1,000 l) isolation and NMR characterization.

Overall, these data show that NglO catalyses an unprecedented enzymatic etherification reaction using a hydroxy group as a nucleophile to displace an alkyl halide electrophile. The pathway encoded by *ngl* represents a new example of cryptic halogenation, further expanding the reactivity of the CylK family of alkyl halide-utilizing enzymes to encompass hydroxy, resorcinol and carboxylate nucleophiles (Fig. 5c).

## Halide depletion is a function-agnostic discovery tool

**Extended Data Fig. 8:**
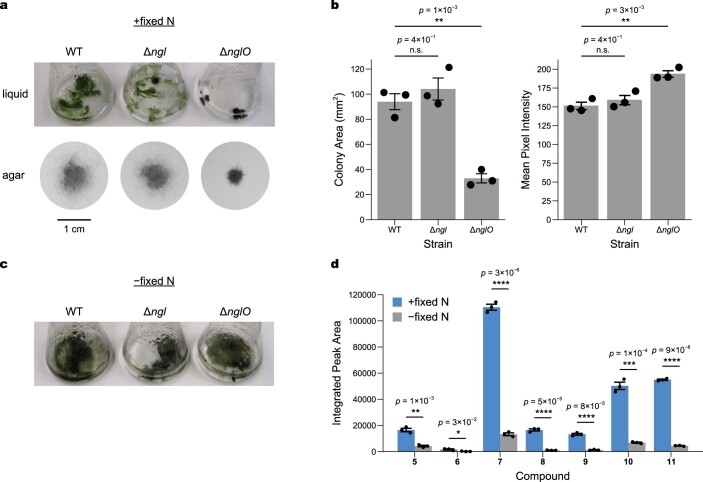
Phenotype of strain ∆*nglO* and the influence of nitrogen fixation. For statistical analyses, error bars represent the standard error of three independent biological replicates. Significance markers represent the *p*-value from a two-tailed Student’s t-test: n.s., not significant (*p* > 0.05); *, *p* ≤ 0.05; **, *p* ≤ 0.01; ***, *p* ≤ 0.001; ****, *p* ≤ 0.0001. Black points represent individual measurements and bars represent the mean. **a**, Representative cultures of *N. punctiforme* strains growing in liquid BG-11 medium (top) or on soft agar AA/4 medium (bottom). **b**, Quantitation of soft agar growth by colony area (left) or pixel intensity (right). Pixel intensity represents an indirect measurement of cell density. **c**, Representative cultures of *N. punctiforme* strains growing in liquid BG-11 medium without a fixed nitrogen source. **d**, LC–MS quantitation of glycolipids **5**–**11** from wild-type ATCC 29133 grown in liquid BG-11 with or without fixed nitrogen (nitrate). Injection volumes were normalized by chlorophyll absorbance of the sample measured at 665 nm. Source data

We next wondered whether the nostochlorosides play physiological roles for the producing cyanobacterium that might be missed by traditional bioactivity or functional screens. We did not observe a growth defect for ∆*ngl* during routine laboratory culturing, but ∆*nglO* showed more aggregation in liquid culture (Extended Data Fig. 8a) and lower motility on soft agar (Extended Data Fig. 8a,b), with a similar or greater growth rate compared to the wild type (Extended Data Fig. 8b). We did not observe a growth defect in media free of fixed nitrogen, and the aggregation phenotype of ∆*nglO* was less pronounced under nitrogen fixation conditions (Extended Data Fig. 8c). Surprisingly, we measured significantly less **5**–**11** in wild-type cultures undergoing nitrogen fixation (Extended Data Fig. 8d), despite the reported upregulation of *ngl* under this condition^39,40^. Although the biological functions of these glycolipids remain unknown, these observations show that **5**–**11** are not required for nitrogen fixation.

**Extended Data Fig. 9:**
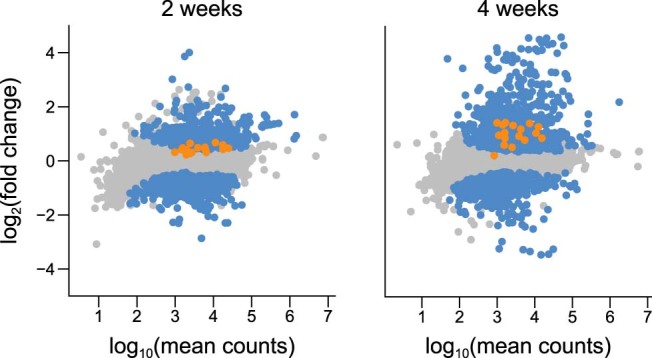
Halide depletion induces a transcriptional response in *N. punctiforme* ATCC 29133. Comparison of transcriptional responses after 2 weeks (left) and 4 weeks (right). Fold changes represent feature abundance in cultures grown without chloride relative to those grown with chloride. *p*-values were calculated with DESeq2 using the Wald test and corrected for multiple testing using the Benjamini–Hochberg method. Each data point represents the mean of three independent biological replicates per condition. Statistically significant changes (*p* ≤ 0.001) are shown in blue. Features in *ngl* are shown in orange. All other features are shown in gray.

Finally, to examine the effect of halide depletion on cyanobacterial physiology and global gene expression, we performed RNA-sequencing of two- and four-week-old *N. punctiforme* cultures grown in the presence and absence of chloride (Extended Data Fig. 9 and Supplementary Tables 8 and 9). The largest differences observed in the absence of chloride were enzymes involved in precursors to the UV-protective sunscreen molecule shinorine^41,42^. Halide-free cultures also overexpressed other general stress-response proteins, including a high light-inducible protein, catalases and accessory photosystem proteins. Notably, halide-depleted cultures showed two- to threefold higher expression of *ngl* by four weeks (Extended Data Fig. 9), despite producing up to 600-fold less of the nostochlorosides (Fig. 3e). This large disparity between gene expression and biosynthetic output indicates that halide bioavailability, and not a transcriptional response, limits the production of nostochlorosides during halide depletion. These findings demonstrate how halide depletion can reveal new structural motifs derived from cryptic halogenation, independently of activity-guided, functional or transcriptional screening approaches.

## Discussion

Traditionally, natural product discovery has been guided by bioactivity assays or other functional screens. More recently, strategies such as elicitor screening, promoter engineering, heterologous expression and correlating genome content with metabolite production have been employed to access the products of cryptic BGCs^5,6,43–45^. However, it is often challenging and laborious to identify specific metabolites of interest from complex metabolomes. Here we report that manipulating substrate availability for a key biosynthetic enzyme provides a complementary approach to target the discovery of natural products derived from enzymatic halogenation (Fig. 1).

To demonstrate its utility, we applied our halide depletion workflow to identify additional paracyclophane derivatives in *C. licheniforme* ATCC 29412 (Fig. 2) and to discover the natural products made by *ngl* (*pks3*) in *N. punctiforme* ATCC 29133 (Figs. 3–5), whose genome was first published in 2001^46^. Notably, *ngl* is one of the highest constitutively expressed BGCs in *N. punctiforme* ATCC 29133, and it had previously been suggested that its orphan status was due to challenges in detecting the corresponding natural product(s)^47^. We found that LC–MS and halide depletion readily overcame these detection challenges and pinpointed the *ngl*-derived products **5**–**11** (Fig. 3), demonstrating how this strategy offers a convenient and complementary approach for halogenated natural product discovery efforts. As halides are not generally required for bacterial growth^48^, we anticipate that this method can be applied to many other microorganisms that grow in defined low-salt minimal media. In particular, it may prove useful for identifying other products of cryptic halogenation involving CylK homologues, as there are many additional instances of CylK-encoding genes colocalized with CylC-encoding genes in BGCs^49^.

Our halide depletion workflow offers several major advantages over existing discovery approaches, including the use of simple growth conditions without the need for extensive screening or time-consuming genetic manipulations. The discovery of the nostochlorosides highlights these advantages. First, although *N. punctiforme* ATCC 29133 is genetically tractable, its slow growth rate made obtaining the ∆*ngl* and ∆*nglO* mutants laborious. Applying halide depletion allowed us to begin our isolation and characterization efforts in parallel with genetic studies. Second, the size of the *ngl* BGC (~36 kb) means it would be challenging to express all the necessary biosynthetic enzymes in a heterologous host. We also note that the *ngl* BGC does not appear to encode the acyltransferase for forming nostochlorosides C–G (**7**–**11**). We hypothesize that this acyltransferase is encoded elsewhere in the genome, making the full family of nostochlorosides challenging to discover outside of the native producer. Third, this approach enabled us to discover the nostochlorosides and their intriguing chemical features and enzymology without relying on their bioactivity. We note that halide depletion may be unsuitable for marine organisms that may require halides for growth. Moreover, the physiological effects of halide depletion are understudied compared to high salt tolerance, and so it is unknown if halide depletion causes non-specific metabolic effects in other microbial phyla. Nonetheless, this work demonstrates that manipulating access to an essential biosynthetic precursor is a powerful approach for natural product discovery.

Given that we targeted the nostochlorosides for discovery due to halogenation in their biosynthesis, their other unusual structural features were unexpected. At first glance, the nostochlorosides superficially resemble the heterocyst glycolipids produced by *N. punctiforme* and other cyanobacteria^30^. These glycolipids typically carry a glucose headgroup ether-linked to a long-chain fatty alcohol (C_26_ or C_28_)^30^ and protect the nitrogen-fixing heterocysts from oxygen, which can interfere with nitrogen fixation^50^. Although ether lipids are well-described in biology, for example in heterocyst glycolipids^30^, archaeal lipids^51^ and plasmalogens^52^, the specific linkage to a vicinal diol in the nostochlorosides is comparatively rare. In addition, whereas the heterocyst glycolipids typically contain glucose as their polar headgroup^30^, nostochlorosides contain the rare sugar gulose (Fig. 4c). Gulose has been reported in only a handful of natural products, including an archaeal membrane lipid^31^, extracellular and cell wall components of algae^31,32^ and, perhaps most notably, the antibiotic bleomycin^33^. In bleomycin biosynthesis, l-gulose is thought to originate from the enzyme BlmG acting as a d-mannose-5-epimerase^53^. The sugar epimerase in *ngl*, NglK (Npun_F3566), bears little similarity to BlmG (10.3% identity) or other characterized epimerases, and so the immediate precursor to gulose in the nostochlorosides is unknown. The unexpected discovery of gulose in the nostochlorosides suggests that gulose and/or other rare sugars might have underappreciated roles in biology.

Another notable structural feature of the nostochlorosides is their C_21_ lipid tail. Odd-chain-length lipids are uncommon in biology because lipids are typically built from sequential additions of two carbon units. Indeed, our stable isotope-feeding studies (Fig. 4d) suggested that palmitic acid (C_16_) is the starter unit for biosynthesis of **5**. Nostochloroside biosynthesis therefore requires extension of palmitic acid by a total of five carbon units, a pathway with little precedence. Based on the enzymes encoded by *ngl*, we hypothesize that the unusual nostochloroside tail originates from cleavage of a larger PKS-derived product (Supplementary Fig. 22 and Supplementary Note 3). This hypothesis is in contrast to other mechanisms for producing odd-chain-length lipids, such as decarboxylation as suggested for barbamide biosynthesis^54^ or carbon excision in microcystin biosynthesis^55^. Testing this hypothesis will require in vitro biochemical characterization of the *ngl* PKS assembly line, but it is clear from the nostochloroside structures that *ngl* employs uncharacterized biosynthetic transformations.

We were also surprised by the structures of the nostochloroside oligomers. Although we predicted that NglO would use the alkyl chloride **5** as a substrate, we unexpectedly found that it catalyses polymerization instead of dimerization. The formation of higher oligomerization states in vitro (Fig. 5a) might partially explain our inability to consistently detect **12** in our experiments, for example, if the larger products extract inefficiently or are difficult to ionize in MS. Polymerization of **5** by NglO involves etherification (Fig. 5) to create branched lipids that are distinct from previously described fatty-acid esters of hydroxylated fatty acids^56^, but which bear some resemblance to the nigricanosides, a family of metabolites from the eukaryotic green alga *Avrainvillea nigricans*^57^. Etherification has not been previously observed for CylK homologues and further indicates that activation of alkyl chloride electrophiles is a key feature of this enzyme family.

Natural product discovery remains an ongoing challenge. Untargeted metabolomics frequently uncovers a wealth of molecular features that represent everything from artefacts to true metabolites^58^, so there is a continuing need to streamline the identification of molecules of interest. The discovery of the *ngl*-derived nostochlorosides by halide depletion demonstrates how alternative approaches, informed by an understanding of biosynthetic logic, can accelerate the process of natural product discovery. Although our approach is limited to a subset of natural products, namely halogenated natural products and natural products derived from cryptic halogenation, it is agnostic towards metabolite function or bioactivity. It may also greatly simplify the process of connecting metabolites to their corresponding BGCs by focusing on halogenase-encoding pathways. We envision generalizing this approach by manipulating the availability of other biosynthetic building blocks, including metal ions, amino acids and sugars. This general approach could also be coupled to traditional activity assays to rapidly identify bioactive compounds derived from specific precursors. Given the growing appreciation for cryptic halogenation in natural product biosynthesis, we anticipate that halide depletion and other biosynthesis-guided MS-based approaches will continue to uncover new families of natural products.

## Methods

### General materials and methods

Pure water (18.2-MΩ resistivity) was provided by a MilliQ water purification system and used for the bacterial media and LC–MS. MS-grade organic solvents were from Burdick & Jackson (Honeywell). MS-grade formic acid was from Sigma-Aldrich or Pierce (Thermo Scientific). Flash silica chromatography was performed using SiliaFlash P60 gel (SiliCycle). Unless otherwise stated, all other reagents were from Sigma-Aldrich. Preparative thin layer chromatography (TLC) was performed using 20 × 20-cm Uniplates with GF silica and a thickness of 1,000 µm (Analtech). For preparative TLC, the product was visualized by staining a small slice of the TLC plate with KMnO_4_. High-resolution MS data were collected on an Agilent 6530 Q-TOF system.

### Bacterial cultivation

For routine cultivation, cyanobacteria were grown in BG-11 medium containing 1.5 g l^−1^ NaNO_3_, 0.04 g l^−1^ K_2_HPO_4_, 0.02 g l^−1^ Na_2_CO_3_, 0.001 g l^−1^ Na_2_EDTA, 0.006 g l^−1^ citric acid, 0.006 g l^−1^ ferric ammonium citrate, 7.5 g l^−1^ MgSO_4_·7H_2_O, and 0.0537 g l^−1^ CaCl_2_·6H_2_O. A concentrated stock solution of trace elements containing 2.86 g l^−1^ H_3_BO_3_, 1.81 g l^−1^ MnCl_2_·7H_2_O, 0.22 g l^−1^ ZnSO_4_·7H_2_O, 0.39 g l^−1^ Na_2_MoO_4_·2H_2_O, 0.11 g l^−1^ CuSO_4_·5H_2_O and 0.0477 g l^−1^ CoSO_4_·7H_2_O was diluted 1,000-fold into the BG-11, then 25 ml of supplemented BG-11 was aliquoted into 250-ml Erlenmeyer flasks and sterilized by autoclaving. Fixed nitrogen-free cultures were grown by omitting NaNO_3_ from the medium. Cyanobacteria were incubated at room temperature (22–23 °C) at 50 r.p.m. on an orbital shaker (5.08-cm orbit diameter). Illumination was provided using full-spectrum white light-emitting diodes (LEDs) with a colour temperature of 5,000 K (Nichia part no. NFSW757GT-Rsp0a, mounted on a LUXdrive I033 DUO linear LED array, part no. I033-N75H50-SO from LEDsupply). The *C. licheniforme* was grown using a light intensity of 15 µmol m^−2^ s^−1^, and *N. punctiforme* was grown using an intensity of 20 µmol m^−2^ s^−1^.

*Escherichia coli* was grown in lysogeny broth (LB) containing 10 g l^−1^ tryptone, 5 g l^−1^ yeast extract and 5 g l^−1^ NaCl. Cultures were incubated in 5 ml of medium in 15-ml round-bottomed culture tubes (Falcon) while shaking at 180 r.p.m. (2.54-cm orbital diameter) at 37 °C. Solid media contained 15 g l^−1^ agar.

### Halide depletion and metabolite extraction

Halide-free BG-11 was prepared as above, except that 0.0334 g l^−1^ CaSO_4_·2H_2_O was substituted for CaCl_2_·6H_2_O in the medium, 1.38 g l^−1^ MnSO_4_·H_2_O was substituted for MnCl_2_·4H_2_O in the trace elements solution, and the medium was sterilized by filtration instead of autoclaving. For halide-depletion experiments, 10 ml of a starter culture grown in halide-replete medium was centrifuged at 3,220*g* for 2 min. The supernatant was decanted, and the cells were resuspended in 10 ml of halide-free BG-11. This process was repeated three more times. Fresh medium (25 ml in 125-ml flasks) of halide-replete or halide-free BG-11 was inoculated with 1 ml of the resuspended cells. Comparison of the different *N. punctiforme* strains was performed similarly using halide-replete medium. Cyanobacteria were collected by transferring the culture to a 50-ml conical tube, centrifuging for 10 min at 3,220*g*, and decanting the supernatant. The pellet was resuspended in 1 ml of water and transferred to a 1-dram vial. The vial was frozen at −70 °C for at least 4 h and the contents were lyophilized overnight.

For *C. licheniforme* metabolite analysis, cultures were collected after 45 days, and the metabolites were extracted by adding 1 ml of 5:2 ethanol/water and 1 ml of 5:2 *n*-heptane/ethyl acetate to the lyophilized material. The mixture was sonicated in a water bath sonicator for 20 min and then stirred at 1,000 r.p.m. for 1 h. The vials were centrifuged for 5 min at 200*g* to facilitate phase separation. The top (deep green) and bottom (yellow) layers were centrifuged in 1.7-ml microcentrifuge tubes for 15 min at 16,200*g*. The bottom layer was centrifuged a second time for 15 min at 16,200*g* and the supernatant was transferred to an HPLC vial for LC–MS analysis.

For *N. punctiforme* metabolite analysis, cultures were collected after 14 and 28 days, and the metabolites were extracted by adding 2 ml of 2:1 chloroform/methanol to the lyophilized material. The mixture was sonicated in a water bath sonicator for 5 min and then stirred at 1,000 r.p.m. for 1 h. The slurry (1.4 ml) was transferred to a 1.7-ml microcentrifuge tube and centrifuged for 15 min at 16,200*g*. The supernatant (1 ml) was transferred to a clean tube, dried in a vacuum concentrator (CentriVap), redissolved in 0.5 ml of methanol, and centrifuged again for 10 min at 16,200*g*. The supernatant was then transferred to an HPLC vial for LC–MS analysis.

### LC–MS analysis and molecular networking

LC–MS and LC–MS/MS were performed on an Agilent 1260 Infinity LC system connected to an Agilent 6530 Q-TOF system operating in the standard *m*/*z* range (100–3,200). A calibration solution (Agilent G1969-85001) was infused at 4 µl min^−1^ with reference masses set to 121.0509 and 922.0098 for the positive ionization mode. The MS parameters were as follows: gas temperature (N_2_) of 300 °C; drying gas, 8 l min^−1^; nebulizer, 35 psig; fragmentor, 175 V; skimmer, 65 V; V_Cap_, 3,500 V; OCT 1 RF V_pp_, 750 V; scan time, 500 ms. Automatic MS/MS was performed in a separate run using collision-induced dissociation with N_2_, a narrow (~1.3 a.m.u.) isolation width, and collision energy (in volts) determined by the parent ion *m*/*z* according to the formula 10 + 0.02 × (*m*/*z*). Chromatographic separations were performed on a Hypersil GOLD aQ C18 column (Thermo Scientific) with dimensions of 3 × 100 mm and a particle size of 3 µm. The column temperature was 30 °C and the flow rate was 0.5 ml min^−1^. For general analysis, mobile phase A was water with 0.1% formic acid and mobile phase B was acetonitrile with 0.1% formic acid. The general separation consisted of 1% B for 0–2 min, a linear gradient from 1% to 99% B over 2–18 min, 99% B over 18–23 min, a linear gradient from 99% to 1% B over 23–24 min, and a re-equilibration at 1% B over 24–30 min. For lipidomics, mobile phase A was 40% water/60% acetonitrile with 10 mM ammonium formate and 0.1% formic acid, and mobile phase B was 90% isopropanol/10% acetonitrile. The lipidomics separation consisted of 40% B for 0–2 min, a linear gradient from 40% to 99% B over 2–20 min, 99% B over 20–23 min, a linear gradient from 99% to 40% B over 23–24 min, and a re-equilibration at 40% B over 24–30 min. For enzyme or isolated compound analysis, or experiments where normalization was not necessary, the injection volume was 5 µl. For cyanobacteria culture or strain comparisons, samples were measured for chlorophyll absorbance at 665 nm and normalized to a maximum injection volume of 10 or 15 µl.

For comparative metabolomics, LC–MS data were exported from MassHunter Qualitative Analysis vB.07.00 (Agilent) to the mzData format and analysed with MZmine^59^ v2.53. Chromatograms were constructed using the ADAP Chromatogram Builder^60^, deconvoluted using the Wavelets (ADAP) algorithm, isotopically grouped, joined and gap-filled. Where applicable, an *m*/*z* tolerance of 0.01 or 20 ppm was applied. For statistical tests and calculating fold changes, gap-filled features with counts less than the noise value of 100 were treated as having a pseudocount of 100, and *P* values were determined by a two-tailed Student’s *t*-test from three biological replicates for each condition. Data were plotted using the matplotlib^61^ v3.3.2 and seaborn^62^ v0.11.0 packages for Python. Fragmentation data for molecular networking was collected in a separate LC–MS/MS run using precursor ion selection prioritized by the differentially abundant features. Molecular networks were generated using MetGem^63,64^ v1.3.6 using default parameters: *m*/*z* tolerance of 0.02, minimum matched peaks of 4, keeping peaks outside of the ±17-Th window, and keeping each peak in the top 6 in the ±50 window. The molecular networks were visualized using Cytoscape v3.7.2. Nodes of interest were manually annotated with fold changes from the comparative metabolomics. Theoretical mass spectra were simulated using MassHunter Qualitative Analysis vB.07.00, and stable isotope incorporation was estimated using the curve_fit function from the SciPy v1.5.2 Python package to perform a nonlinear least-squares fit of theoretical labelled and unlabelled spectra to the observed spectrum.

### *N. punctiforme* strain construction

Construction of strains ∆*ngl* and ∆*nglO* was performed as previously described^40^, except that the shuttle vectors were constructed using Gibson assembly^65^. To create the shuttle vectors, plasmid pRL278 (ref. ^66^) was linearized by polymerase chain reaction (PCR) using primers CTGTCAGACCAAGTTTACTC and AACTCCAGCATGAGATCC. The product was digested with DpnI (New England Biolabs) for 1 h and purified using a QIAquick PCR Purification Kit (Qiagen). Regions of length ~1 kb upstream and downstream of the target locus were amplified from *N. punctiforme* ATCC 29133 genomic DNA. For ∆*ngl*, the upstream region was amplified using primers ATCTCATGCTGGAGTTCTTCAGACTCAAAGCAAATCAAAGC and CACTTTCACTGAAGTAAAAGTTAGCTATTTATAGACACCTTATTTTAAATAACTACGC, and the downstream region was amplified using primers GCGTAGTTATTTAAAATAAGGTGTCTATAAATAGCTAACTTTTACTTCAGTGAAAGTG and GAGTAAACTTGGTCTGACAGCTCTTGTGCCTCTTTTAAGG. For ∆*nglO*, the upstream region was amplified using primers ATCTCATGCTGGAGTTCTTCATGATTGAGCATGGCTTCTC and CACTTTCACTGAAGTAAAAGTTAGCTATGTTTTTTATGACTTTAATACGAACACTG, and the downstream region was amplified using primers CAGTGTTCGTATTAAAGTCATAAAAAACATAGCTAACTTTTACTTCAGTGAAAGTG and GAGTAAACTTGGTCTGACAGCTCTTGTGCCTCTTTTAAGG. The fragments were purified using a QIAquick PCR Purification Kit (Qiagen), and the linearized pRL278 was assembled with the upstream and downstream fragments using NEBuilder HiFi (New England Biolabs) to create plasmids pRL278-∆*ngl* and pRL278-∆*nglO*. The reaction products were transformed into *E. coli* Top10 and selected on LB agar with 50 µg ml^−1^ kanamycin. Single colonies were grown in LB with 50 µg ml^−1^ kanamycin, and plasmids were isolated using an E.Z.N.A. Plasmid DNA Mini Kit (Omega Bio-tek). The shuttle vectors were transformed into *E. coli* strain UC585 (ref. ^67^) by electroporation and selected on LB agar containing 17 µg ml^−1^ chloramphenicol, 50 µg ml^−1^ carbenicillin and 50 µg ml^−1^ kanamycin. Biparental mating between UC585/pRL278-∆*ngl* or UC585/pRL278-∆*nglO* with *N. punctiforme* ATCC 29133 and selection for double-crossovers was performed as previously described^40^, except that BG-11 was used instead of AA/4. Genomic deletions of *ngl* and *nglO* were confirmed by colony PCR using OneTaq (New England Biolabs). Genomic DNA isolated from the mutants (using a Qiagen DNeasy Ultraclean Microbial kit) did not yield PCR product using primers TTTGCTCTACTAGTGACAGG and AGCAGCACAGGAAATTCAC that bind to *nglO*. Deletions were further confirmed by amplification with primers TGGTGAAAAACTGGAATGCC and GTAATTGTAGCCTTTTGCGC for ∆*ngl*, and TACGCCAAATACCCTTTTGC and GTAATTGTAGCCTTTTGCGC for ∆*nglO*, followed by Sanger sequencing of the product.

### RNA-sequencing

RNA-sequencing was performed on the same *N. punctiforme* ATCC 29133 cultures as for halide depletion. The cultures were quickly homogenized by pipetting, and 1 ml was transferred to a 1.7-ml microcentrifuge tube. The tube was centrifuged for 30 s at 16,200*g*. The supernatant was carefully aspirated, and the pellet was immediately frozen in liquid nitrogen. Culture harvesting was staggered so that the total time between homogenization and snap-freezing was less than 2 min. After harvesting, the remaining culture was processed for metabolomics. Samples for RNA-seq were stored at −70 °C until submission to the standard RNA-seq service provided by Genewiz (Azenta Life Sciences). Briefly, RNA isolation was performed using an RNeasy Plus Mini Kit (Qiagen). Ribosomal RNA was depleted using a QIAseq FastSelect kit (Qiagen), and libraries were prepared using a NEBNext Ultra II RNA Library Prep Kit (New England Biolabs). Sequencing was performed on an Illumina HiSeq system with 2 × 150-bp configuration, single index and data were provided in the fastq format. Reads were aligned using Bowtie 2 (ref. ^68^), and gene expression was counted using featureCounts^69^. Differential gene expression was analysed using DESeq2 (ref. ^70^). Data were deposited in the Sequence Read Archive (http://www.ncbi.nlm.nih.gov/sra) under accession no. PRJNA868493.

### Motility assays

*N. punctiforme* motility assays were performed as previously described^71^.

### Nostochloroside purification

The ∆*nglO* mutant was grown in 10- and 20-l carboys sparged with air under a panel of full-spectrum white LEDs (~30 µmole m^−2^ s^−1^). After 28 days, the carboys were harvested by filtration through Whatman 1 paper. The cell paste was collected in 50-ml conical tubes, centrifuged for 10 min at 3,220*g*, decanted and stored at −70 °C until extraction. In a representative purification, 30 ml of cell paste was thawed and transferred to a 1-l bottle. The cells were extracted with 375 ml of 2:1 chloroform/methanol by stirring for 1 h. The slurry was filtered through Whatman 1 filter paper. The solid paste was re-extracted once more with 375 ml of 2:1 chloroform/methanol, filtered, and the combined filtrates were dried on a rotary evaporator.

For purification of **5**, but not **7**, the crude material was saponified by dissolving in 30 ml of methanol with 2 M KOH. The solution was stirred for 4 h at room temperature and then diluted with 270 ml of water. The mixture was acidified with 6 M HCl and then extracted four times with 50 ml of chloroform, and the combined chloroform layers were dried on a rotary evaporator for downstream purification. These steps were not necessary for purification of **5**, but they improved the yield by up to twofold.

The dried material (~0.4 g) was redissolved in a minimal volume of 2% methanol in chloroform and subjected to flash chromatography using a puriFlash 5.250 system (Interchim). Separation was performed on an 80-g RediSep silica cartridge (Teledyne ISCO). Mobile phase A was chloroform and mobile phase B was methanol. The flow rate was 34 ml min^−1^ and the fraction size was 25 ml. The column was equilibrated with eight column volumes (CVs) of 3% B before loading the sample. Compounds **5** and **7** were eluted using a gradient of 3% B for 0–5 CVs, a linear gradient from 3% to 25% B over 5–15 CVs, and holding 25% B over 15–17 CVs. Fractions were screened for **5** and **7** using a modified rapid lipidomics method on the LC–MS system. Samples were diluted tenfold into methanol, and the injection volume was 5 µl. For the rapid method, the column was a Hypersil GOLD aQ C18 column (Thermo Scientific) with dimensions of 3 × 50 mm and 3-µm particle size, and the flow rate was 0.7 ml min^−1^. The rapid gradient was 30% B for 0–0.75 min, a linear gradient from 30% to 99% B over 0.75–3 min, 99% for 3–3.5 min, a linear gradient from 99% to 30% B over 3.5–3.8 min, and re-equilibration at 30% B over 3.8–6 min. Fractions containing **5** or **7**, identified by *m*/*z* values 555.3658 and 838.6533, respectively, were pooled and dried on a rotary evaporator.

Compounds **5** and **7** were further purified by preparative HPLC using a Dionex UltiMate 3000 semi-preparative system. The column was a Hypersil GOLD aQ C18 column (Thermo Scientific) with column dimensions of 10 × 250 mm and a particle size of 5 µm. For **5**, mobile phase A was water with 0.1% formic acid, and mobile phase B was acetonitrile with 0.1% formic acid. The fraction size was 1 ml. The gradient consisted of 60% B for 0–2.78 min, a linear gradient from 60% to 99% B over 2.78–25 min, and 99% B for 25–30 min. For **7**, mobile phase A was 40% water/60% acetonitrile with 10 mM ammonium formate and 0.1% formic acid and mobile phase B was 90% isopropanol/10% acetonitrile. The fraction size was 2 ml. The gradient consisted of 50% B for 0–3.7 min, a linear gradient from 50% to 80% B over 3.7–37 min, and 80% B for 37–43 min. Fractions were screened for **5** and **7** using the rapid lipidomics method, then pooled and dried in a vacuum concentrator (CentriVap) to afford pure **7** and impure **5**. Compound **5** co-eluted with an unknown orange contaminant (possibly a carotenoid) and was further purified by preparative TLC developed with 76.5% chloroform/13.5% methanol/10% acetic acid. Compound **5** was visualized by cutting off a small strip and staining with cerium molybdate. Unstained silica was extracted using methanol and dried on a rotary evaporator. The material was redissolved with 1 ml of methanol, diluted with 1 ml of water, and applied to a 1 g C18 Sep-Pak column (Waters). Pure **5** was eluted with 10 ml of 90% methanol/10% water and dried on a vacuum concentrator (CentriVap). A typical purification from 30 g of ∆*nglO* cell paste yielded ~0.8 mg of **5** or 0.4 mg of **7**.

### Nostochloroside structural elucidation and derivatization

NMR spectra were collected using an Ascend 400 magnet equipped with a 5-mm CryoProbe Prodigy cryoprobe (Bruker) with the software TopSpin v4.0.9. Glycolipids **5** and **7** were dissolved in CD_3_OD (Cambridge Isotope Laboratories) and spectra were measured in 3-mm matched Shigemi tubes (Wilmad) using a 5X3INS-B 3-to-5-mm adapter (Norell).

For sugar identification, the sugar headgroup was acid-hydrolysed and then permethylated. For hydrolysis, 2 µl of a 24 mM solution of **5** dissolved in dimethylsulfoxide (DMSO) was added to 1 ml of 90% methanol/10% water with 1.2 M HCl. The solution was sealed and incubated at 80 °C for 21 h. The reaction was cooled to room temperature and dried in a vacuum concentrator (CentriVap). For permethylation, the dried product was dissolved in 200 µl of DMSO. Powdered NaOH (20 mg) was added and stirred for 10 min, then methyl iodide (30 µl) was added. The reaction was stirred for 30 min and then diluted with 1 ml of water. The reaction was extracted three times with 0.5 ml of dichloromethane (DCM). The combined DCM layers were washed three times with 1 ml of water, and the DCM was then dried in a vacuum concentrator (CentriVap) to yield the permethylated product. Permethylated sugar standards were prepared as previously described^72^. Briefly, 100 mg of a known sugar (d-glucose, d-galactose, d-mannose, d-talose, d-allose or l-gulose) was dissolved in 2 ml of DMSO. Powdered NaOH (444 mg, 4 equiv. per exchangeable H) was added and stirred vigorously. After 10 min, methyl iodide was added (690 µl, 4 equiv. per exchangeable H) and the reaction was stirred for 30 min. The reaction was then diluted with 10 ml of water and extracted three times with 10 ml of DCM. The combined DCM layers were washed three times with water and the DCM was dried on a rotary evaporator to afford the permethylated standard as a colourless or yellow oil. Permethylated products were dissolved in DCM and analysed by GC–MS on a Q Exactive GC Orbitrap system (Thermo) with a DB-5ms column (Agilent) with a length of 30 m. The GC oven was set to 50 °C, then to 200 °C at 3 °C min^−1^, then to 300 °C at 15 °C min^−1^, then held at 300 °C for 12 min. All transfer lines were at 310 °C. The injection volume was 1 µl.

To locate the O6 hydroxy group, 1 µl of a 24 mM solution of **5** dissolved in DMSO was added to 1 ml of DCM. Dess–Martin periodinane (11.4 mg) was added and the reaction was stirred for 2 h. The reaction was washed twice with 1 ml of saturated sodium bicarbonate and three times with water. The DCM layer was centrifuged for 3 min at 16,200*g* to facilitate phase separation, and the DCM was transferred to an oven-dried vial containing an oven-dried stir bar, and the DCM layer was dried on a rotary evaporator. *meta*-chloroperoxybenzoic acid (*m*CPBA, 14.8 mg, recrystallized from hot isopropanol) was added, and the vial was sealed and left under vacuum for 2 h. In a separate flask, 100 µl of BF_3_ diethyl etherate was added to 10 ml of anhydrous DCM under nitrogen. The vial containing *m*CPBA and **5** by-product was dissolved with 1 ml of the diluted BF_3_ diethyl etherate in DCM, and stirred overnight under nitrogen. The reaction was then extracted twice with saturated sodium bicarbonate and three times with water. The washed DCM layer was centrifuged for 2 min at 16,200*g*, transferred to a clean vial, and dried in a vacuum concentrator (CentriVap). The product was dissolved with 1 ml of THF and 0.5 ml of aqueous 1 M LiOH, then sealed and stirred vigorously overnight. The reaction was quenched with 0.5 ml of aqueous 2 M HCl and extracted four times with 0.5 ml of DCM. The combined DCM layers were washed four times with water, then dried in a vacuum concentrator (CentriVap), dissolved in 100 µl of methanol, and transferred to an HPLC vial for LC–MS analysis. The reaction products were analysed using the general analysis described above, except that the negative ionization mode was used, and the first 13.2 min were diverted to waste to avoid saturating the MS with salts and leftover reagents.

### Stable isotope feeding

Decanoic-*d*_19_ acid, lauric-*d*_23_ acid and stearic-*d*_35_ acid were from Sigma-Aldrich. Myristic-*d*_27_ acid was from Cambridge Isotope Laboratories. Palmitic-*d*_31_ acid was from Cayman Chemicals. Palmitic-6,6-*d*_2_ acid and palmitic-7,7-*d*_2_ acid were synthesized as described in the Supplementary Information. Labelled fatty acids were dissolved to 100 mM in DMSO and 25 µl was added to 25 ml of BG-11 (0.1 mM final concentration) in a 125-ml flask. The flask was inoculated with 0.25 ml of late-exponential to stationary-phase *N. punctiforme* ATCC 29133 strain ∆*nglO* (a >4-week-old culture) and incubated in the light for 21 days. Cultures were harvested and processed for metabolomics as described above.

### Protein structure prediction

Protein structure prediction was performed using the AlphaFold Colab notebook at https://colab.research.google.com/github/deepmind/alphafold/blob/main/notebooks/AlphaFold.ipynb using the default parameters.

### NglO cloning, expression, purification and assays

Expression vectors for N-terminal His_6_-tagged NglO were generated by Gibson assembly^65^. Vector pET-15b (Novagen) was linearized by PCR using primers GAGGATCCGGCTGCTAAC and GCCGCTGCTGTGATGATG. The product was digested with DpnI (New England Biolabs) for 1 h and purified using a QIAquick PCR Purification Kit (Qiagen). Full-length NglO was amplified using primers ATCATCATCACAGCAGCGGCAAAACAATCAAAAAGTCTACTAAGAAAACAATCG and TTGTTAGCAGCCGGATCCTCTTATACGATACGAAAATCACTGG. Truncated NglO was amplified using primers ATCATCATCACAGCAGCGGCAAAACAATCAAAAAGTCTACTAAGAAAACAATCG and TTGTTAGCAGCCGGATCCTCTTAAGTTGAAGATGGATTAAATGTTTCC. The PCR products were purified and assembled with the linearized pET-15b backbone using NEBuilder HiFi (New England Biolabs) to create vectors pET-NglO and pET-NglO′. The reaction products were transformed into *E. coli* Top10 and selected on LB agar with 50 µg ml^−1^ carbenicillin. Single colonies were grown in LB with 50 µg ml^−1^ carbenicillin, and plasmids were isolated using an E.Z.N.A. Plasmid DNA Mini Kit (Omega Bio-tek). Plasmid sequences were confirmed by Sanger sequencing and transformed into *E. coli* BL21(DE3) for expression and purification.

For expression, overnight cultures of *E. coli* BL21(DE3)/pET-NglO and *E. coli* BL21(DE3)/pET-NglO′ were grown in LB with 50 µg ml^−1^ carbenicillin, then a 250-µl volume was inoculated into 50 ml of terrific broth (TB, Difco) supplemented with 50 µg ml^−1^ carbenicillin and 5 mM MgSO_4_ in a 250-ml baffled flask. Cultures were grown at 37 °C while shaking at 200 r.p.m. (2.54-cm orbit diameter) to an optical density at 600 nm of 0.6–0.7. Expression was induced by adding 50 µl of 100 mM isopropyl β-d-1-thiogalactopyranoside. The incubator temperature was lowered to 16 °C and the cultures were incubated overnight.

For purification, cultures were collected by centrifuging for 10 min at 3,220*g*. All the following steps were performed using cold buffers at 0–4 °C on ice or in a cold room. The pellet was resuspended in 10 ml of binding buffer (20 mM HEPES, 300 mM NaCl, 30 mM imidazole, 10% glycerol, pH 8) and supplemented with 100 µl of 1 M MgCl_2_ and 0.1 M CaCl_2_. The culture was kept on ice and lysed by sonication (2 min total on time with a pulse sequence of 1 s on, 5 s off, 25% power level) and centrifuged at 15,000*g* for 30 min. The supernatant was decanted and 400 µl of a 50% Ni-NTA agarose slurry (Qiagen), washed beforehand with binding buffer, was added. The mixture was incubated for 1 h with gentle nutation and centrifuged for 1 min at 100*g*. The supernatant was decanted and the Ni-NTA resin was resuspended in the residual buffer, then transferred to a fritted spin column (Pierce). The resin was washed by gravity flow three times with 500 µl of binding buffer. The spin column was transferred to a clean 2-ml collection tube, and protein was eluted with two 300-µl portions of elution buffer (20 mM HEPES, 300 mM NaCl, 300 mM imidazole, 10% glycerol, pH 8). The eluted protein was used immediately as is for enzyme assays. Purification was confirmed using polyacrylamide gel electrophoresis followed by western blotting using the Penta·His antibody from Qiagen (product no. 34660, multiple lots, diluted 10,000-fold).

Assays were performed by adding 1 µl of a solution containing 250 mM MgCl_2_ and 250 mM CaCl_2_ to 25 µl of protein (0.6 mg ml^−1^ as determined by absorbance at 280 nm using a calculated extinction coefficient of 14,520 M^−1^ cm^−1^). The solution was mixed gently, and 0.5 µl of **5** and/or **7**, dissolved in DMSO at concentrations of 24 mM and 12 mM, respectively, was added and gently mixed. The reactions were incubated for 18 h at room temperature (22–23 °C), then quenched with 225 µl of methanol, incubated at −20 °C for 10 min, warmed back to room temperature, and centrifuged for 5 min at 16,200*g*. The supernatant was transferred to an HPLC vial for LC–MS analysis using the lipidomics method described above.

### Synthesis of palmitic-6,6-*d*_2_ acid and palmitic-7,7-*d*_2_ acid

Full synthetic procedures are available in the Supplementary Information.

### Reporting summary

Further information on research design is available in the Nature Portfolio Reporting Summary linked to this Article.

## Online content

Any methods, additional references, Nature Portfolio reporting summaries, source data, extended data, supplementary information, acknowledgements, peer review information; details of author contributions and competing interests; and statements of data and code availability are available at 10.1038/s41557-023-01390-z.

### Supplementary information

Supplementary Information Supplementary Figs. 1–22, Tables 1–9, Notes 1–3, synthetic procedures and references.

Reporting Summary

Supplementary Table Supplementary Table 1: Metabolomics of *C. licheniforme* ATCC 29412 comparing cultures grown without chloride to those with chloride. Negative fold changes represent metabolites which are depleted in the absence of chloride. *P* values were determined by a two-tailed Student’s *t*-test. Supplementary Table 2: Metabolomics of *N. punctiforme* ATCC 29133 comparing cultures grown without chloride to those with chloride. Metabolites were analysed using a generic method. Negative fold changes represent metabolites that are depleted in the absence of chloride. *P* values were determined by a two-tailed Student’s *t*-test. Supplementary Table 3: Metabolomics of *N. punctiforme* ATCC 29133 comparing cultures grown without chloride to those with chloride. Metabolites were analysed using a method optimized for lipids. Negative fold changes represent metabolites that are depleted in the absence of chloride. *P* values were determined by a two-tailed Student’s *t*-test. Supplementary Table 4: A replicate metabolomics experiment of *N. punctiforme* ATCC 29133 comparing cultures grown without chloride to those with chloride. Metabolites were analysed using a method optimized for lipids. Negative fold changes represent metabolites that are depleted in the absence of chloride. *P* values were determined by a two-tailed Student’s *t*-test. Supplementary Table 5: Metabolomics of *N. punctiforme* ATCC 29133 comparing the ∆*ngl* mutant to the wild type. Negative fold changes represent metabolites that are depleted in the absence of chloride. *P* values were determined by a two-tailed Student’s *t*-test. Supplementary Table 8: RNA-seq of *N. punctiforme* ATCC 29133 comparing cultures grown without chloride to those with chloride. Cultures were harvested after two weeks. Positive fold changes represent genes that are upregulated in the absence of chloride. *P* values (pvalue) were calculated with DESeq2 using the Wald test (pvalue) and corrected for multiple testing using the Benjamini and Hochberg method (padj). Supplementary Table 9: RNA-seq of *N. punctiforme* ATCC 29133 comparing cultures grown without chloride to those with chloride. Cultures were harvested after four weeks. Positive fold changes represent genes that are upregulated in the absence of chloride. *P* values (pvalue) were calculated with DESeq2 using the Wald test (pvalue) and corrected for multiple testing using the Benjamini–Hochberg method (padj).

### Source data

Source Data Fig. 3 Extracted ion chromatograms.

Source Data Fig. 4 Extracted ion chromatograms.

Source Data Fig. 5 Extracted ion chromatograms.

Source Data Extended Data Fig./Table 1 Extracted ion chromatograms.

Source Data Extended Data Fig./Table 2 Extracted ion chromatograms.

Source Data Extended Data Fig./Table 3 Extracted ion chromatograms.

Source Data Extended Data Fig./Table 4 Extracted ion chromatograms.

Source Data Extended Data Fig./Table 5 Extracted ion chromatograms and mass spectra.

Source Data Extended Data Fig./Table 6 Extracted ion chromatograms and mass spectra.

Source Data Extended Data Fig./Table 8 Statistical source data.

## Data Availability

RNA-sequencing data have been deposited in the Sequence Read Archive (http://www.ncbi.nlm.nih.gov/sra) under accession no. PRJNA868493. Raw LC–MS and LC–MS/MS data are available upon request due to the large file sizes. Previously published crystal structures are available in the Protein Data Bank (https://www.rcsb.org/) under accession codes 7RON and 7ROO. All other data are available in the manuscript or Supplementary Information. Source data are provided with this paper.

## References

[1] Newman DJ, Cragg GM (2020). Natural products as sources of new drugs over the nearly four decades from 01/1981 to 09/2019. J. Nat. Prod..

[2] van der Donk WA (2017). Introduction: unusual enzymology in natural product synthesis. Chem. Rev..

[3] Pye CR, Bertin MJ, Lokey RS, Gerwick WH, Linington RG (2017). Retrospective analysis of natural products provides insights for future discovery trends. Proc. Natl Acad. Sci. USA.

[4] Zani CL, Carroll AR (2017). Database for rapid dereplication of known natural products using data from MS and fast NMR experiments. J. Nat. Prod..

[5] Atanasov AG (2021). Natural products in drug discovery: advances and opportunities. Nat. Rev. Drug Discov..

[6] Li L, Maclntyre LW, Brady SF (2021). Refactoring biosynthetic gene clusters for heterologous production of microbial natural products. Curr. Opin. Biotechnol..

[7] Gerebtzoff G, Li-Blatter X, Fischer H, Frentzel A, Seelig A (2004). Halogenation of drugs enhances membrane binding and permeation. ChemBioChem.

[8] Wishart DS (2018). DrugBank 5.0: a major update to the DrugBank database for 2018. Nucleic Acids Res..

[9] Agarwal V (2017). Enzymatic halogenation and dehalogenation reactions: pervasive and mechanistically diverse. Chem. Rev..

[10] Gribble GW (2015). Biological activity of recently discovered halogenated marine natural products. Mar. Drugs.

[11] Latham J, Brandenburger E, Shepherd SA, Menon BRK, Micklefield J (2018). Development of halogenase enzymes for use in synthesis. Chem. Rev..

[12] Hornung A (2007). A genomic screening approach to the structure-guided identification of drug candidates from natural sources. ChemBioChem.

[13] Roullier C (2016). Automated detection of natural halogenated compounds from LC-MS profiles-application to the isolation of bioactive chlorinated compounds from marine-derived fungi. Anal. Chem..

[14] Peng H (2015). Untargeted identification of organo-bromine compounds in lake sediments by ultrahigh-resolution mass spectrometry with the data-independent precursor isolation and characteristic fragment method. Anal. Chem..

[15] Bromley CL (2018). Hyphenated LC-ICP-MS/ESI-MS identification of halogenated metabolites in South African marine ascidian extracts. S. Afr. J. Chem..

[16] Adak S, Moore BS (2021). Cryptic halogenation reactions in natural product biosynthesis. Nat. Prod. Rep..

[17] Vaillancourt FH, Yeh E, Vosburg DA, O’Connor SE, Walsh CT (2005). Cryptic chlorination by a non-haem iron enzyme during cyclopropyl amino acid biosynthesis. Nature.

[18] Gu L (2009). Metamorphic enzyme assembly in polyketide diversification. Nature.

[19] Yamanaka K, Ryan KS, Gulder TAM, Hughes CC, Moore BS (2012). Flavoenzyme-catalyzed atropo-selective *N*,*C*-bipyrrole homocoupling in marinopyrrole biosynthesis. J. Am. Chem. Soc..

[20] Marchand JA (2019). Discovery of a pathway for terminal-alkyne amino acid biosynthesis. Nature.

[21] Nakamura H, Hamer HA, Sirasani G, Balskus EP (2012). Cylindrocyclophane biosynthesis involves functionalization of an unactivated carbon center. J. Am. Chem. Soc..

[22] Nakamura H, Schultz EE, Balskus EP (2017). A new strategy for aromatic ring alkylation in cylindrocyclophane biosynthesis. Nat. Chem. Biol..

[23] Preisitsch M (2016). Effects of halide ions on the carbamidocyclophane biosynthesis in *Nostoc* sp. CAVN2. Mar. Drugs.

[24] Breinlinger S (2021). Hunting the eagle killer: a cyanobacterial neurotoxin causes vacuolar myelinopathy. Science.

[25] Schultz EE, Braffman NR, Luescher MU, Hager HH, Balskus EP (2019). Biocatalytic Friedel-Crafts alkylation using a promiscuous biosynthetic enzyme. Angew. Chem. Int. Ed..

[26] Preisitsch M (2016). Cylindrofridins A-C, linear cylindrocyclophane-related alkylresorcinols from the cyanobacterium *Cylindrospermum stagnale*. J. Nat. Prod..

[27] Guijas C (2018). METLIN: a technology platform for identifying knowns and unknowns. Anal. Chem..

[28] Aron AT (2020). Reproducible molecular networking of untargeted mass spectrometry data using GNPS. Nat. Protoc..

[29] Liaimer A (2011). A polyketide interferes with cellular differentiation in the symbiotic cyanobacterium *Nostoc punctiforme*. Environ. Microbiol. Rep..

[30] Campbell EL, Cohen MF, Meeks JC (1997). A polyketide-synthase-like gene is involved in the synthesis of heterocyst glycolipids in *Nostoc punctiforme* strain ATCC 29133. Arch. Microbiol..

[31] Swain M, Brisson J-R, Sprott GD, Cooper FP, Patel GB (1997). Identification of β-l-gulose as the sugar moiety of the main polar lipid of *Thermoplasma acidophilum*. Biochim. Biophys. Acta.

[32] Becker B, Melkonian M, Kamerling JP (1998). The cell wall (theca) of *Tetraselmis striata* (*Chlorophyta*): macromolecular composition and structural elements of the complex polysaccharides. J. Phycol..

[33] Umezawa H, Maeda K, Takeuchi T, Okami Y (1966). New antibiotics, bleomycin A and B. J. Antibiot..

[34] Reis JPA, Figueiredo SAC, Sousa ML, Leão PN (2020). BrtB is an O-alkylating enzyme that generates fatty acid-bartoloside esters. Nat. Commun..

[35] Jumper J (2021). Highly accurate protein structure prediction with AlphaFold. Nature.

[36] Baumann U (2019). Structure-function relationships of the repeat domains of RTX toxins. Toxins.

[37] Guerra JVDS (2020). ParKVFinder: a thread-level parallel approach in biomolecular cavity detection. SoftwareX.

[38] Guerra JVDS (2021). pyKVFinder: an efficient and integrable Python package for biomolecular cavity detection and characterization in data science. BMC Bioinformatics.

[39] Campbell EL, Summers ML, Christman H, Martin ME, Meeks JC (2007). Global gene expression patterns of *Nostoc punctiforme* in steady-state dinitrogen-grown heterocyst-containing cultures and at single time points during the differentiation of akinetes and hormogonia. J. Bacteriol..

[40] Risser Douglas D, Wong Francis CY, Meeks John C (2012). Biased inheritance of the protein PatN frees vegetative cells to initiate patterned heterocyst differentiation. Proc. Natl Acad. Sci. USA.

[41] Balskus Emily P, Walsh Christopher T (2010). The genetic and molecular basis for sunscreen biosynthesis in cyanobacteria. Science.

[42] Gao Q, Garcia-Pichel F (2011). An ATP-grasp ligase involved in the last biosynthetic step of the iminomycosporine shinorine in *Nostoc punctiforme* ATCC 29133. J. Bacteriol..

[43] Seyedsayamdost MR (2014). High-throughput platform for the discovery of elicitors of silent bacterial gene clusters. Proc. Natl Acad. Sci. USA.

[44] Ongley SE, Bian X, Neilan BA, Müller R (2013). Recent advances in the heterologous expression of microbial natural product biosynthetic pathways. Nat. Prod. Rep..

[45] Kleigrewe K (2015). Combining mass spectrometric metabolic profiling with genomic analysis: a powerful approach for discovering natural products from cyanobacteria. J. Nat. Prod..

[46] Meeks JC (2001). An overview of the genome of *Nostoc punctiforme*, a multicellular, symbiotic cyanobacterium. Photosynth. Res..

[47] Dehm D (2019). Unlocking the spatial control of secondary metabolism uncovers hidden natural product diversity in *Nostoc punctiforme*. ACS Chem. Biol..

[48] Roeßler M, Sewald X, Müller V (2003). Chloride dependence of growth in bacteria. FEMS Microbiol. Lett..

[49] Braffman NR (2022). Structural basis for an unprecedented enzymatic alkylation in cylindrocyclophane biosynthesis. eLife.

[50] Murry MA, Wolk CP (1989). Evidence that the barrier to the penetration of oxygen into heterocysts depends upon two layers of the cell envelope. Arch. Microbiol..

[51] Caforio A, Driessen AJM (2017). Archaeal phospholipids: structural properties and biosynthesis. Biochim. Biophys. Acta.

[52] Braverman NE, Moser AB (2012). Functions of plasmalogen lipids in health and disease. Biochim. Biophys. Acta.

[53] Wolucka Beata A (2001). Partial purification and identification of GDP-mannose 3′,5′-epimerase of *Arabidopsis thaliana*, a key enzyme of the plant vitamin C pathway. Proc. Natl Acad. Sci. USA.

[54] Chang Z (2002). The barbamide biosynthetic gene cluster: a novel marine cyanobacterial system of mixed polyketide synthase (PKS)-non-ribosomal peptide synthetase (NRPS) origin involving an unusual trichloroleucyl starter unit. Gene.

[55] Hicks LM, Moffitt MC, Beer LL, Moore BS, Kelleher NL (2006). Structural characterization of in vitro and in vivo intermediates on the loading module of microcystin synthetase. ACS Chem. Biol..

[56] Yore MM (2014). Discovery of a class of endogenous mammalian lipids with anti-diabetic and anti-inflammatory effects. Cell.

[57] Williams DE, Sturgeon CM, Roberge M, Andersen RJ (2007). Nigricanosides A and B, antimitotic glycolipids isolated from the green alga *Avrainvillea nigricans* collected in Dominica. J. Am. Chem. Soc..

[58] Peisl BYL, Schymanski EL, Wilmes P (2018). Dark matter in host-microbiome metabolomics: tackling the unknowns—a review. Anal. Chim. Acta.

[59] Pluskal T, Castillo S, Villar-Briones A, Oresic M (2010). MZmine 2: modular framework for processing, visualizing and analyzing mass spectrometry-based molecular profile data. BMC Bioinformatics.

[60] Myers OD, Sumner SJ, Li S, Barnes S, Du X (2017). One step forward for reducing false positive and false negative compound identifications from mass spectrometry metabolomics data: new algorithms for constructing extracted ion chromatograms and detecting chromatographic peaks. Anal. Chem..

[61] Hunter JD (2007). Matplotlib: a 2D graphics environment. Comput. Sci. Eng..

[62] Waskom ML (2021). seaborn: statistical data visualization. J. Open Source Softw..

[63] Olivon F (2018). MetGem software for the generation of molecular networks based on the *t*-SNE algorithm. Anal. Chem..

[64] Elie N, Santerre C, Touboul D (2019). Generation of a molecular network from electron ionization mass spectrometry data by combining MZmine2 and MetGem software. Anal. Chem..

[65] Gibson DG (2009). Enzymatic assembly of DNA molecules up to several hundred kilobases. Nat. Methods.

[66] Black TA, Cai Y, Wolk CP (1993). Spatial expression and autoregulation of *hetR*, a gene involved in the control of heterocyst development in *Anabaena*. Mol. Microbiol..

[67] Liang J, Scappino L, Haselkorn R (1993). The patB gene product, required for growth of the cyanobacterium *Anabaena* sp. strain PCC 7120 under nitrogen-limiting conditions, contains ferredoxin and helix-turn-helix domains. J. Bacteriol..

[68] Langmead B, Salzberg SL (2012). Fast gapped-read alignment with Bowtie 2. Nat. Methods.

[69] Liao Y, Smyth GK, Shi W (2014). featureCounts: an efficient general purpose program for assigning sequence reads to genomic features. Bioinformatics.

[70] Love MI, Huber W, Anders S (2014). Moderated estimation of fold change and dispersion for RNA-seq data with DESeq2. Genome Biol..

[71] Khayatan B, Meeks JC, Risser DD (2015). Evidence that a modified type IV pilus-like system powers gliding motility and polysaccharide secretion in filamentous cyanobacteria. Mol. Microbiol..

[72] Ciucanu I, Kerek F (1984). A simple and rapid method for the permethylation of carbohydrates. Carbohydr. Res..

