# Supplementary material for: Accelerating the discovery of alkyl halide-derived natural products using halide depletion
Source: Nat Chem. 2024 Jan 12;16(2):173–82. doi: 10.1038/s41557-023-01390-z (PMC10849952; doi:10.1038/s41557-023-01390-z)
Supplement: Supplementary file 1 — Supplementary Figs. 1–22, Tables 1–9, Notes 1–3, synthetic procedures and references. [file 41557_2023_1390_MOESM1_ESM.pdf]

nature chemistry

Article

https://doi.org/10.1038/s41557-023-01390-z

Accelerating the discovery of alkyl halide-derived natural products using halide depletion

In the format provided by the authors and unedited

Supplementary Information

Supplementary Fig. 1 | Discovery of new cylindrocyclophane derivatives 2
Supplementary Fig. 2 | MS1 spectra of compounds in the cylindrocyclophane molecular network 3
Supplementary Fig. 3 | ^{1}H spectrum of **5** in CD_3OD at 400 MHz 4
Supplementary Fig. 4 | ^{13}C spectrum of **5** in CD_3OD at 101 MHz 5
Supplementary Fig. 5 | ^{1}H–^{13}C HSQC spectrum of **5** in CD_3OD at 400 MHz 6
Supplementary Fig. 6 | ^{1}H–^{13}C HMBC spectrum of **5** in CD_3OD at 400 MHz 7
Supplementary Fig. 7 | ^{1}H–^{13}C H2BC spectrum of **5** in CD_3OD at 400 MHz 8
Supplementary Fig. 8 | ^{1}H–^{1}H COSY spectrum of **5** in CD_3OD at 400 MHz 9
Supplementary Fig. 9 | ^{1}H–^{1}H TOCSY spectrum of **5** in CD_3OD at 400 MHz 10
Supplementary Fig. 10 | ^{1}H–^{13}C HSQC-TOCSY spectrum of **5** in CD_3OD at 400 MHz 11
Supplementary Fig. 11 | ^{1}H–^{1}H NOESY spectrum of **5** in CD_3OD at 400 MHz 12
Supplementary Fig. 12 | ^{1}H spectrum of **7** in CD_3OD at 400 MHz 13
Supplementary Fig. 13 | ^{13}C spectrum of **7** in CD_3OD at 101 MHz 14
Supplementary Fig. 14 | ^{1}H–^{13}C HSQC spectrum of **7** in CD_3OD at 400 MHz 15
Supplementary Fig. 15 | ^{1}H–^{13}C HMBC spectrum of **7** in CD_3OD at 400 MHz 16
Supplementary Fig. 16 | ^{1}H–^{13}C H2BC spectrum of **7** in CD_3OD at 400 MHz 17
Supplementary Fig. 17 | ^{1}H–^{1}H COSY spectrum of **7** in CD_3OD at 400 MHz 18
Supplementary Fig. 18 | ^{1}H–^{1}H TOCSY spectrum of **7** in CD_3OD at 400 MHz 19
Supplementary Fig. 19 | ^{1}H–^{13}C HSQC-TOCSY spectrum of **7** in CD_3OD at 400 MHz 20
Supplementary Fig. 20 | ^{1}H–^{1}H NOESY spectrum of **7** in CD_3OD at 400 MHz 21
Supplementary Fig. 21 | SDS-PAGE gels illustrating purification of NglO′ 22
Supplementary Fig. 22 | Biosynthetic hypothesis for the acyl chain of **5** 23
Supplementary Table 6 | Partial NMR assignments for **5** in CD_3OD 24
Supplementary Table 7 | Partial NMR assignments for **7** in CD_3OD 25
Supplementary Table 10 | Template search for homology modeling of NglG 26
Supplementary Note 1 | NMR analysis of **5** and **7** 27
Supplementary Note 2 | Isotopic labeling of **5** 29
Supplementary Note 3 | Biosynthetic hypothesis 31
Synthetic Procedures 32
Supplementary References 39

a

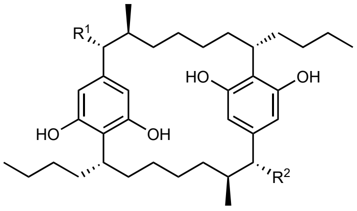

cylindrocyclophanes A–F

	R^1	R^2	Formula
cylindrocyclophane A	OH	OH	$C_{36}H_{56}O_6$
cylindrocyclophane B	OH	OAc	$C_{38}H_{58}O_7$
cylindrocyclophane C	OH	H	$C_{36}H_{56}O_5$
cylindrocyclophane D	OAc	OAc	$C_{40}H_{60}O_8$
cylindrocyclophane E	OAc	H	$C_{38}H_{58}O_6$
cylindrocyclophane F	H	H	$C_{36}H_{56}O_4$

b

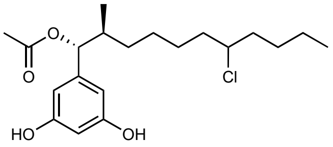

cylindrofridin A ($C_{20}H_{31}O_4Cl$)

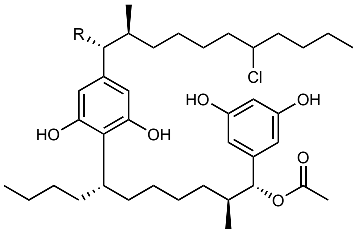

cylindrofridins B and C

	R	Formula
cylindrofridin B	OH	$C_{38}H_{59}O_7$
cylindrofridin C	OH	$C_{40}H_{61}O_8$

c

Feature	*m*/*z*	Predicted Ion	Proposed Identity
A (**4**)	553.4253	$[C_{36}H_{57}O_4]^+$	cylindrocyclophane F
B	551.4097	$[C_{36}H_{55}O_4]^+$	desaturated cylindrocyclophane F
C	551.4097	$[C_{36}H_{55}O_4]^+$	desaturated cylindrocyclophane F
D	609.4170	$[C_{38}H_{57}O_6]^+$	desaturated cylindrocyclophane E
E (**3**)	589.4013	$[C_{36}H_{58}O_4Cl]^+$	non-acetylated cylindrofridin B
F	567.4146	$[C_{36}H_{55}O_5]^+$	desaturated cylindrocyclophane C
G	567.4130	$[C_{36}H_{55}O_5]^+$	desaturated cylindrocyclophane C
H*	549.3947	$[C_{36}H_{53}O_4]^+$	source fragment of J
I*	609.4147	$[C_{38}H_{57}O_6]^+$	source fragment of J
J*	686.4661	$[C_{40}H_{64}NO_8]^+$	cylindrocyclophane D, ammonium adduct
K	776.5097	$[C_{43}H_{70}NO_{11}]^+$ or $[C_{46}H_{66}N_5O_7]^+$	uncharacterized cylindrocyclophane
L	565.3916	$[C_{36}H_{53}O_5]^+$	2× desaturated cylindrocyclophane C
M	565.3893	$[C_{36}H_{53}O_5]^+$	2× desaturated cylindrocyclophane C
N	571.4012	$[C_{35}H_{55}O_6]^+$	desaturated cylindrocyclophane A
O	607.3782	$[C_{35}H_{56}O_6Cl]^+$	uncharacterized cylindrofridin
P**	569.3917	$[C_{35}H_{53}O_6]^+$	source fragment of Q
Q**	646.4327	$[C_{37}H_{57}O_8]^+$ or $[C_{38}H_{53}N_4O_4]^+$	uncharacterized cylindrocyclophane
R	585.3811	$[C_{37}H_{61}O_5]^+$	uncharacterized cylindrocyclophane
S***	715.4766	$[C_{42}H_{67}O_9]^+$ or $[C_{38}H_{63}N_6O_7]^+$	source fragment of T
T***	894.5555	$[C_{55}H_{80}NO_{14}]^+$ or $[C_{44}H_{76}N_7O_{12}]^+$	uncharacterized cylindrocyclophane
U	603.4180	$[C_{37}H_{60}O_4Cl]^+$	uncharacterized cylindrofridin
V	605.3984	$[C_{36}H_{58}O_5Cl]^+$	uncharacterized cylindrofridin
W	399.2378	$[C_{21}H_{35}O_7]^+$ or $[C_{22}H_{31}N_4O_3]^+$	uncharacterized monoalkylresorcinol
X (**2**)	329.1880	$[C_{18}H_{30}O_3Cl]^+$	monoalkylresorcinol monomer
Y	371.1989	$[C_{20}H_{32}O_4Cl]^+$	cylindrofridin A

Supplementary Fig. 1 | Discovery of new cylindrocyclophane derivatives

a, Structures of known cylindrocyclophanes. **b**, Structures of known cylindrofridins. **c**, Proposed identities of the species in the cylindrocyclophane molecular network (Fig. 2). The molecular formulas were predicted from the observed *m/z* values and the MS1 spectrum using the *Generate Formulas from Compound* feature in Qualitative Analysis (Agilent). The proposed identity of each species was derived by comparing the predicted formula to the most similar known cylindrocyclophane or cylindrofridin. Source fragments were identified by overlapping peak shapes and retention times.

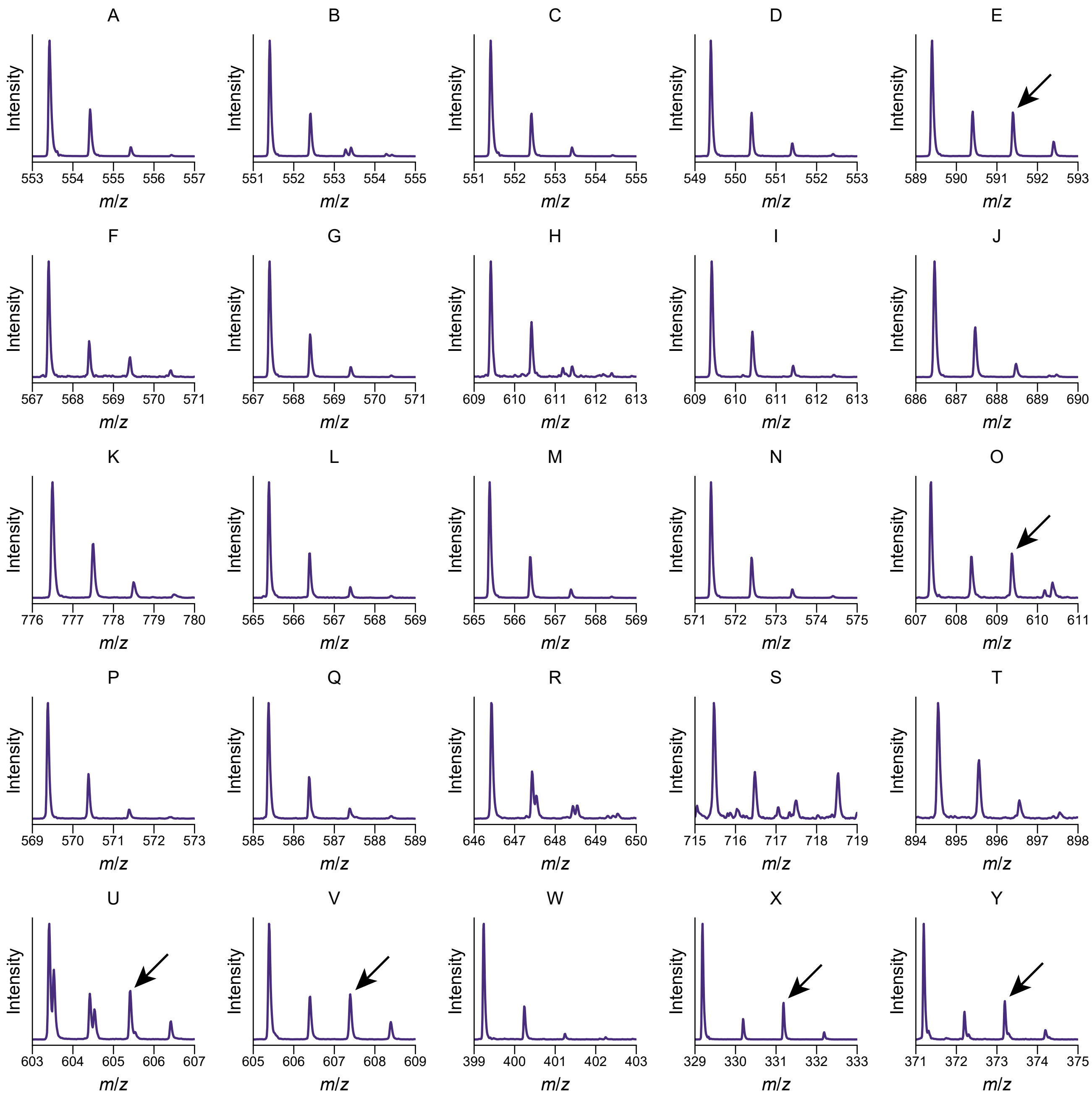

Supplementary Fig. 2 | MS1 spectra of compounds in the cylindrocyclophane molecular network

Each panel shows the MS1 spectrum for the feature indicated above, corresponding to the features detected in the cylindrocyclophane molecular network (Fig. 2d, e). Arrows denote the increased intensity at M+2 that is diagnostic of halogenated compounds, in this case arising primarily from the relative abundance of ^{35}Cl and ^{37}Cl.

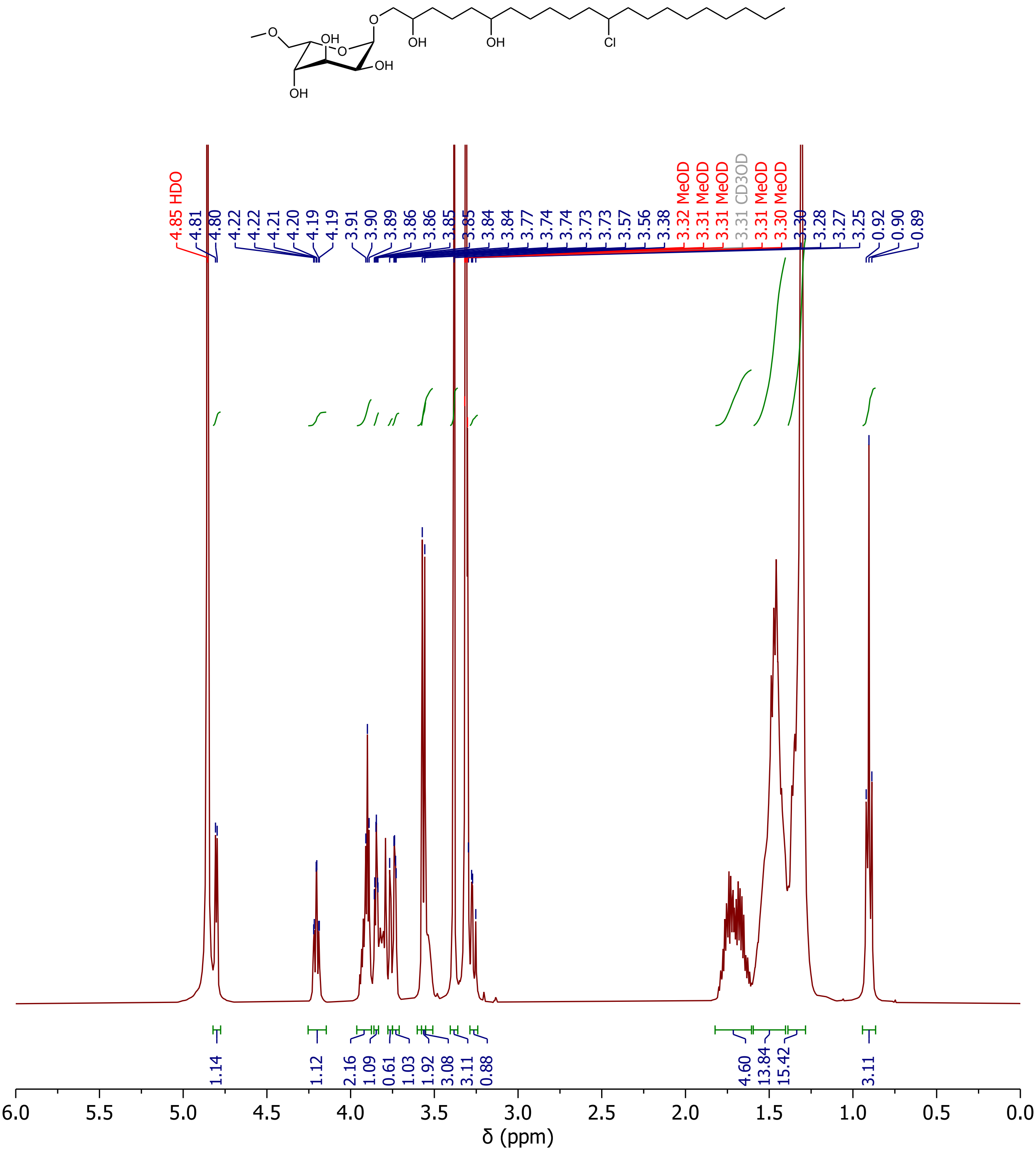

Supplementary Fig. 3 | ^{1}H spectrum of 5 in CD_3OD at 400 MHz

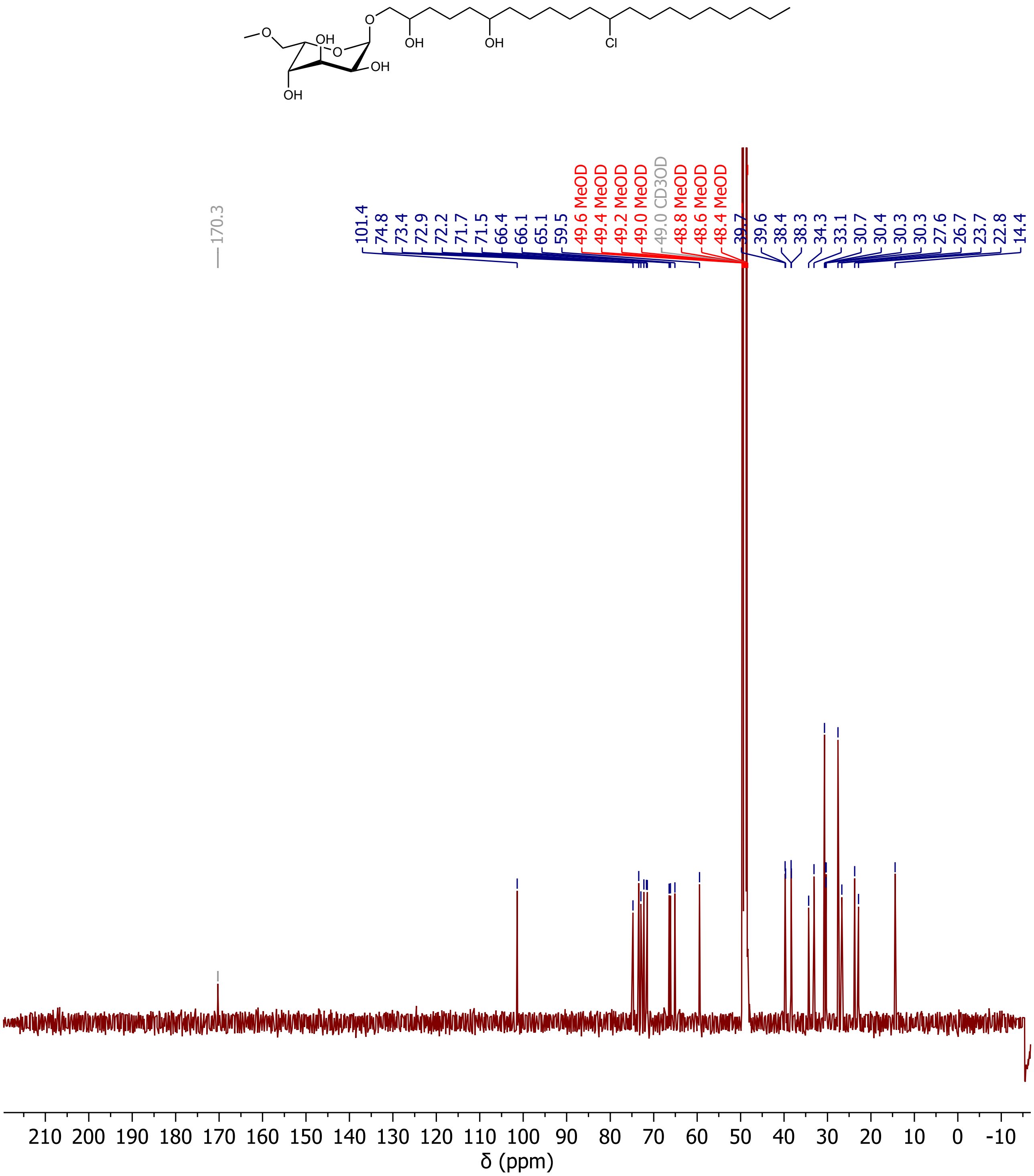

Supplementary Fig. 4 | ^{13}C spectrum of 5 in CD_3OD at 101 MHz

The peak at 170.3 ppm likely originates from trace formic acid, used as a mobile phase additive during chromatography, that remained tightly bound to the sample.

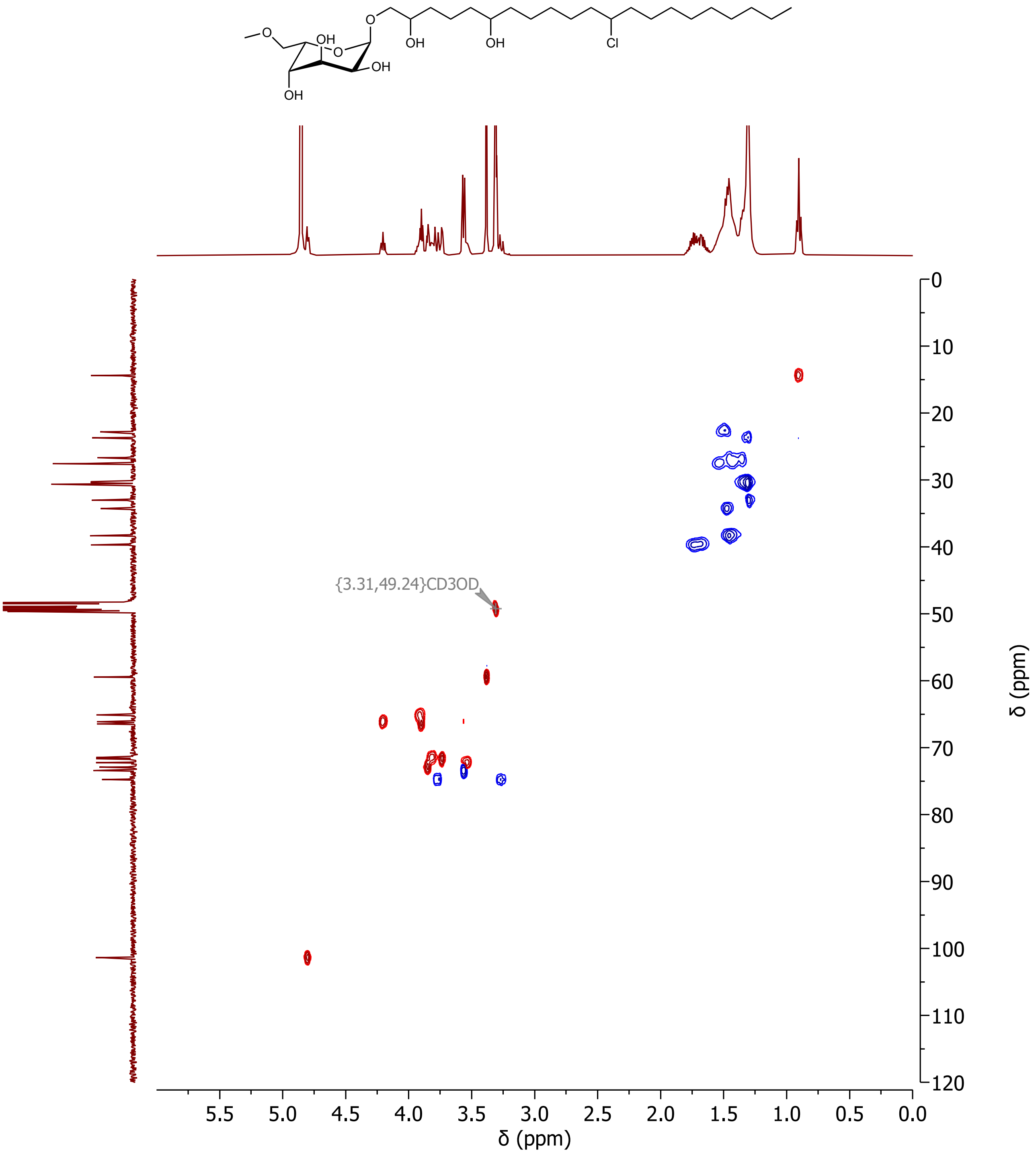

Supplementary Fig. 5 | ^{1}H–^{13}C HSQC spectrum of 5 in CD_3OD at 400 MHz

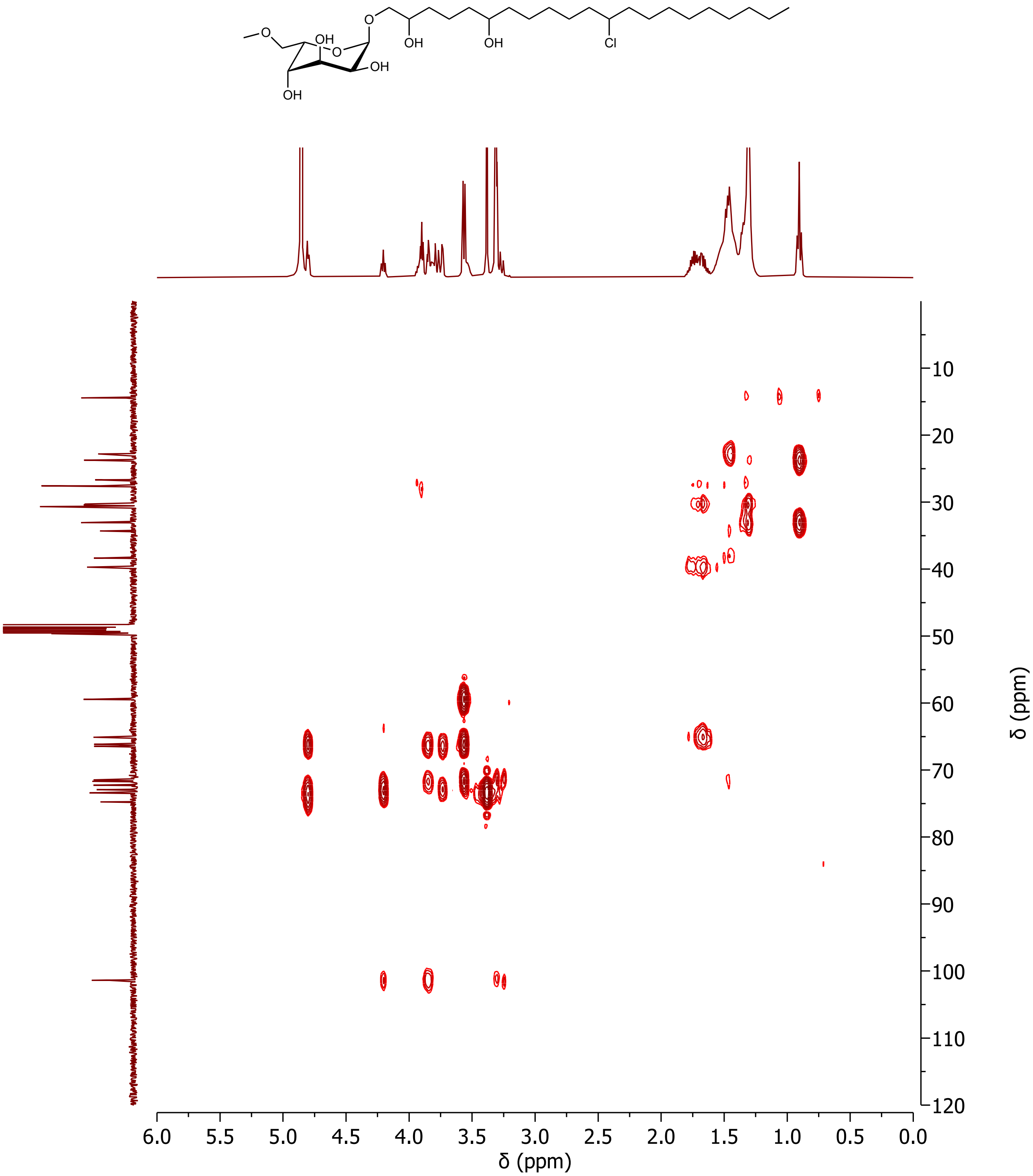

Supplementary Fig. 6 | ^{1}H–^{13}C HMBC spectrum of 5 in CD_3OD at 400 MHz

The coupling constant (cnst13) was 5 Hz.

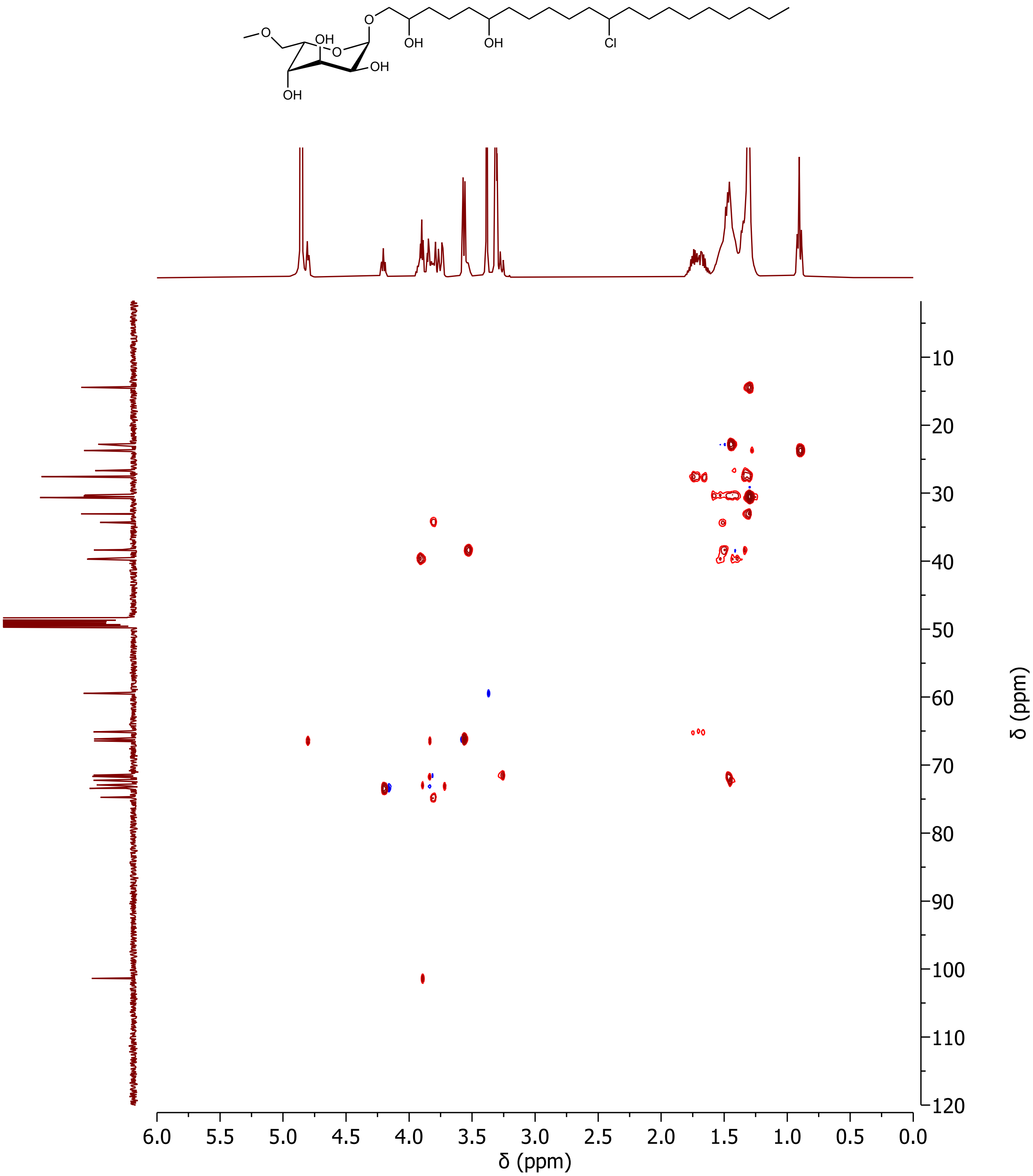

Supplementary Fig. 7 | ^{1}H–^{13}C H2BC spectrum of 5 in CD_3OD at 400 MHz

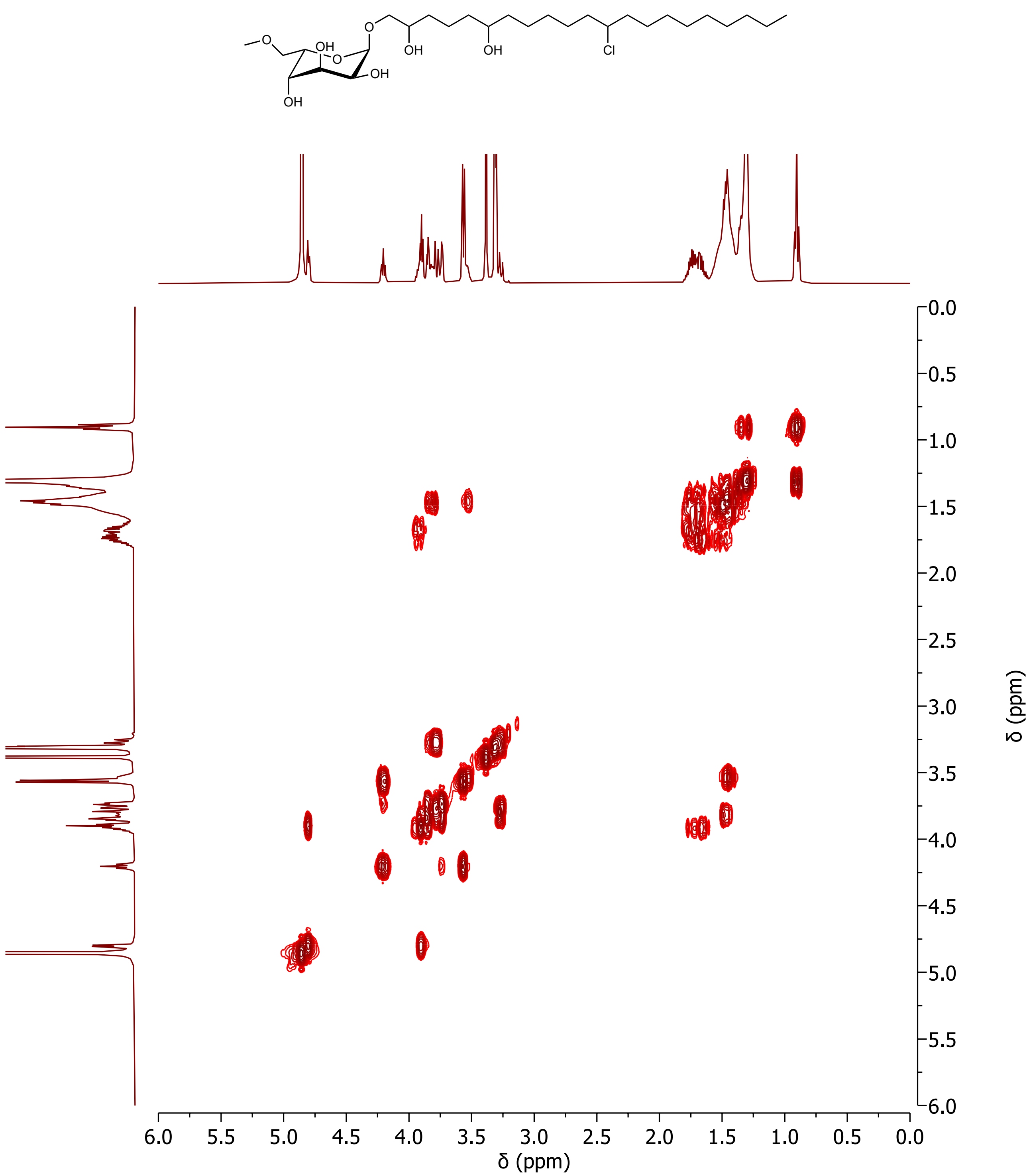

Supplementary Fig. 8 | ^{1}H–^{1}H COSY spectrum of 5 in CD_3OD at 400 MHz

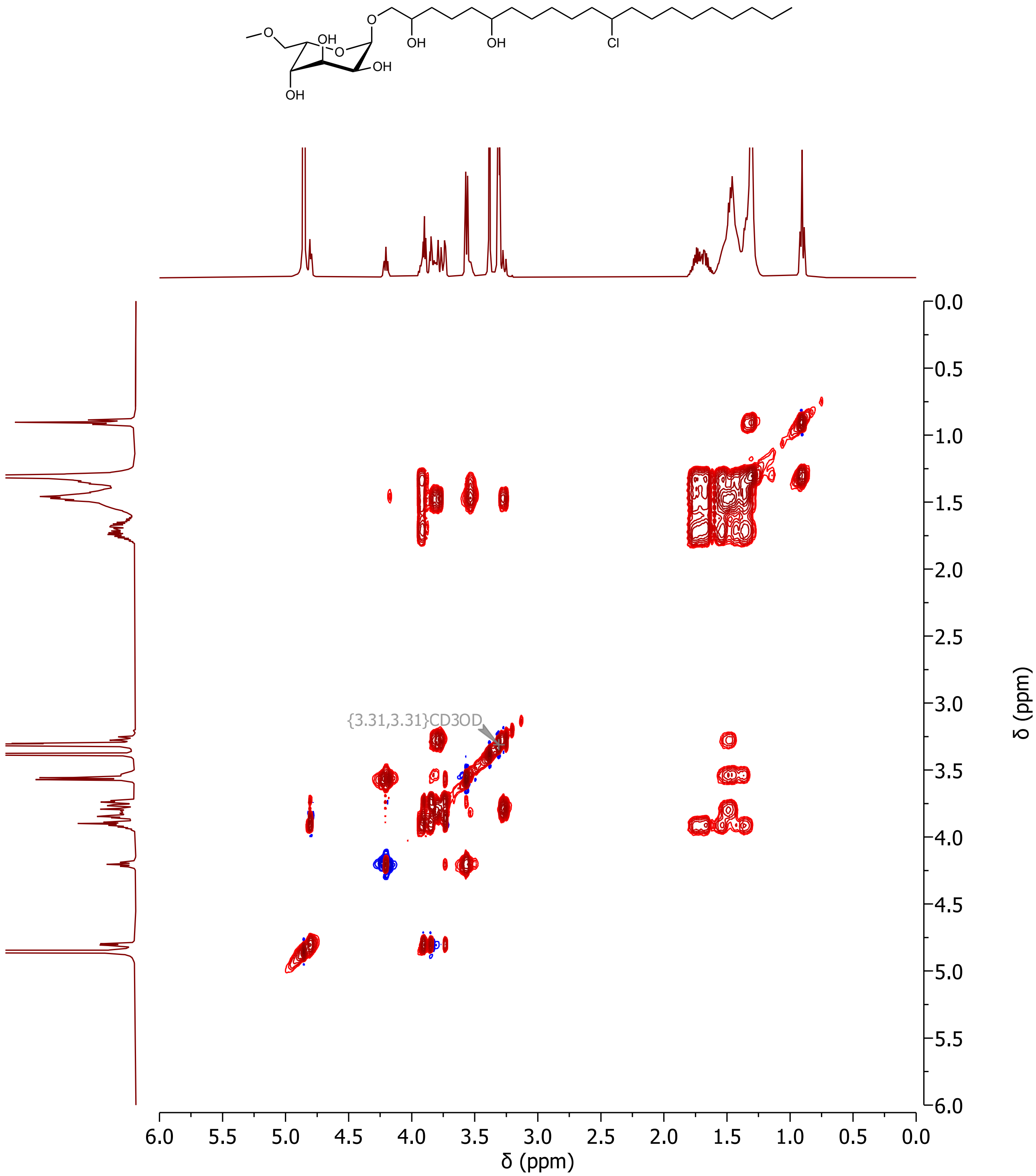

Supplementary Fig. 9 | ^{1}H–^{1}H TOCSY spectrum of 5 in CD_3OD at 400 MHz

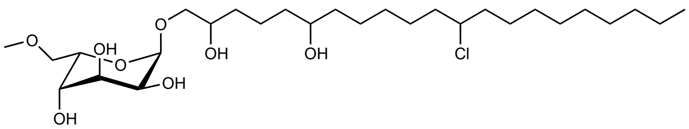

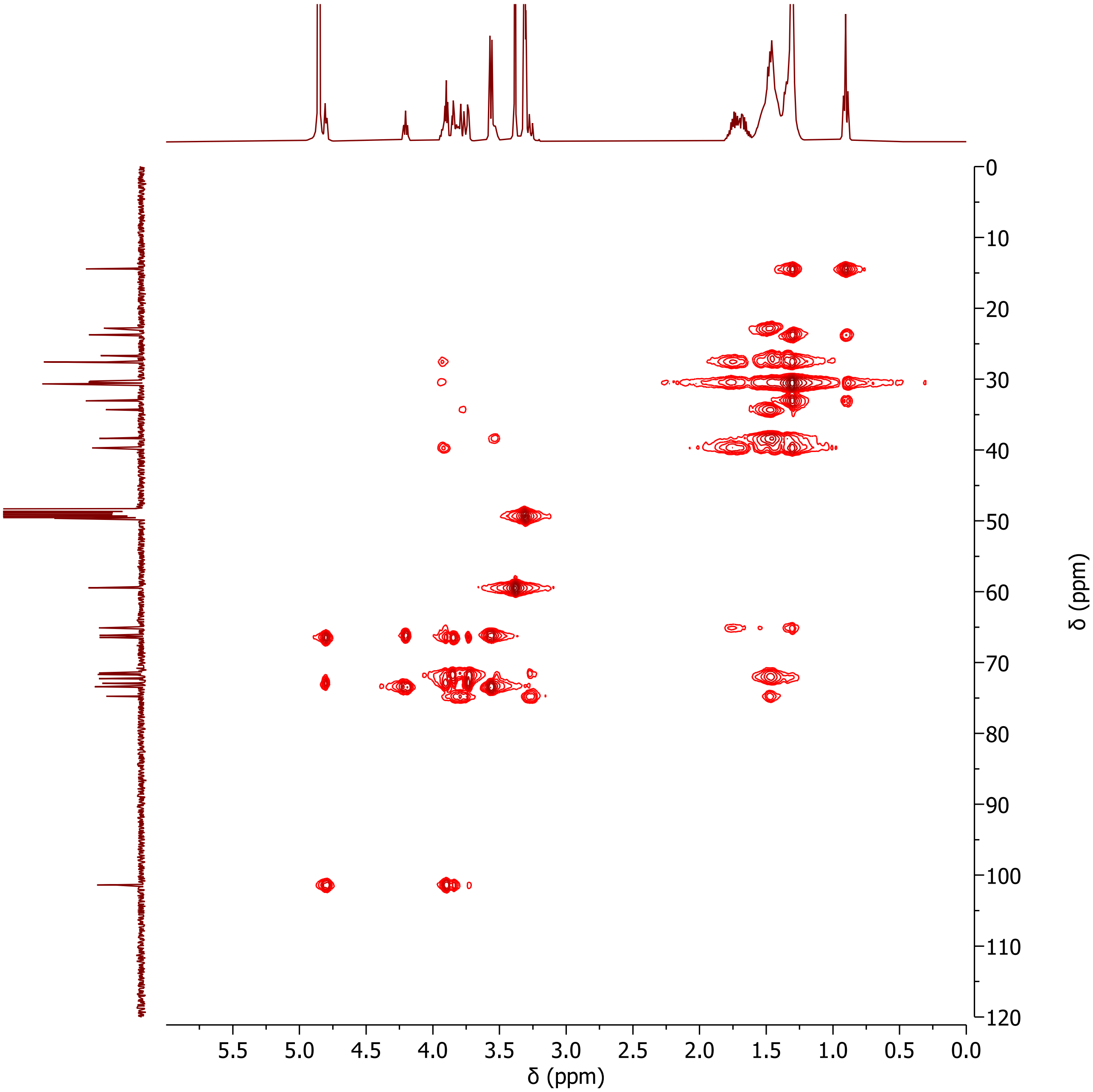

Supplementary Fig. 10 | ^{1}H–^{13}C HSQC-TOCSY spectrum of 5 in CD_3OD at 400 MHz

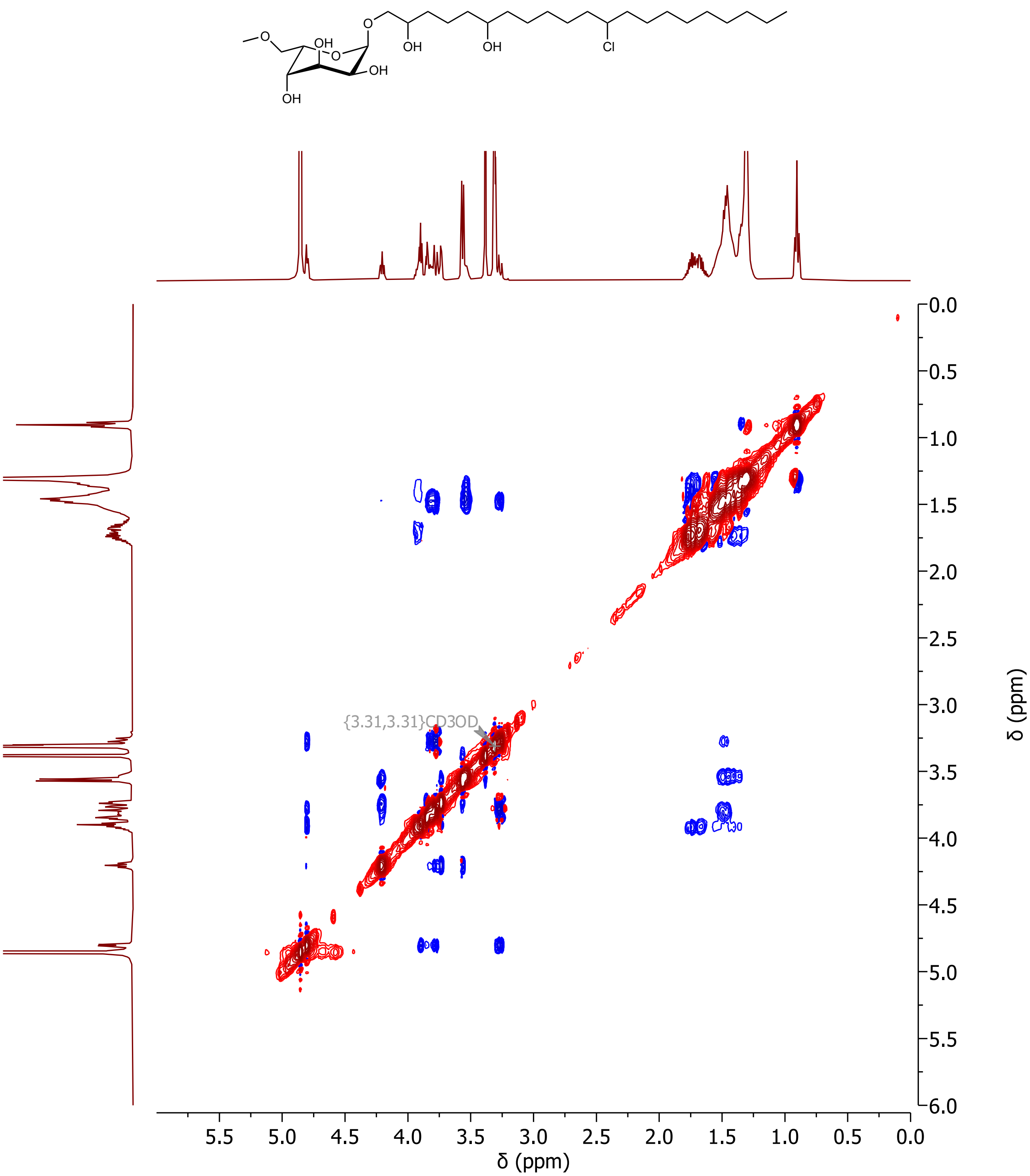

Supplementary Fig. 11 | ^{1}H–^{1}H NOESY spectrum of 5 in CD_3OD at 400 MHz

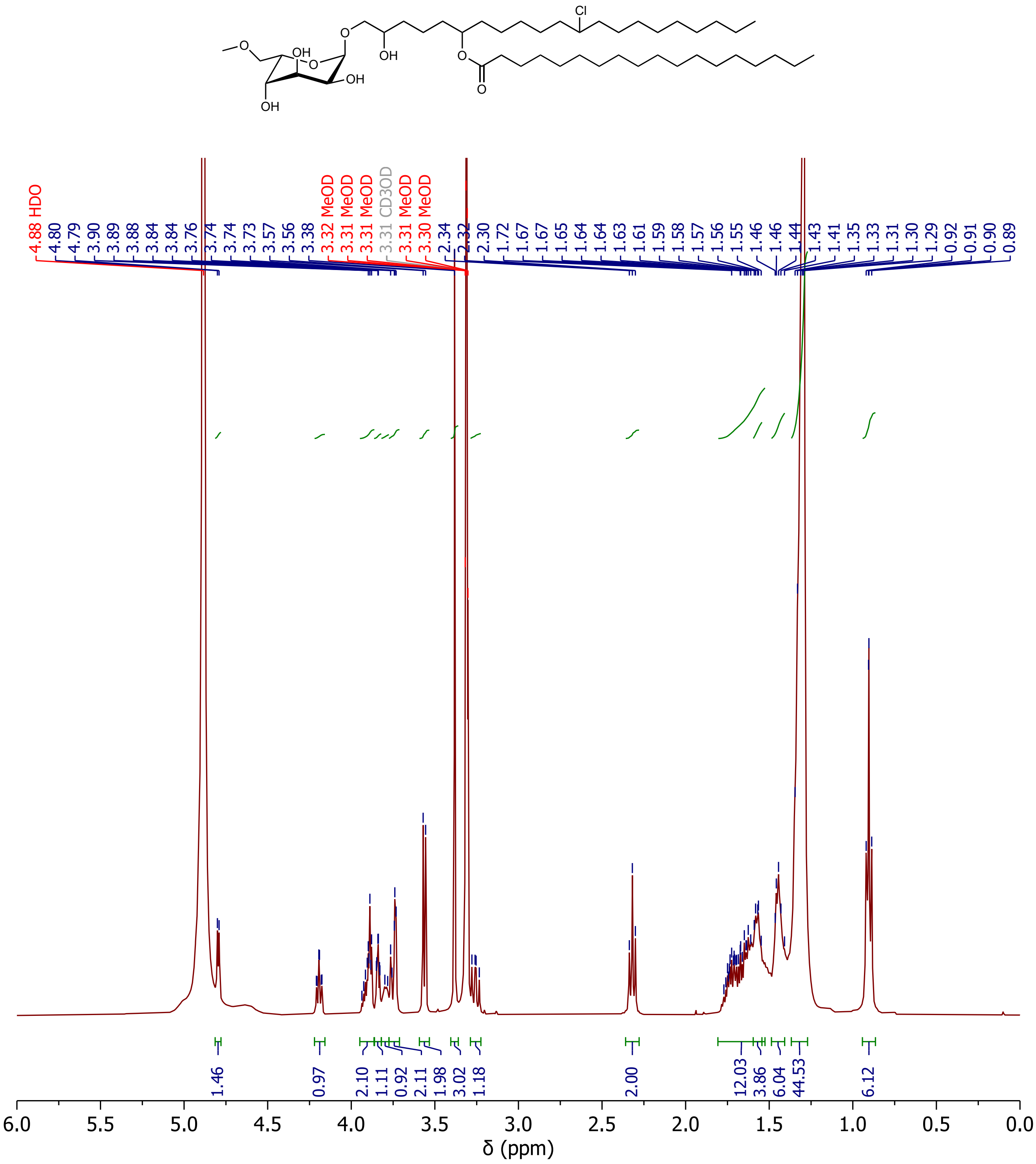

Supplementary Fig. 12 | ^{1}H spectrum of 7 in CD_3OD at 400 MHz

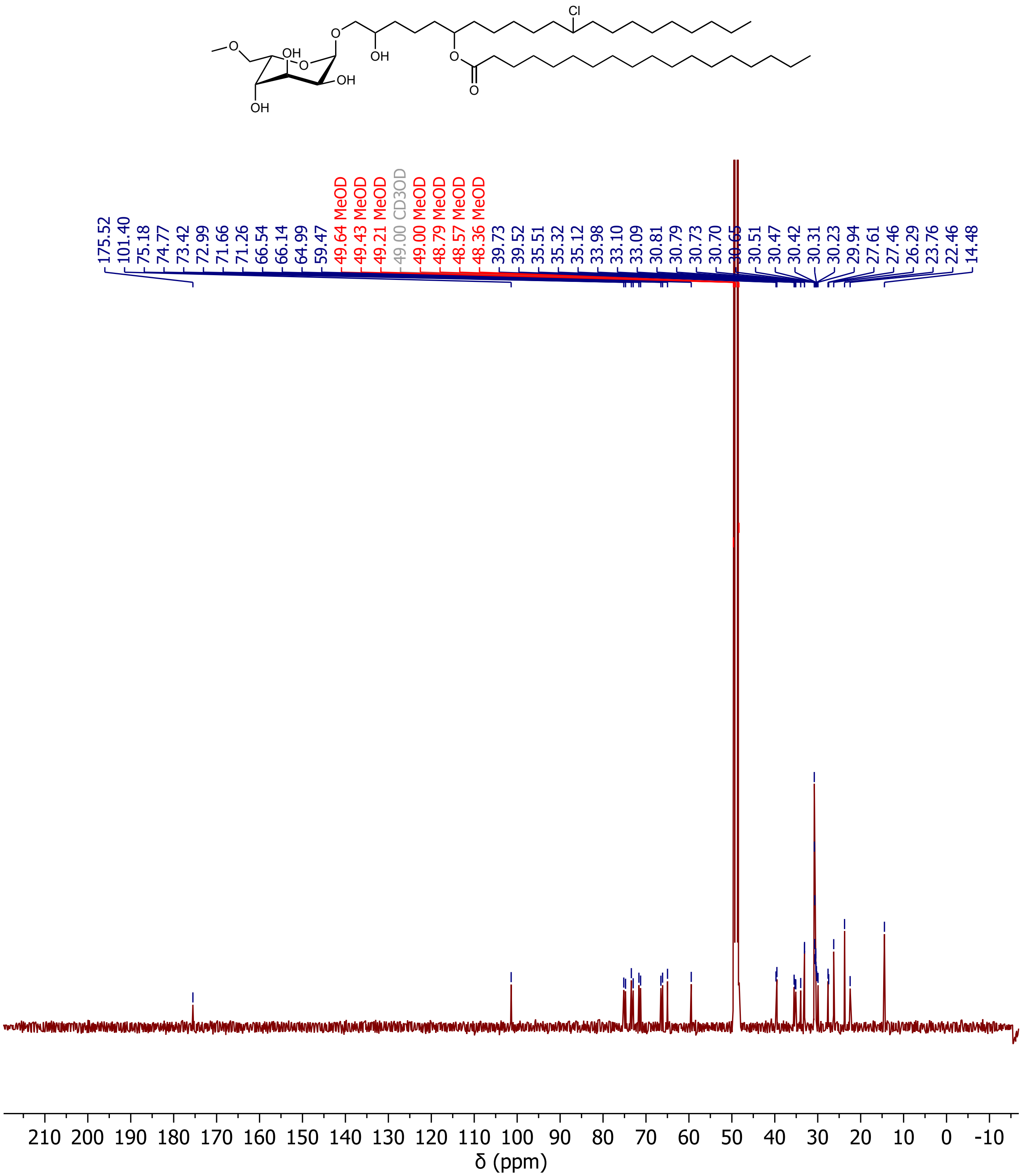

Supplementary Fig. 13 | ^{13}C spectrum of 7 in CD_3OD at 101 MHz

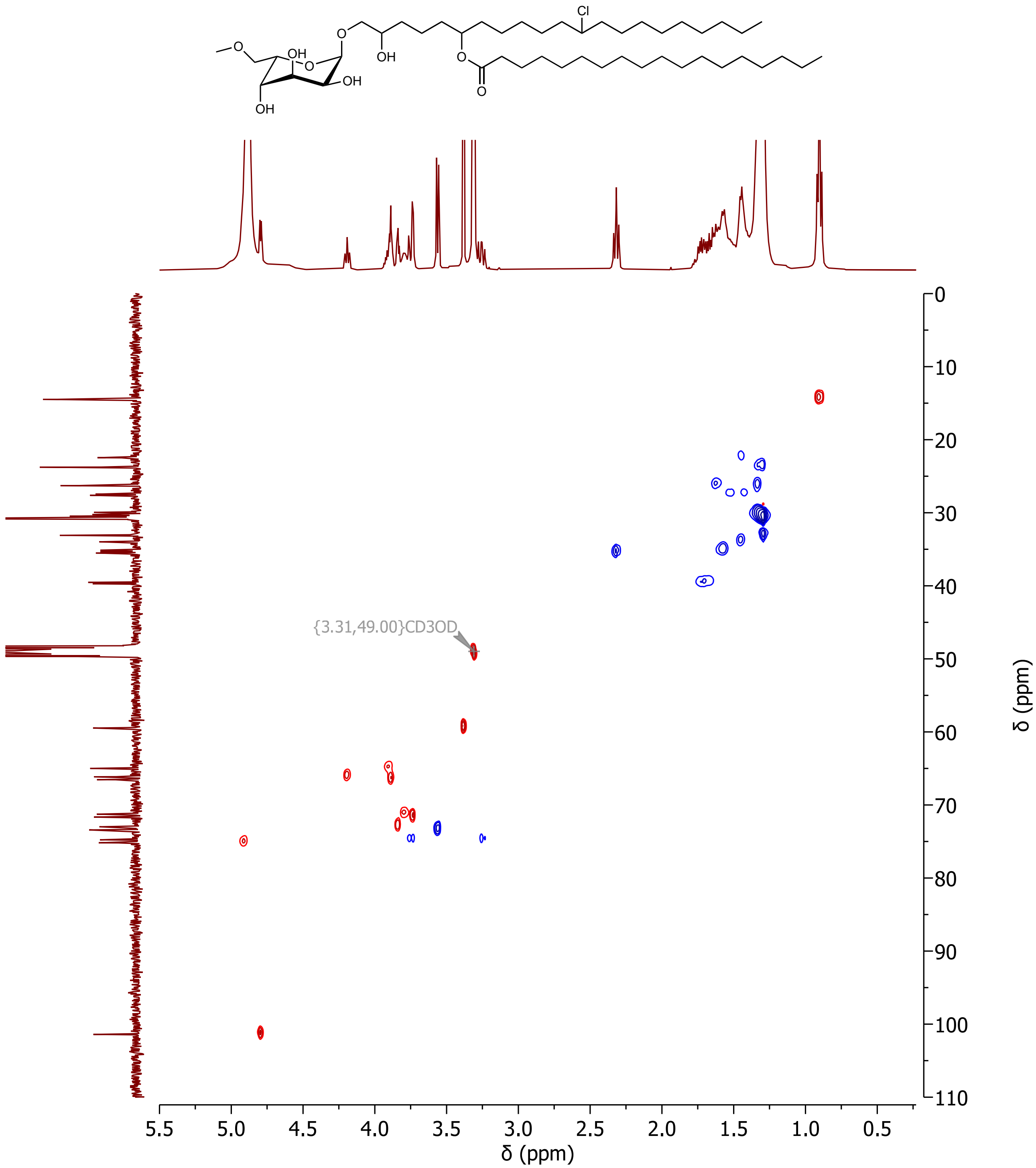

Supplementary Fig. 14 | ^{1}H–^{13}C HSQC spectrum of 7 in CD_3OD at 400 MHz

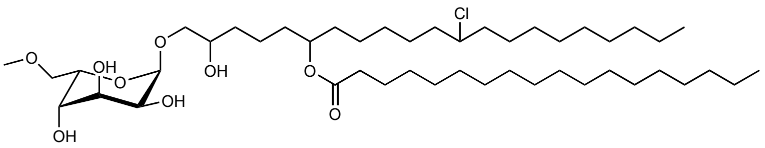

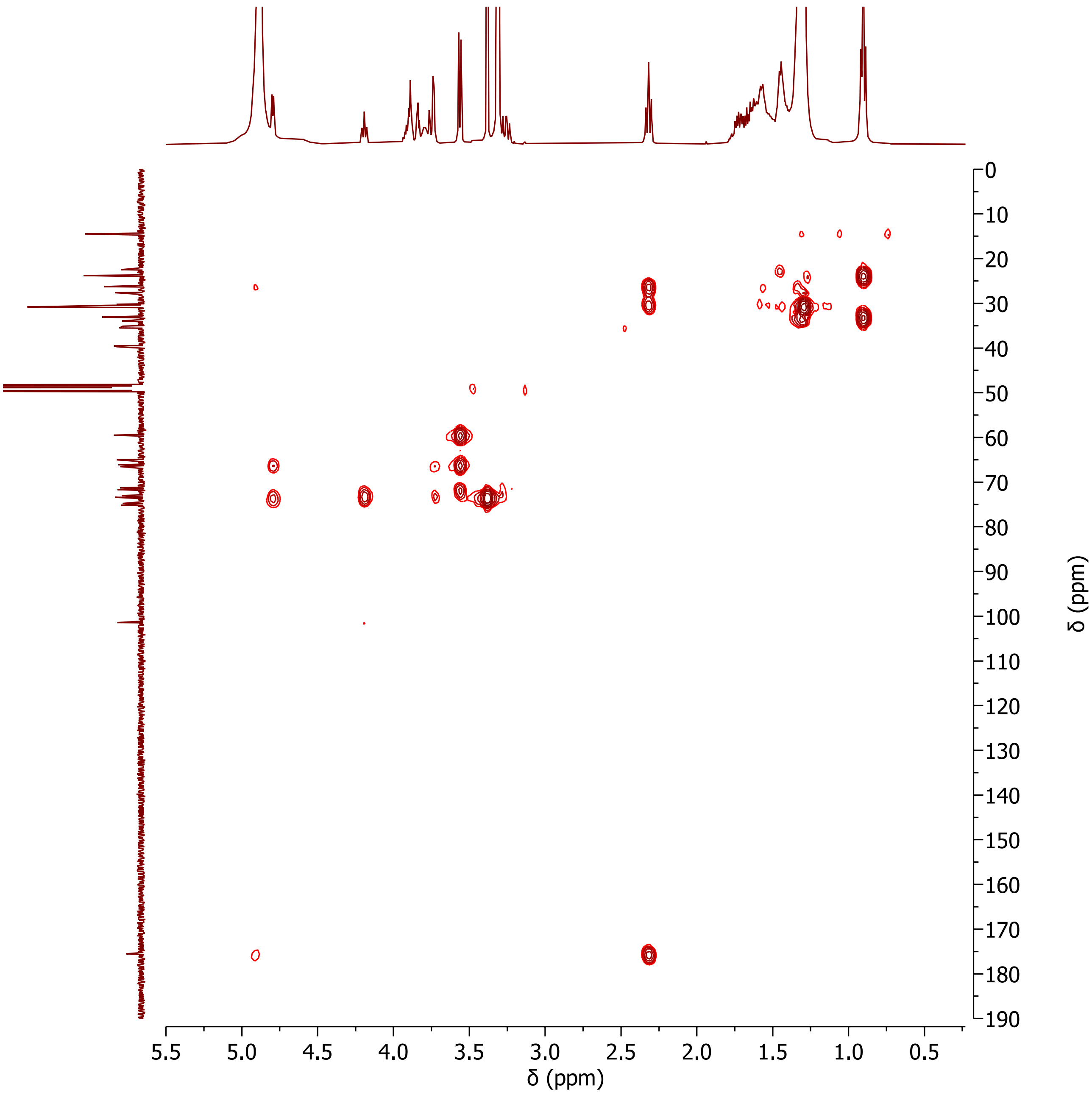

Supplementary Fig. 15 | ^1H–^{13}C HMBC spectrum of 7 in CD_3OD at 400 MHz

The coupling constant (cnst13) was 5 Hz.

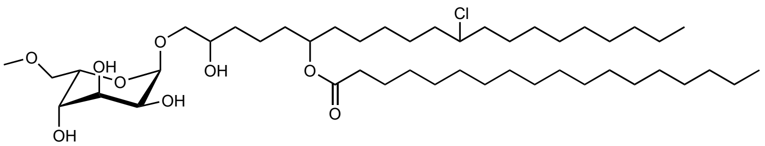

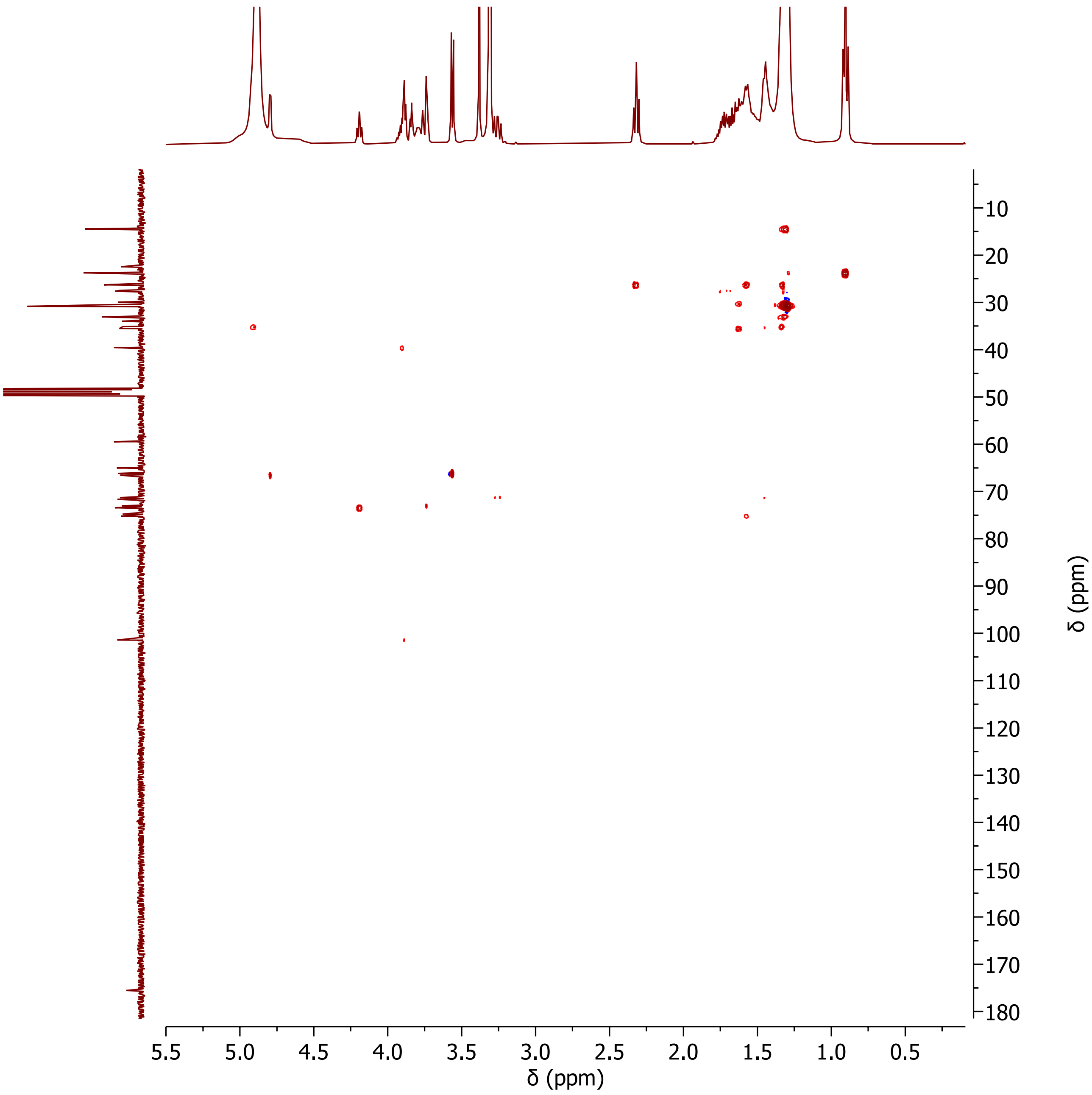

Supplementary Fig. 16 | ^{1}H–^{13}C H2BC spectrum of 7 in CD_3OD at 400 MHz

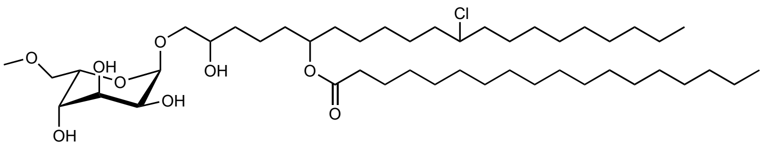

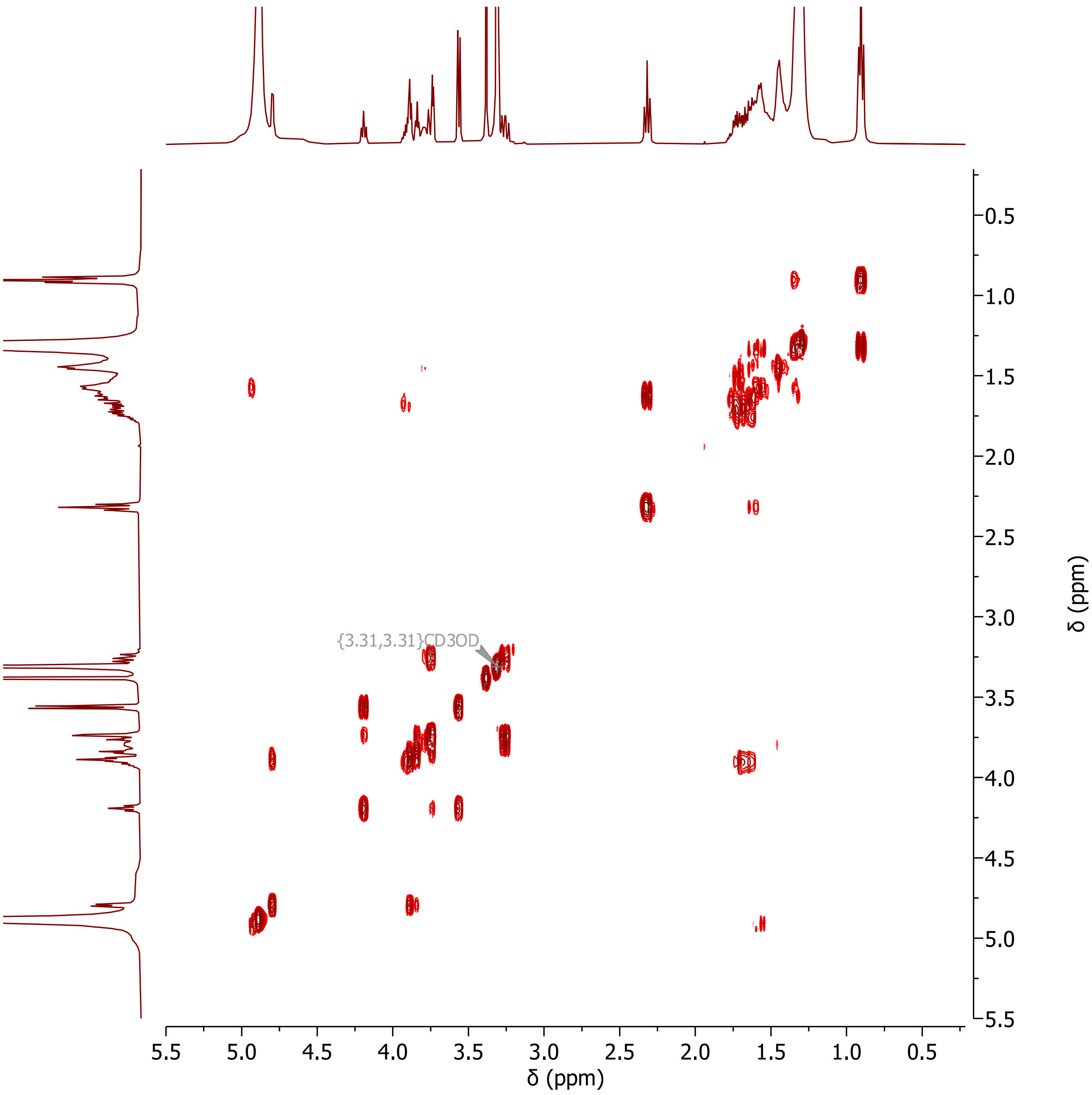

Supplementary Fig. 17 | ^{1}H–^{1}H COSY spectrum of 7 in CD_3OD at 400 MHz

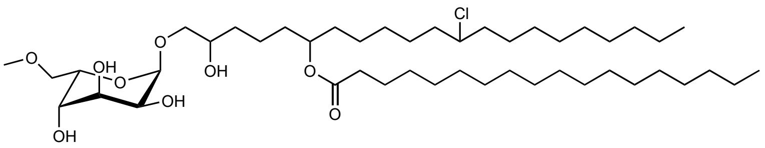

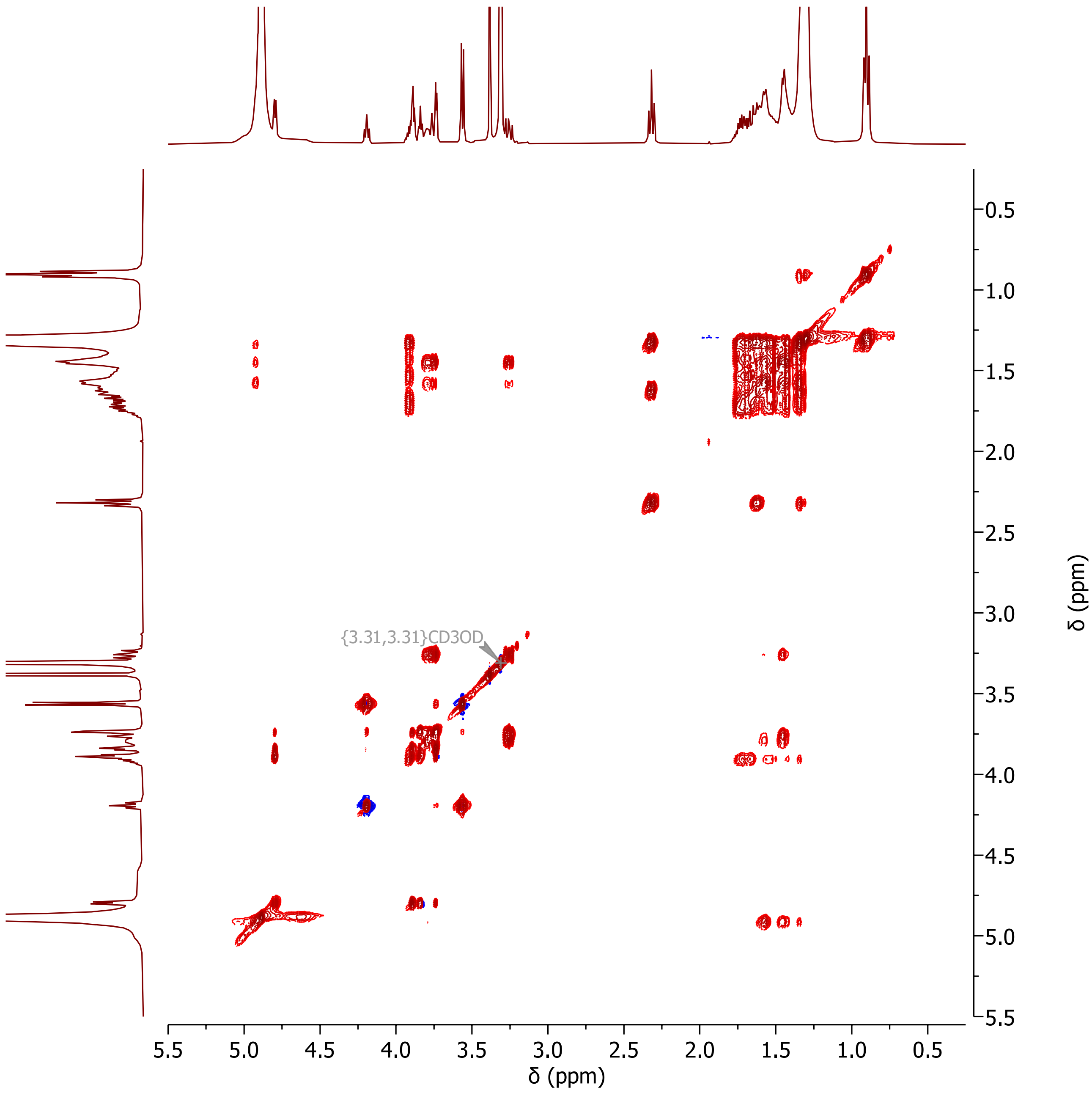

Supplementary Fig. 18 | ^{1}H–^{1}H TOCSY spectrum of 7 in CD_3OD at 400 MHz

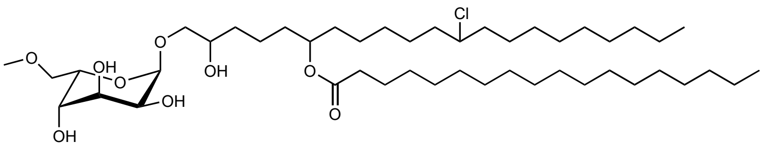

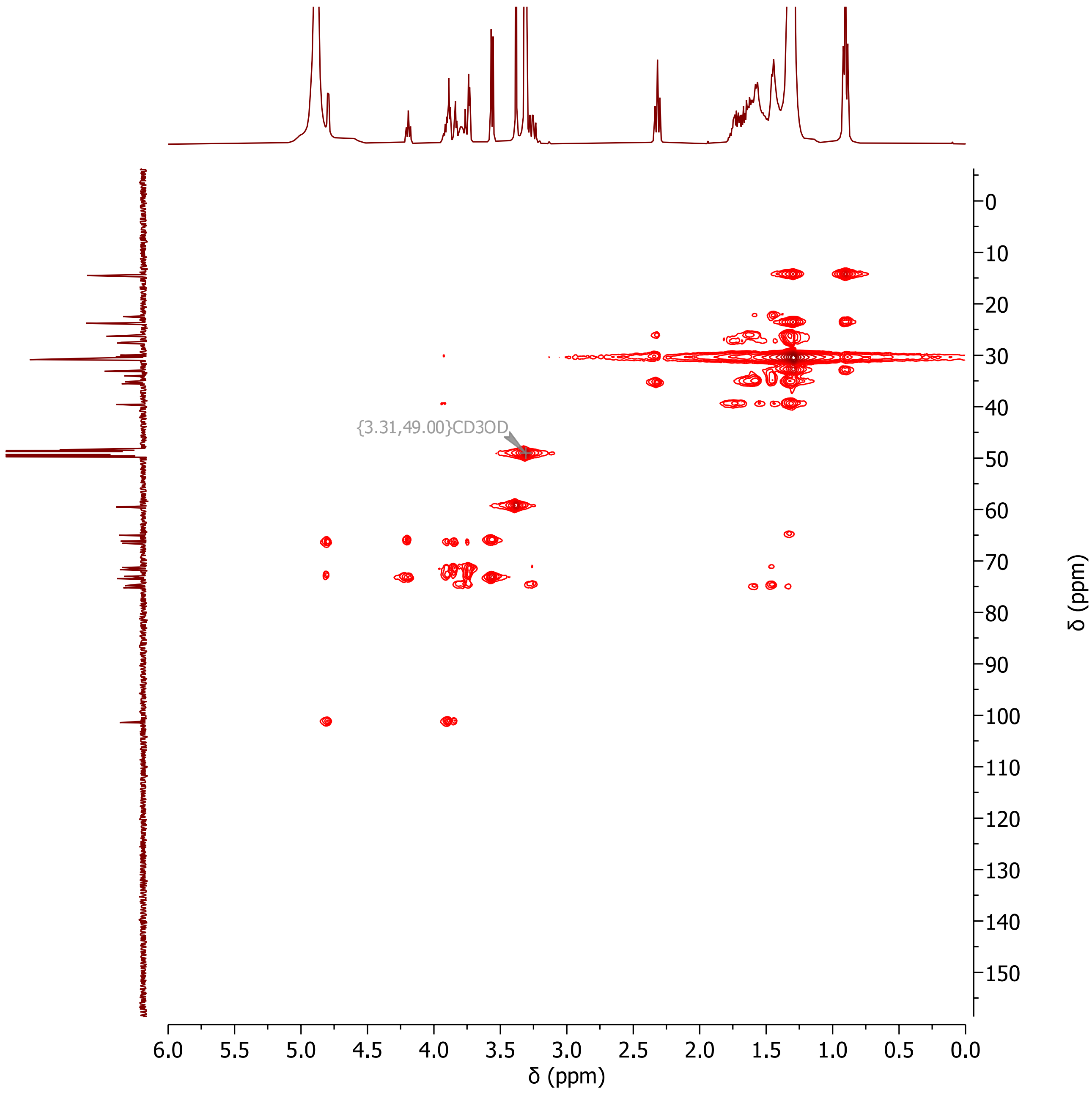

Supplementary Fig. 19 | $^{1}H–^{13}C$ HSQC-TOCSY spectrum of 7 in CD_3OD at 400 MHz

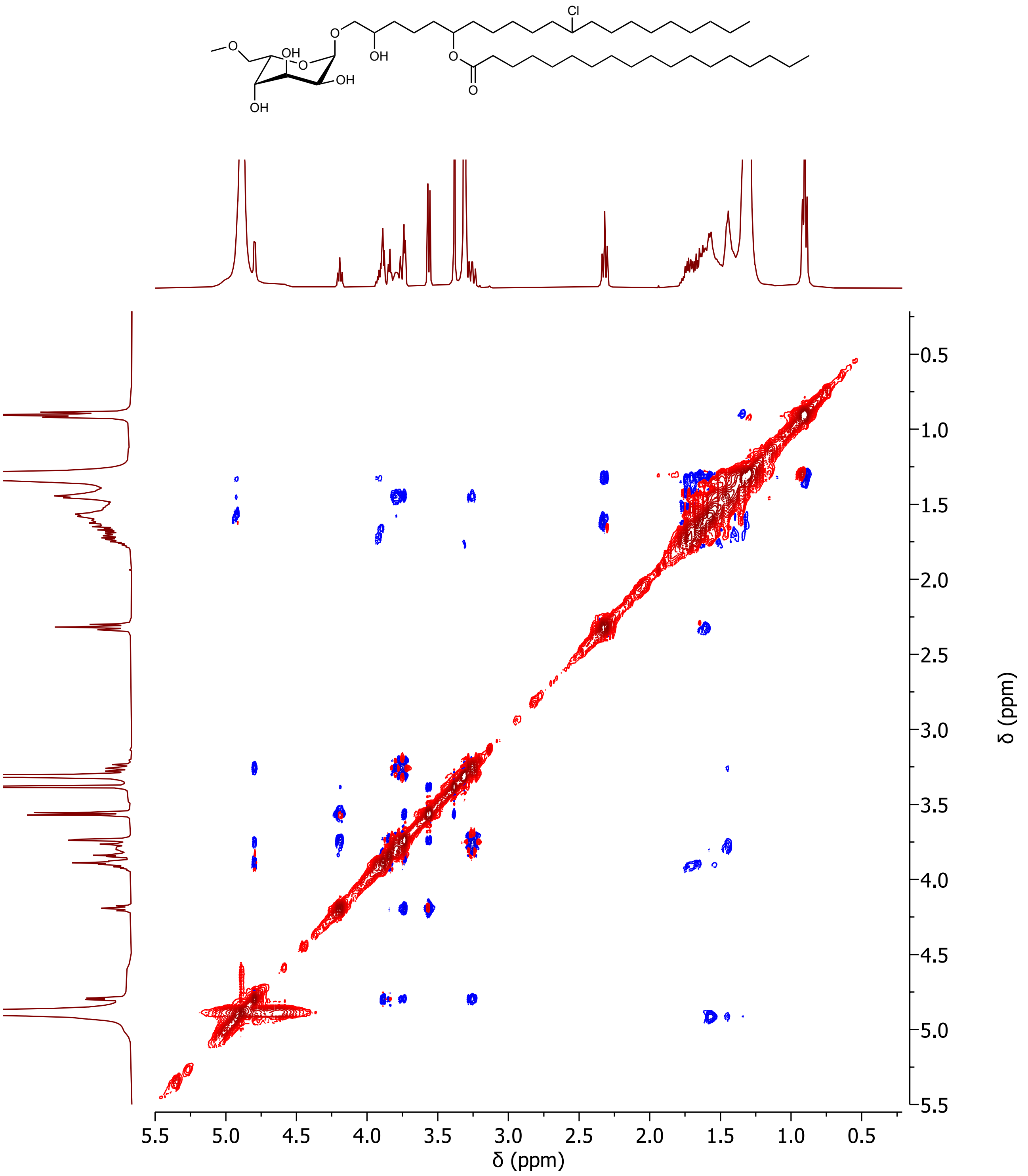

Supplementary Fig. 20 | ^{1}H–^{1}H NOESY spectrum of 7 in CD_3OD at 400 MHz

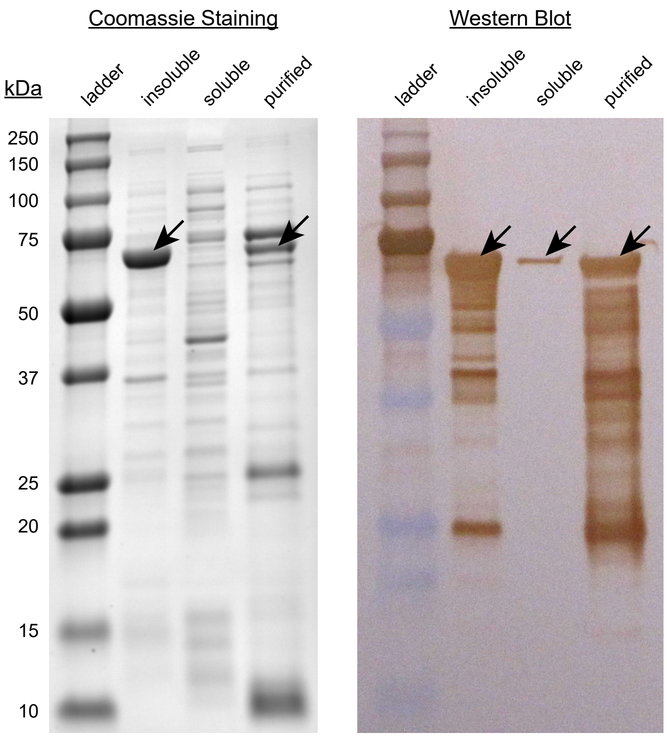

Supplementary Fig. 21 | SDS-PAGE gels illustrating purification of NgIO′

Arrows point to the band assigned to NgIO′. Gels were stained using Coomassie blue (left) or visualized by Western blotting (right) with a penta-His HRP conjugation kit (Qiagen). Representative lanes show the ladder ("ladder", Bio-Rad Precision Plus), insoluble protein in whole cell lysate ("insoluble"), soluble protein in whole cell lysate ("soluble"), and the isolated product (purified"). The many smaller bands in the purified product result from proteolysis of NgIO′ during purification. The purification was repeated once with similar results.

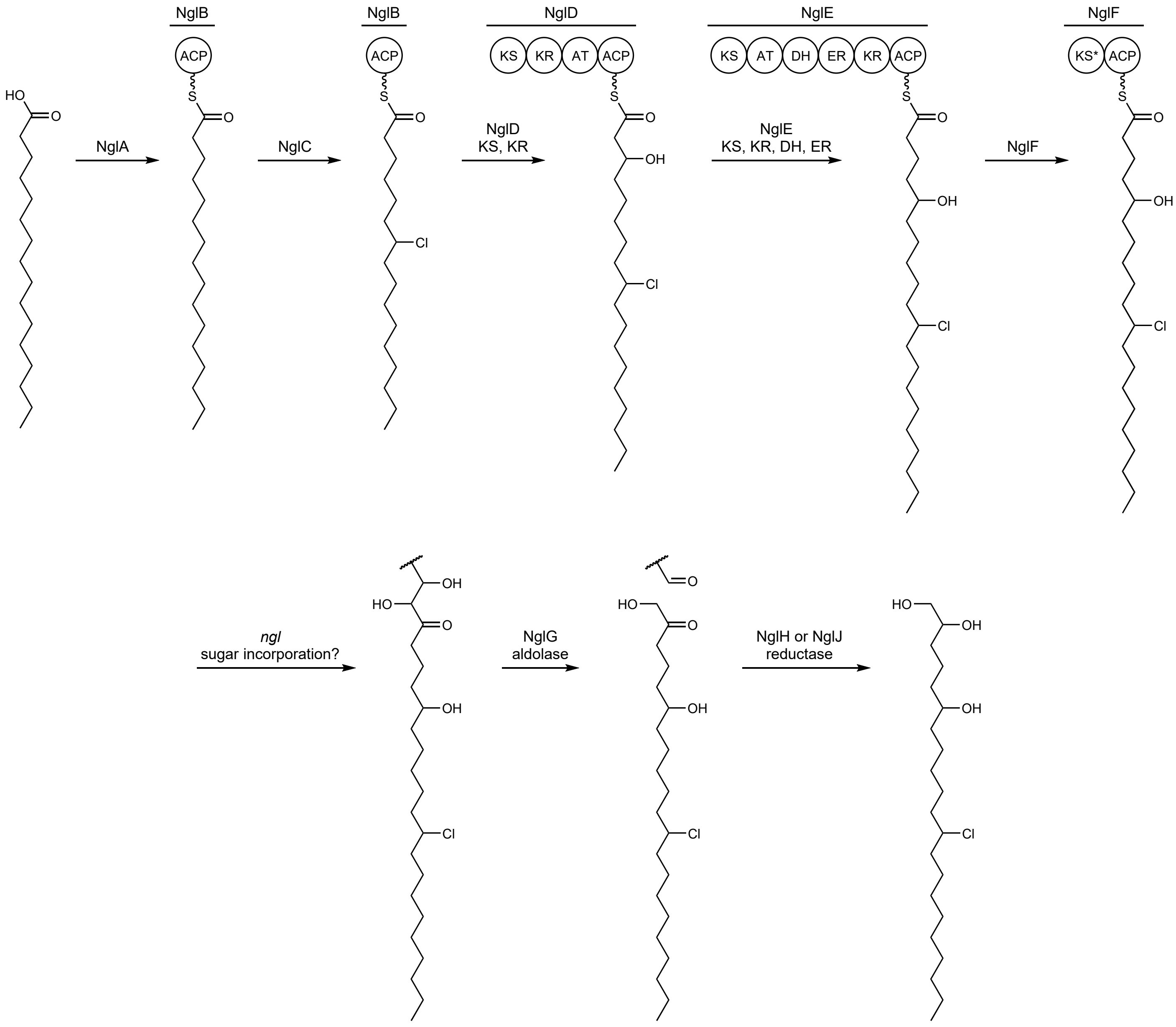

Supplementary Fig. 22 | Biosynthetic hypothesis for the acyl chain of 5

A possible extension of palmitic acid to create a C_{21} lipid tail.

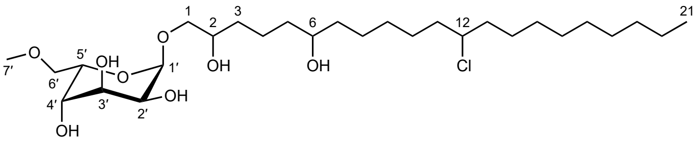

Position	δ ^{1}H (ppm)	m	J (Hz)	δ ^{13}C (ppm)
1A	3.27	dd	7.6, 9.9	74.8
1B	3.77	dd	3.0, 9.8	74.8
2	3.82	m		71.5
3	1.48	m		34.3
6	3.54	br		72.2
12	3.91	m		65.1
21	0.90	t	6.7	14.4
1′	4.80	d	4.0	101.4
2′	3.90	t	3.9	66.4
3′	3.85	td	3.8, 1.0	72.9
4′	3.73	dd	1.4, 3.8	71.7
5′	4.20	td	6.0, 1.4	66.1
6′	3.56	d	6.0	73.4
7′	3.38	s		59.5

Supplementary Table 6 | Partial NMR assignments for 5 in CD_3OD

The structure is shown above for reference. Unassigned aliphatic δ ^{13}C (ppm): 39.7, 39.6, 38.4, 38.3, 33.1, 30.7, 30.4, 30.3, 30.3, 27.6, 26.7, 23.7, 22.8. Unassigned aliphatic δ ^{1}H (ppm): 1.82–1.61 (5H, m), 1.60–1.40 (14H, br), 1.39–1.28 (15H, br).

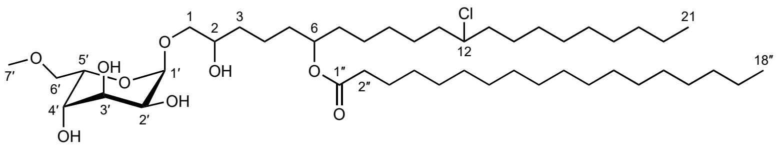

Position	δ ^{1}H (ppm)	m	J (Hz)	δ ^{13}C (ppm)
1A	3.26	dd	7.7, 10.1	74.8
1B	3.75	dd	3.1, 9.8	74.8
2	3.80	m		71.3
3	1.45	m		34.0
6	4.91	m		75.2
12	3.91	m		65.0
21	0.90	t	6.7	
1′	4.80	d	3.9	101.4
2′	3.89	t	3.9	66.5
3′	3.84	td	3.7, 1.0	73.0
4′	3.74	d	3.1	71.7
5′	4.19	td	5.9, 1.5	66.1
6′	3.56	d	5.9	73.4
7′	3.38	s		59.5
1″				175.5
2″	2.32	t	7.2	35.3
18″	0.90	t	6.7	

Supplementary Table 7 | Partial NMR assignments for 7 in CD_3OD

The structure is shown above for reference. Unassigned aliphatic δ ^{13}C (ppm): 39.7, 39.5, 35.5, 35.1, 33.1, 33.1, 30.8, 30.8, 30.7, 30.7, 30.7, 30.5, 30.5, 30.4, 30.3, 30.2, 29.9, 27.6, 27.5, 26.3, 23.8, 22.5, 14.5. Unassigned aliphatic δ ^{1}H (ppm): 1.81–1.53 (12H, m), 1.49–1.41 (6H, br), 1.37–1.27 (45H, br).

PDB	Description	GMQE	QSQE	Seq Id	Coverage	Range	Seq Similarity
2v9g.1.B	RHAMNULOSE-1-PHOSPHATE ALDOLASE	0.08	0	14.65	0.25	437-606	0.27
2v9g.1.A	RHAMNULOSE-1-PHOSPHATE ALDOLASE	0.08	0	14.65	0.25	437-606	0.27
1ojr.1.A	RHAMNULOSE-1-PHOSPHATE ALDOLASE	0.08	0.03	15.89	0.24	437-607	0.27
2v9o.1.A	RHAMNULOSE-1-PHOSPHATE ALDOLASE	0.09	0	13.61	0.24	436-607	0.27
2v9m.2.A	RHAMNULOSE-1-PHOSPHATE ALDOLASE	0.09	0	13.61	0.24	436-607	0.27
2v9m.1.A	RHAMNULOSE-1-PHOSPHATE ALDOLASE	0.08	0	13.61	0.24	436-607	0.27
2v2b.1.A	RHAMNULOSE-1-PHOSPHATE ALDOLASE	0.08	0	16.08	0.23	439-605	0.28
2v2a.1.A	RHAMNULOSE-1-PHOSPHATE ALDOLASE	0.08	0	16.08	0.23	437-606	0.27
1jdi.1.A	L-RIBULOSE 5 PHOSPHATE 4-EPIMERASE	0.1	0	22.31	0.21	444-600	0.32
1k0w.1.A	L-RIBULOSE 5 PHOSPHATE 4-EPIMERASE	0.1	0	20.77	0.21	443-599	0.31
2irp.1.A	Putative aldolase class 2 protein aq_1979	0.08	0	21.37	0.21	441-598	0.3
2uyv.1.A	RHAMNULOSE-1-PHOSPHATE ALDOLASE	0.06	0	16.18	0.22	439-598	0.27
3m4r.1.A	Uncharacterized protein	0.07	0	15.15	0.21	444-600	0.28
6btd.1.A	Fuculose phosphate aldolase	0.08	0	18.25	0.2	444-598	0.3
6voq.1.A	Aldolase	0.08	0	17.05	0.21	444-602	0.28
4m6r.1.A	Methylthioribulose-1-phosphate dehydratase	0.08	0	14.84	0.21	450-599	0.28
4m6r.1.D	Methylthioribulose-1-phosphate dehydratase	0.08	0	14.84	0.21	450-599	0.28
4c25.1.A	L-FUCULOSE PHOSPHATE ALDOLASE	0.07	0.03	16.67	0.2	444-598	0.29
1e4c.1.A	L-FUCULOSE 1-PHOSPHATE ALDOLASE	0.08	0.03	15.38	0.21	445-604	0.26
2opi.1.A	L-fuculose-1-phosphate aldolase	0.07	0	17.89	0.2	444-598	0.3
4c24.1.A	L-FUCULOSE PHOSPHATE ALDOLASE	0.07	0	15.08	0.2	443-598	0.28
3ocr.1.A	Class II aldolase/adducin domain protein	0.07	0	12	0.2	448-598	0.28
3ocr.2.A	Class II aldolase/adducin domain protein	0.06	0	12	0.2	448-598	0.28
6vop.1.A	Aldolase	0.08	0	19.01	0.2	444-598	0.3
1dzu.1.A	L-fuculose phosphate aldolase	0.08	0	17.6	0.2	445-599	0.27
1dzy.1.A	L-FUCULOSE-1-PHOSPHATE ALDOLASE	0.08	0.03	18.55	0.2	445-598	0.27
1dzx.1.A	L-fuculose phosphate aldolase	0.07	0.07	17.6	0.2	444-598	0.27
1e4b.1.A	L-FUCULOSE 1-PHOSPHATE ALDOLASE	0.08	0.07	18.55	0.2	445-598	0.27
1e47.1.A	L-FUCULOSE 1-PHOSPHATE ALDOLASE	0.08	0.07	17.6	0.2	444-598	0.27
1dzz.1.A	L-fuculose phosphate aldolase	0.08	0	18.55	0.2	445-598	0.27
1dzv.1.A	L-fuculose phosphate aldolase	0.08	0	17.74	0.2	445-598	0.27
1dzw.1.A	L-fuculose phosphate aldolase	0.08		17.74	0.2	445-598	0.27
1e4a.1.A	L-FUCULOSE 1-PHOSPHATE ALDOLASE	0.08		16.8	0.2	445-599	0.26
3fua.1.A	L-FUCULOSE-1-PHOSPHATE ALDOLASE	0.08		17.74	0.2	445-598	0.27
7x78.1.A	L-fuculose phosphate aldolase	0.08	0	16.94	0.2	445-598	0.27
1e49.1.A	L-FUCULOSE 1-PHOSPHATE ALDOLASE	0.08		16.94	0.2	445-598	0.27
1e48.1.A	L-FUCULOSE 1-PHOSPHATE ALDOLASE	0.08	0	18.85	0.2	445-599	0.28
1e46.1.A	L-FUCULOSE 1-PHOSPHATE ALDOLASE	0.08		19.83	0.2	445-598	0.28
2z7b.1.A	Mlr6791 protein	0.07	0	17.09	0.19	450-598	0.3
5t7z.1.A	EpoB	0.06	0	21.15	0.17	1-104	0.32
5t81.1.A	EpoB	0.06	0	21.15	0.17	1-104	0.32
5t81.2.A	EpoB	0.06	0	21.15	0.17	1-104	0.32
6cgo.1.A	Condensation domain protein	0.04		24.32	0.12	2-75	0.32
2jug.1.A	TubC protein	0.06	0.06	35.19	0.09	2-55	0.4
2jug.1.B	TubC protein	0.06	0.06	35.19	0.09	2-55	0.4
2jug.1.A	TubC protein	0.06	0.05	43.14	0.08	14-69	0.44
2jug.1.B	TubC protein	0.07	0.05	43.14	0.08	14-69	0.44
6ewv.1.A	NRPS Kj12C-NDD, NRPS Kj12B-CDD	0.03	0	19.3	0.09	1-57	0.34
6ewu.1.A	NRPS Kj12C-NDD	0.04	0	19.64	0.09	1-56	0.34
6ewt.1.A	NRPS Kj12B-NDD	0.04	0	21.43	0.09	1-56	0.34
6ews.1.A	NRPS Kj12A-NDD	0.04		19.64	0.09	1-56	0.33
3e02.1.A	uncharacterized protein DUF849	0		33.33	0.03	508-525	0.36
3e49.1.A	uncharacterized protein DUF849 with a TIM barrel fold	0		27.78	0.03	508-525	0.34
3c6c.1.A	3-keto-5-aminohexanoate cleavage enzyme	0		27.78	0.03	508-525	0.34

Supplementary Table 10 | Template search for homology modeling of NglG

The table shows select output from a homology template search using SWISS-MODEL[1].

Supplementary Note 1 | NMR analysis of 5 and 7

We began our structural elucidation by noting that the ^1H NMR spectrum of **5** shows a characteristic peak corresponding to a sugar anomeric proton at the 1′ position [δ_H 4.80 (1H, d)]. This was consistent with the presence of a predicted glycosyltransferase encoded by *ngl*. The sugar was established as a hexose by COSY correlations between H-1′ and H-2′ [δ_H 3.90 (1H, t)], H-3′ [δ_H 3.85 (1H, dt)] and H-4′ [δ_H 3.73 (1H, dd)], H-4′ and H-5′ [δ_H 4.20 (1H, td)], and H-5′ and H-6′ [δ_H 3.56 (2H, d)]. We interpret the apparent triplet at H-2′ as a doublet of doublets with similar *J*-couplings, and the apparent triplet at H-3′ as a doublet of doublets arising from long-range coupling in the constrained sugar ring. Although we could not resolve the expected COSY correlations between H-2′ and H-3′ because of overlap with their intense self-correlations, the correlations between H-2′ and C-3′ were observed in the H2BC spectrum. A methoxy group [δ_H 3.38 (3H, s)] showed an HMBC correlation to C-6′ indicating a 6-O-methyl hexose. The sugar was further identified as a hexopyranose by the HMBC correlation from H-5′ to C-1′. The NOESY correlation between the anomeric H-1′ and H-5′ was observable but extremely weak, indicating the sugar is present as the α-anomer.

An HMBC correlation between H-1 and C-1′ showed that the acyl chain is connected to the anomeric O-1′. The H-1 protons showed a large diastereotopic coupling between H_A-1 [δ_H 3.27 (1H, dd)] and H_B-1 [δ_H 3.77 (1H, dd)], likely originating from the chiral environment imposed by the adjacent sugar, and HSQC confirmed both are connected to C-1. COSY and H2BC correlations between both H-1 protons and H-2 [δ_H 3.82 (1H, m)] revealed a vicinal diol in the acyl chain. Phase information from HSQC showed that H-21 [δ_H 3.90 (3H, t)] is the only methyl group besides the sugar methoxy group. Based on the molecular formula and the elucidated sugar moiety, the acyl chain must therefore be a straight chain C_{21} lipid. We could not determine the locations of the protons corresponding to the remaining hydroxymethine [δ_H 3.54 (1H, br)] and chloromethine [δ_H 3.91 (1H, m)] from COSY, H2BC, or HMBC correlations.

The NMR spectra of **7** were virtually identical to that of **5**, with all of the same 2D correlations, consistent with the compounds being structurally related. The only notable difference is the appearance of α-carbonyl protons [δ_H 2.32 (2H, t)] corresponding to the ester linkage of an additional stearic acid moiety. In addition, the methine corresponding to the undetermined acyl chain hydroxy moves farther downfield in the ester derivative [δ_H 4.91 (1H, b)], with minimal change of other signals, and it shows an HMBC correlation with the α-carbonyl carbon, indicating it forms the ester in **7**.

Supplementary Note 2 | Isotopic labeling of 5

Based on the similarity of the biosynthetic machinery encoded by *ngl* to that involved in cylindrocyclophane production, namely the presence of a colocalized halogenase, acyl carrier protein (ACP), and FAAL (Fig. 3a), we inferred that biosynthesis of **5** likely begins with halogenation of a fatty acyl-ACP intermediate derived from a fatty acid precursor (Fig. 4d). We envisioned that observing the loss or retention of deuterium in products obtained upon feeding different deuterated fatty acid precursors could allow us to infer the identity of the fatty acid precursor and the position of chlorination. Feeding of decanoic-d_{19} acid to *N. punctiforme* led to incorporation of all 19 deuteria into **5**, whereas feeding lauric-d_{23} acid led to loss of one deuterium (Extended Data Fig. 5a). Incorporation into **5** with loss of 1 deuterium was also observed when feeding myristic-d_{27} acid and palmitic-d_{31} acid, but no incorporation was observed when feeding stearic-d_{35} acid. We did detect incorporation of one stearic-d_{35} acid unit into the second fatty acyl substituent of **7** with no loss of deuterium (Extended Data Fig. 5b), indicating that the lack of labeling in **5** was not because of limited solubility or cell permeability. These data suggest that palmitic acid is the starter unit for the biosynthesis of **5** because it is the largest fatty acid incorporated into the natural product backbone. We did not observe incorporation of palmitic acid into **6**, suggesting **6** arises from the incorporation of an unsaturated fatty acid instead of palmitic acid.

From these initial feeding studies, we concluded that the halogenase NglC (Npun_F3358) produces either a 6- or 7-chloropalmitic acyl-ACP thioester intermediate, which would give rise to chlorination at either the 11- and 12-positions of **5**, respectively (Fig. 4d). To distinguish between these possibilities, we synthesized palmitic-6,6-d_2 and palmitic-7,7-d_2 acid for feeding studies. Both deuteria were conserved in **5** when cultures were fed palmitic-6,6-d_2 acid (Fig. 4d). In contrast, one deuterium was lost in **5** when cultures were fed palmitic-7,7-d_2 acid (Fig. 4d). This result is consistent with the halogenase producing 7-chloropalmitic acid, which would result in chlorination at the 12-position of the final product (Fig. 4d). Together, the combination of NMR spectroscopy,

chemical derivatization, and stable isotope feeding experiments established the structures of **5** and **7** (Fig. 4a). From molecular networking, we propose that **8–11** are related to **7** but incorporate fatty acids varying in length and unsaturation (Fig. 4a).

Supplementary Note 3 | Biosynthetic hypothesis

We hypothesize that, similar to the cylindrocyclophanes, biosynthesis of **5** begins with extension of a C_{16} starter unit by the two PKS assembly line modules (NglD and NglE) to form a C_{20} unit (Supplementary Fig. 22). These PKSs contain the domains required to install two hydroxy groups at the observed locations in **5**. The function of the third PKS (NglF) is unclear because its ketosynthase domain contains a potentially inactivating active site mutation (H→Q at residue 344 to convert the conserved motif C-H-H to C-Q-H). A similar mutation has been previously shown to transform a ketosynthase into a transthiolase[2], and so we propose the third PKS offloads a biosynthetic intermediate for downstream elaboration. None of the three PKSs contain a thioesterase domain, and *ngl* does not encode a homolog of known trans-acting thioester reductases, suggesting *ngl* assembly line termination involves an uncharacterized mechanism. Although we have included a neighboring thioesterase [NglR (Npun_R3355)] in our annotation of *ngl* (Fig. 3a), thioesterase activity is insufficient to explain the PKS termination because the final product **5** does not contain a carboxylic acid or carboxylic acid derivative. Instead, we hypothesize the thioesterase serves a proofreading function and that on-pathway assembly line termination is performed by the hypothetical protein NglG (Npun_F3362). In support of this hypothesis, we note that a BLAST search of NglG reveals several hundred homologs that are encoded in additional PKS-encoding BGCs that lack known termination domains. NglG is predicted to contain a PKS-docking domain and predicted secondary structure conservation suggests it may also contain an aldolase domain (Supplementary Table 10). We therefore propose that the final C_{21} lipid with its unusual vicinal diol is formed from C–C bond cleavage of a larger PKS-derived product catalyzed by the putative aldolase domain (Supplementary Fig. 22). The larger product might arise from incorporation of a sugar moiety into the assembly line-derived product given the sugar-derivatizing enzymes encoded in *ngl* (Fig. 3a).

Synthetic Procedures

*Synthesis of methyl 6-oxohexadecanoate (**13**)*

6-Methoxy-6-oxohexanoic acid (0.500 g, 0.463 mL, 3.12 mmol, Acros Organics AC10282) was dissolved in 12 mL of anhydrous THF and triethyl amine (0.52 mL, 3.7 mmol, Sigma–Aldrich 471283) was added. In a separate flask, dichlorotriphenylphosphorane (1.455 g, 4.37 mmol, Sigma–Aldrich 378755) was dissolved in 12 mL anhydrous DCM and cooled to −10 °C. The 6-methoxy-6-oxohexanoic acid solution was added dropwise to the dichlorotriphenylphosphorane solution and stirred at −10 °C for 45 min. A solution of decylmagnesium bromide (3.12 mL of 1 M in diethyl ether, 3.12 mmol, Sigma–Aldrich 347108) was added dropwise over 45 min using a syringe pump and stirred for an additional 1.5 h at −10 °C under nitrogen. The reaction was quenched with 20 mL of aqueous 2 M HCl and extracted with three times with 20 mL of ethyl acetate. The combined organic layers were washed twice with 10 mL of saturated $NaHCO_3$ and once with 10 mL of brine, then dried over anhydrous sodium sulfate, filtered, and dried on a rotary evaporator. Triphenylphosphine oxide was removed by passing the crude product over a silica plug (50 mL of silica) in 90% DCM/10% acetone. After elution of the product, determined by TLC developed with 75% hexanes/25% acetone and stained with $KMnO_4$, the product was dried on a rotary evaporator. The product was further purified by flash chromatography (50 mL column) with 95% hexanes/5% ethyl acetate. Pure fractions were combined and dried on a rotary evaporator to afford methyl 6-oxohexadecanoate (**13**) (71.5 mg, 0.25 mmol, 8.1%). ^{1}H NMR ($CDCl_3$, 400 MHz): δ 3.67 (3H, s), 2.45–2.28 (6H, m), 1.66–1.58 (4H, m), 1.34–1.22 (14H, m, *J* = 2.9 Hz), 0.88 (3H, t, *J* = 6.8 Hz). ^{13}C NMR ($CDCl_3$, 101 MHz): δ 211.0, 173.9, 51.5, 42.9, 42.3, 33.9, 31.9, 29.6, 29.5, 29.4, 29.3, 29.3, 24.5, 23.9, 23.2, 22.7, 14.1. HRMS (*m/z*): calcd. for $C_{17}H_{33}O_3^+$ $[M + H]^+$, 285.2424; found, 285.2438.

Impure fractions were combined with a second synthesis of methyl 6-oxohexadecanoate that was performed at −40 °C and enriched by flash chromatography with 85% hexanes/10%

DCM/5% acetone. The combined material was purified by preparative TLC developed three times with 95% hexanes/5% ethyl acetate. Product was extracted from the silica using 80% DCM/20% acetone and dried on a rotary evaporator to afford impure methyl 6-oxohexadecanoate (56.8 mg, 74% purity by NMR with the major contaminant being another methyl ester).

*Synthesis of methyl 6-hydroxydecanoate-6-*d ***(14)***

Pure methyl 6-oxohexadecanoate (**13**) (70.6 mg, 0.248 mmol) was dissolved in 2 mL of anhydrous methanol and cooled to 0 °C. $NaBD_4$ (108.4 mg, 2.59 mmol, Acros Organics AC19495) was added over the course of 10 min and the reaction mixture was stirred on ice. After 30 min, additional $NaBD_4$ was added (20.0 mg, 0.478 mmol) and the reaction was stirred for 30 min. The reaction was quenched with 1 mL of aqueous 2 M HCl and extracted three times with 5 mL of DCM. The combined organic layers were washed once with 10 mL of saturated $NaHCO_3$ and once with 10 mL of brine, then dried over anhydrous sodium sulfate, filtered, and dried on a rotary evaporator. The product was purified by preparative TLC developed with 50% hexanes/40% diethyl ether/10% acetone. Product was extracted from the silica using 80% DCM/20% acetone and dried on a rotary evaporator to afford methyl 6-hydroxydecanoate-6-*d* (**14**) (53.5 mg, 0.186 mmol, 75%). The impure preparation of **13** (56.8 mg) was treated similarly to afford additional pure **14** (37.3 mg, 0.130 mmol). ^{1}H NMR ($CDCl_3$, 400 MHz): δ 3.67 (3H, s), 2.33 (2H, t, J = 7.5 Hz), 1.74–1.58 (2H, m), 1.51–1.33 (8H, m), 1.33–1.20 (16H, m), 0.88 (3H, t, J = 6.8 Hz). ^{13}C NMR ($CDCl_3$, 101 MHz): δ 174.2, 71.3 (t, J = 21.8 Hz), 51.5, 37.4, 36.9, 34.0, 31.9, 29.7, 29.6, 29.3, 25.6, 25.2, 24.9, 22.7, 14.1. HRMS (*m*/*z*): calcd. for $C_{17}H_{34}DO_3$ $[M + H]^+$, 288.2644; found, 288.2649.

*Synthesis of methyl 6-(tosyloxy)hexadecanoate-6-*d ***(15)***

Methyl 6-hydroxydecanoate-6-*d* (**14**) (90.8 mg, 0.316 mmol), *p*-toluenesulfonyl chloride (301 mg, 1.58 mmol, Sigma–Aldrich 240877), and 4-dimethylaminopyridine (9.7 mg, 0.079

mmol) were dissolved in anhydrous DCM and triethyl amine was added (0.44 mL, 3.2 mmol). The reaction mixture was stirred overnight at room temperature under nitrogen. The reaction was extracted with 10 mL of aqueous 1 M HCl and the aqueous layer was extracted with 8 mL of DCM. The organic layers were combined, washed once with 10 mL of saturated sodium bicarbonate, dried over anhydrous sodium sulfate, filtered, and dried on a rotary evaporator. The product was purified using preparative TLC developed with 65% hexanes/30% ether/5% acetone. Product was extracted from the silica using 80% DCM/20% acetone and dried on a rotary evaporator to afford methyl 6-(tosyloxy)hexadecanoate-6-*d* (**15**) (53.2 mg, 0.120 mmol, 38%). ^{1}H NMR ($CDCl_3$, 400 MHz): δ 7.81–7.76 (2H, m), 7.32 (2H, d, *J* = 8.2 Hz), 3.66 (3H, s), 2.44 (3H, s), 2.23 (2H, t, *J* = 7.5 Hz), 1.62–1.44 (7H, m), 1.36–1.07 (20H, m), 0.88 (3H, t, *J* = 6.9 Hz). ^{13}C NMR ($CDCl_3$, 101 MHz): δ 173.9, 144.4, 134.7, 129.7, 127.7, 83.7 (t, *J* = 22.5 Hz), 51.5, 34.0, 33.8, 33.7, 31.9, 29.6, 29.5, 29.4, 29.3, 29.3, 24.7, 24.6, 24.2, 22.7, 21.6, 14.1. HRMS (*m*/*z*): calcd. for $C_{24}H_{43}DNO_5S$ [M + NH_4]$^+$, 459.3000; found, 459.3017.

Synthesis of palmitic-6,6-d_2 *acid (**16**)*

Methyl 6-(tosyloxy)hexadecanoate-6-*d* (**15**) (45.4 mg, 0.103 mmol) was dissolved in 2 mL of DMSO. $NaBD_4$ (38.9 mg, 0.929 mmol) was added and the vessel was flushed with nitrogen. The reaction mixture was stirred for 4 hours at 80 °C with a vent to the atmosphere. The reaction was quenched with 1 mL of aqueous 2 M HCl and diluted with 10 mL of water. The product was extracted three times with 10 mL of DCM. The combined organic layers were extracted once with 10 mL of saturated $NaHCO_3$ and twice with water, dried over anhydrous sodium sulfate, filtered, and dried on a rotary evaporator to afford crude methyl hexadecanoate-6-6-d_2 (32.1 mg). The crude material was dissolved in 5 mL of 90% THF/10% aqueous 1 M LiOH and stirred vigorously overnight. The reaction was quenched with 1 mL of 2 M HCl and added to 10 mL of water. The product was extracted three times with 10 mL of DCM. The combined organic layers were

washed once with 30 mL of water, then dried over anhydrous sodium sulfate, filtered, and dried on a rotary evaporator. The product was purified using preparative TLC developed with 95% DCM/5% acetone. Product was extracted from the silica using diethyl ether and dried on a rotary evaporator to afford palmitic-6,6-d_2 acid (**16**) (5.0 mg, 0.019 mmol, 18%). ^{1}H NMR ($CDCl_3$, 400 MHz): δ 2.35 (2H, t, J = 7.5 Hz), 1.68–1.58 (2H, m), 1.37–1.23 (21H, m), 0.88 (3H, t, J = 6.9 Hz). ^{13}C NMR ($CDCl_3$, 101 MHz): δ 179.1, 33.9, 31.9, 29.7, 29.7, 29.7, 29.6, 29.4, 29.4, 29.0, 29.0, 24.7, 22.7, 14.1. HRMS (*m*/*z*): calcd. for $C_{16}H_{29}D_2O_2$ [M – H]$^-$, 257.2455; found, 257.2466.

*Synthesis of methyl 7-oxohexadecanoate (**17**)*

7-Methoxy-7-oxohexanoic acid (0.500 g, 0.456 mL, 2.87 mmol, Astatech A11369) was dissolved in 12 mL of anhydrous THF and triethyl amine (0.80 mL, 5.7 mmol, Sigma–Aldrich 471283) was added. In a separate flask, dichlorotriphenylphosphorane (1.91 g, 5.74 mmol, Sigma–Aldrich 378755) was dissolved in 12 mL anhydrous DCM and cooled to 0 °C. The dichlorotriphenylphosphorane solution was added to the 7-methoxy-7-oxohexanoic acid solution and the resulting mixture was stirred at 0 °C for 45 min. A solution of nonylmagnesium bromide (2.87 mL of 1 M in diethyl ether, 2.87 mmol) was added dropwise over 45 min using a syringe pump and the reaction mixture was stirred at 0 °C under nitrogen, then allowed to warm to room temperature and stirred overnight. The reaction was quenched with 20 mL of aqueous 2 M HCl and extracted with three times with 20 mL of ethyl acetate. The combined organic layers were washed twice with 10 mL of saturated $NaHCO_3$ and once with 10 mL of brine, then dried over anhydrous sodium sulfate, filtered, and dried on a rotary evaporator. Triphenylphosphine oxide was removed by passing the crude product over a silica plug (50 mL of silica) in 90% DCM/10% acetone. The product was further purified by flash chromatography (50 mL column) with 95% hexanes/5% ethyl acetate. Pure fractions were combined and dried on a rotary evaporator to afford methyl 7-oxohexadecanoate (**17**) (138.5 mg, 0.487 mmol, 17%). ^{1}H NMR ($CDCl_3$, 400

MHz): δ 3.66 (3H, s), 2.38 (4H, q, *J* = 7.7 Hz), 2.31 (2H, t, *J* = 7.5 Hz), 1.69–1.49 (6H, m), 1.36–1.22 (14H, m), 0.87 (3H, t, *J* = 6.8 Hz). ^{13}C NMR ($CDCl_3$, 101 MHz): δ 211.4, 174.1, 51.5, 42.9, 42.5, 33.9, 31.9, 29.5, 29.3, 28.7, 24.7, 23.9, 23.4, 22.7, 14.1. HRMS (*m/z*): calcd. for $C_{17}H_{33}O_3^+$ $[M + H]^+$, 285.2424; found, 285.2436.

*Synthesis of methyl 7-hydroxyhexadecanoate-7-*d ***(18)***

Methyl 7-oxohexadecanoate (**17**) (138.5 mg, 0.487 mmol) was dissolved in 4 mL of anhydrous methanol and cooled to 0 °C. $NaBD_4$ (237 mg, 4.87 mmol, Sigma–Aldrich 205591) was added over the course of 10 min and the reaction mixture was stirred at room temperature. After 1 h, additional $NaBD_4$ was added (237 mg, 4.87 mmol) and the reaction mixture was stirred for 30 min. The reaction was quenched with 10 mL of aqueous 2 M HCl and extracted three times with 10 mL of DCM. The combined organic layers were washed twice with 10 mL of saturated $NaHCO_3$ and once with 10 mL of brine, then dried over anhydrous sodium sulfate, filtered, and dried on a rotary evaporator. The product was purified by silica flash chromatography (50 mL column) with 80% hexanes/20% ethyl acetate. Pure fractions were combined and dried rotary evaporator to afford methyl 7-hydroxydecanoate-7-*d* (**18**) (74.4 mg, 0.259 mmol, 53%). ^{1}H NMR ($CDCl_3$, 400 MHz): δ 3.67 (3H, s), 2.31 (2H, t, *J* = 7.5 Hz), 1.64 (2H, p, *J* = 7.5 Hz), 1.49–1.31 (10H, m), 1.31–1.24 (13H, m), 0.88 (3H, t, *J* = 6.8 Hz). ^{13}C NMR ($CDCl_3$, 101 MHz): δ 174.3, 71.4 (t, *J* = 21.6 Hz), 51.5, 37.4, 37.1, 34.0, 31.9, 29.7, 29.7, 29.6, 29.3, 29.2, 25.6, 25.3, 24.9, 22.7, 14.1. HRMS (*m*/*z*): calcd. for $C_{17}H_{34}DO_3$ $[M + H]^+$, 288.2644; found, 288.2653.

*Synthesis of methyl 6-(tosyloxy)hexadecanoate-6-*d ***(19)***

Methyl 7-hydroxyhexadecanoate-7-*d* (**18**) (72.6 mg, 0.253 mmol), *p*-toluenesulfonyl chloride (482 mg, 25.3 mmol, Sigma–Aldrich 240877), and 4-dimethylaminopyridine (31.0 mg, 0.253 mmol) were dissolved in anhydrous DCM and triethyl amine was added (0.35 mL, 2.5 mmol). The reaction mixture was stirred overnight at room temperature under nitrogen. The

reaction was extracted with 10 mL of aqueous 1 M HCl and the aqueous layer was extracted with 8 mL of DCM. The organic layers were combined, washed once with 10 mL of saturated sodium bicarbonate, dried over anhydrous sodium sulfate, filtered, and dried on a rotary evaporator. The product was purified by silica flash chromatography (50 mL column) with 65% hexanes/30% diethyl ether/5% acetone. Pure fractions were combined and dried on a rotary evaporator to afford methyl 7-(tosyloxy)hexadecanoate-7-*d* (**19**) (46.5 mg, 0.105 mmol, 42%). ^{1}H NMR ($CDCl_3$, 400 MHz): δ 7.81–7.76 (2H, m), 7.33 (2H, d, *J* = 8.1 Hz), 3.67 (3H, s), 2.44 (3H, s), 2.25 (2H, t, *J* = 7.5 Hz), 1.62–1.48 (6H, m), 1.34–1.12 (19H, m), 0.88 (3H, t, *J* = 6.9 Hz). ^{13}C NMR ($CDCl_3$, 101 MHz): δ 174.1, 144.4, 134.7, 129.7, 127.7, 51.5, 34.0, 33.9, 33.9, 31.9, 29.5, 29.4, 29.3, 28.8, 24.7, 24.7, 24.4, 22.7, 21.6, 14.1. HRMS (*m*/*z*): calcd. for $C_{24}H_{43}DNO_5S$ $[M + NH_4]^+$, 459.3000; found, 459.3017.

*Synthesis of palmitic-7,7-d_2 acid (**20**)*

Methyl 7-(tosyloxy)hexadecanoate-7-*d* (**19**) (43.5 mg, 0.098 mmol) was dissolved in 5 mL of anhydrous DMSO. $NaBD_4$ (41.2 mg, 0.984 mmol) was added and the vessel was flushed with nitrogen. The reaction mixture was stirred overnight at 80 °C under nitrogen. The reaction was quenched with 10 mL of aqueous 2 M HCl and diluted with 10 mL of water. The product was extracted five times with 10 mL of DCM. The combined organic layers were extracted once with 10 mL of saturated $NaHCO_3$ and twice with 30 mL of water, dried over anhydrous magnesium sulfate, filtered, and dried on a rotary evaporator to afford crude methyl hexadecanoate-7-7-d_2. The crude material was dissolved in 5 mL of 90% THF/10% aqueous 1 M LiOH and stirred vigorously overnight. The reaction was quenched with 1 mL of 2 M HCl and added to 10 mL of water. The product was extracted four times with 10 mL of DCM. The combined organic layers were washed once with 30 mL of water, then dried over anhydrous magnesium sulfate, filtered, and dried on a rotary evaporator. The product was enriched by silica flash chromatography (10

mL column) with 90% hexanes/5% ethyl acetate/5% acetone, then purified by preparative TLC developed with 80% hexanes/10% ethyl acetate/10% acetone. The product was extracted from the silica with diethyl ether and dried on a rotary evaporator to afford palmitic-7,7-d_2 acid (**20**) (2.0 mg, 0.0077 mmol, 8%). ^{1}H NMR ($CDCl_3$, 400 MHz): δ 2.35 (2H, t, *J* = 7.5 Hz), 1.64 (2H, p, *J* = 7.4 Hz), 1.38–1.23 (21H, m), 0.88 (3H, t, *J* = 6.7 Hz). ^{13}C NMR ($CDCl_3$, 101 MHz): δ 177.55, 33.60, 31.95, 29.72, 29.68, 29.64, 29.46, 29.39, 29.25, 29.21, 29.09, 24.72, 22.72, 14.15. HRMS (*m*/*z*): calcd. for $C_{16}H_{29}D_2O_2$ [M – H]$^-$, 257.2455; found, 257.2466.

Supplementary References

1. Waterhouse, A., Bertoni, M., Bienert, S., Studer, G., Tauriello, G., Gumienny, R., Heer, F.T., de Beer, T.A.P., Rempfer, C., Bordoli, L., Lepore, R., Schwede, T. SWISS-MODEL: homology modelling of protein structures and complexes. *Nucleic Acids Res*. **46**(W1), W296-W303 (2018).

2. He, H.Y., Tang, M.C., Zhang, F., Tang, G.L. Cis-Double bond formation by thioesterase and transfer by ketosynthase in FR901464 biosynthesis. *J. Am. Chem. Soc.* **136**, 4488-4491 (2014).
