# Supplementary material for: Accelerating the discovery of alkyl halide-derived natural products using halide depletion
Source: Nat Chem. 2024 Jan 12;16(2):173–82. doi: 10.1038/s41557-023-01390-z (PMC10849952; doi:10.1038/s41557-023-01390-z)
Supplement: Supplementary file 2 — Reporting Summary [file 41557_2023_1390_MOESM2_ESM.pdf]

nature portfolio

Corresponding author(s): Emily Balskus

Last updated by author(s): Sep 8, 2022

Reporting Summary

Nature Portfolio wishes to improve the reproducibility of the work that we publish. This form provides structure for consistency and transparency in reporting. For further information on Nature Portfolio policies, see our Editorial Policies and the Editorial Policy Checklist.

Statistics

For all statistical analyses, confirm that the following items are present in the figure legend, table legend, main text, or Methods section.

n/a	Confirmed	
☐	☒	The exact sample size (*n*) for each experimental group/condition, given as a discrete number and unit of measurement
☐	☒	A statement on whether measurements were taken from distinct samples or whether the same sample was measured repeatedly
☐	☒	The statistical test(s) used AND whether they are one- or two-sided<br>*Only common tests should be described solely by name; describe more complex techniques in the Methods section.*
☒	☐	A description of all covariates tested
☒	☐	A description of any assumptions or corrections, such as tests of normality and adjustment for multiple comparisons
☐	☒	A full description of the statistical parameters including central tendency (e.g. means) or other basic estimates (e.g. regression coefficient) AND variation (e.g. standard deviation) or associated estimates of uncertainty (e.g. confidence intervals)
☐	☒	For null hypothesis testing, the test statistic (e.g. *F*, *t*, *r*) with confidence intervals, effect sizes, degrees of freedom and *P* value noted<br>*Give P values as exact values whenever suitable.*
☒	☐	For Bayesian analysis, information on the choice of priors and Markov chain Monte Carlo settings
☒	☐	For hierarchical and complex designs, identification of the appropriate level for tests and full reporting of outcomes
☒	☐	Estimates of effect sizes (e.g. Cohen's *d*, Pearson's *r*), indicating how they were calculated

Our web collection on statistics for biologists contains articles on many of the points above.

Software and code

Policy information about availability of computer code

Data collection	LC-MS data were collected using MassHunter v10.0.<br>NMR data were collected using TopSpin v4.0.9.
Data analysis	LC-MS data were analyzed using MassHunter Qualitative Analysis vB.07.00 (Agilent) and MZmine v2.53.<br>NMR data were analyzed using MestReNova v14.2.0-26256.<br>RNA-seq data were analyzed using Bowtie 2 v2.4.5 and featureCounts v2.0.3.<br>RNA-seq statistics were analyzed using DESeq2 v1.34.<br>Statistics tests were performed using Python v3.10.6 and SciPy v1.5.2.<br>Figures were plotted using Python v3.10.6, matplotlib v3.3.2, and seaborn v0.11.0.<br>Molecular networks were created using MetGem v1.3.6 and visualized using Cytoscape v3.7.2.

For manuscripts utilizing custom algorithms or software that are central to the research but not yet described in published literature, software must be made available to editors and reviewers. We strongly encourage code deposition in a community repository (e.g. GitHub). See the Nature Portfolio guidelines for submitting code & software for further information.

Data

Policy information about availability of data

All manuscripts must include a data availability statement. This statement should provide the following information, where applicable:

- Accession codes, unique identifiers, or web links for publicly available datasets
- A description of any restrictions on data availability
- For clinical datasets or third party data, please ensure that the statement adheres to our policy

RNA-seq data were deposited in the Sequence Read Archive (http://www.ncbi.nlm.nih.gov/sra) under accession number PRJNA868493. Raw LC–MS and LC–MS/MS data are available upon request due to large file sizes. Previously published crystal structures are available in the Protein Data Bank (https://www.rcsb.org/) under accession codes 7RON and 7ROO. All other data are available in the manuscript or Supplementary Information. Source data are provided with this paper.

Human research participants

Policy information about studies involving human research participants and Sex and Gender in Research.

Reporting on sex and gender	N/A
Population characteristics	N/A
Recruitment	N/A
Ethics oversight	N/A

Note that full information on the approval of the study protocol must also be provided in the manuscript.

Field-specific reporting

Please select the one below that is the best fit for your research. If you are not sure, read the appropriate sections before making your selection.

☒ Life sciences ☐ Behavioural & social sciences ☐ Ecological, evolutionary & environmental sciences

For a reference copy of the document with all sections, see nature.com/documents/nr-reporting-summary-flat.pdf

Life sciences study design

All studies must disclose on these points even when the disclosure is negative.

Sample size	Three independent biological replicates were analyzed for each experiment to ensure standard errors could be calculated, and because of the limited LC-MS time available and the cost of RNA-seq experiments. No statistical methods were used to predetermine sample size. The sample size was considered sufficient given the relatively low standard error compared to the large differences observed, and by validation with statistically independent follow-up experiments such as NMR and chemical derivatizations.
Data exclusions	One replication of the halide depletion experiment in Nostoc punctiforme ATCC 29133 was excluded because it became contaminated with an unknown species. However, the results of this contaminated experiment were consistent with the other findings of this study.
Replication	Experiments were replicated at least 3 times and yielded similar results.
Randomization	Randomization was not used because the study did not involve human or animal subjects, and it did not use statistical tests which required randomization. LC-MS carryover between samples was confirmed to be negligible by periodic blank injections throughout each run. All samples were treated identically.
Blinding	No blinding was used for LC-MS experiments because the study did not involve human or animal subjects, all samples were treated identically, and the results did not rely on subjective interpretation. Blinding was not possible for structural elucidation because it required multiple independent experiments whose results had to be interpreted in context of one another. The RNA-seq samples were measured by an outside organization that did not know the purpose of the experiments.

Reporting for specific materials, systems and methods

We require information from authors about some types of materials, experimental systems and methods used in many studies. Here, indicate whether each material, system or method listed is relevant to your study. If you are not sure if a list item applies to your research, read the appropriate section before selecting a response.

Materials & experimental systems

n/a	Involved in the study
☐	☒ Antibodies
☒	☐ Eukaryotic cell lines
☒	☐ Palaeontology and archaeology
☒	☐ Animals and other organisms
☒	☐ Clinical data
☒	☐ Dual use research of concern

Methods

n/a	Involved in the study
☒	☐ ChIP-seq
☒	☐ Flow cytometry
☒	☐ MRI-based neuroimaging

Antibodies

Antibodies used	Penta·His antibody from Qiagen (product number 34660, multiple lots) was used for Western blots.
Validation	The commercial Penta·His antibody was validated by the manufacturer.
